# Integration of molecular modelling and *in vitro* studies to inhibit LexA proteolysis

**DOI:** 10.3389/fcimb.2023.1051602

**Published:** 2023-03-03

**Authors:** Zachariah P. Schuurs, John P. McDonald, Laura V. Croft, Derek J. Richard, Roger Woodgate, Neha S. Gandhi

**Affiliations:** ^1^ Cancer and Ageing Research Program, Centre for Genomics and Personalised Health, Queensland University of Technology (QUT), Translational Research Institute (TRI), Brisbane, QLD, Australia; ^2^ School of Chemistry and Physics, Queensland University of Technology (QUT), Brisbane, QLD, Australia; ^3^ Laboratory of Genomic Integrity, National Institute of Child Health and Human Development, National Institutes of Health, Bethesda, MD, United States

**Keywords:** LexA, antibiotic resistance, covalent inhibitors, molecular docking, proteolysis

## Abstract

**Introduction:**

As antibiotic resistance has become more prevalent, the social and economic impacts are increasingly pressing. Indeed, bacteria have developed the SOS response which facilitates the evolution of resistance under genotoxic stress. The transcriptional repressor, LexA, plays a key role in this response. Mutation of LexA to a non-cleavable form that prevents the induction of the SOS response sensitizes bacteria to antibiotics. Achieving the same inhibition of proteolysis with small molecules also increases antibiotic susceptibility and reduces drug resistance acquisition. The availability of multiple LexA crystal structures, and the unique Ser-119 and Lys-156 catalytic dyad in the protein enables the rational design of inhibitors.

**Methods:**

We pursued a binary approach to inhibit proteolysis; we first investigated β-turn mimetics, and in the second approach we tested covalent warheads targeting the Ser-119 residue. We found that the cleavage site region (CSR) of the LexA protein is a classical Type II β-turn, and that published 1,2,3-triazole compounds mimic the β-turn. Generic covalent molecule libraries and a β-turn mimetic library were docked to the LexA C-terminal domain using molecular modelling methods in FlexX and CovDock respectively. The 133 highest-scoring molecules were screened for their ability to inhibit LexA cleavage under alkaline conditions. The top molecules were then tested using a RecA-mediated cleavage assay.

**Results:**

The β-turn library screen did not produce any hit compounds that inhibited RecA-mediated cleavage. The covalent screen discovered an electrophilic serine warhead that can inhibit LexA proteolysis, reacting with Ser-119 *via* a nitrile moiety.

**Discussion:**

This research presents a starting point for hit-to-lead optimisation, which could lead to inhibition of the SOS response and prevent the acquisition of antibiotic resistance.

## 1 Introduction

Antimicrobial resistance is a prevailing problem, threatening to undermine the progress of healthcare in the last century since the discovery of antibiotics. Infections that were previously treatable no longer respond to traditional antibiotics. Quiescent populations of bacterial pathogens resistant to antibiotics can lead to a higher risk of mortality from the infection and an increased risk of disease dissemination (Founou et al., 2017). Bacteria can acquire this resistance through three main mechanisms – transformation, transduction and conjugation (Munita and Arias, 2016). While some bacteria acquire resistance through genetic exchange, others acquire it as the result of chromosomal mutations that inactivate drug-activating enzymes or targets of the drugs. Where it has previously been unprofitable to develop new antibiotics, the social and economic implications are now beginning to outweigh that criterion (Aslam et al., 2018). It is therefore no surprise that alternative antibiotic targets, like the SOS pathway, have emerged (Cirz et al., 2005; Smith and Romesberg, 2007; Culyba et al., 2015).

**Figure 1:**
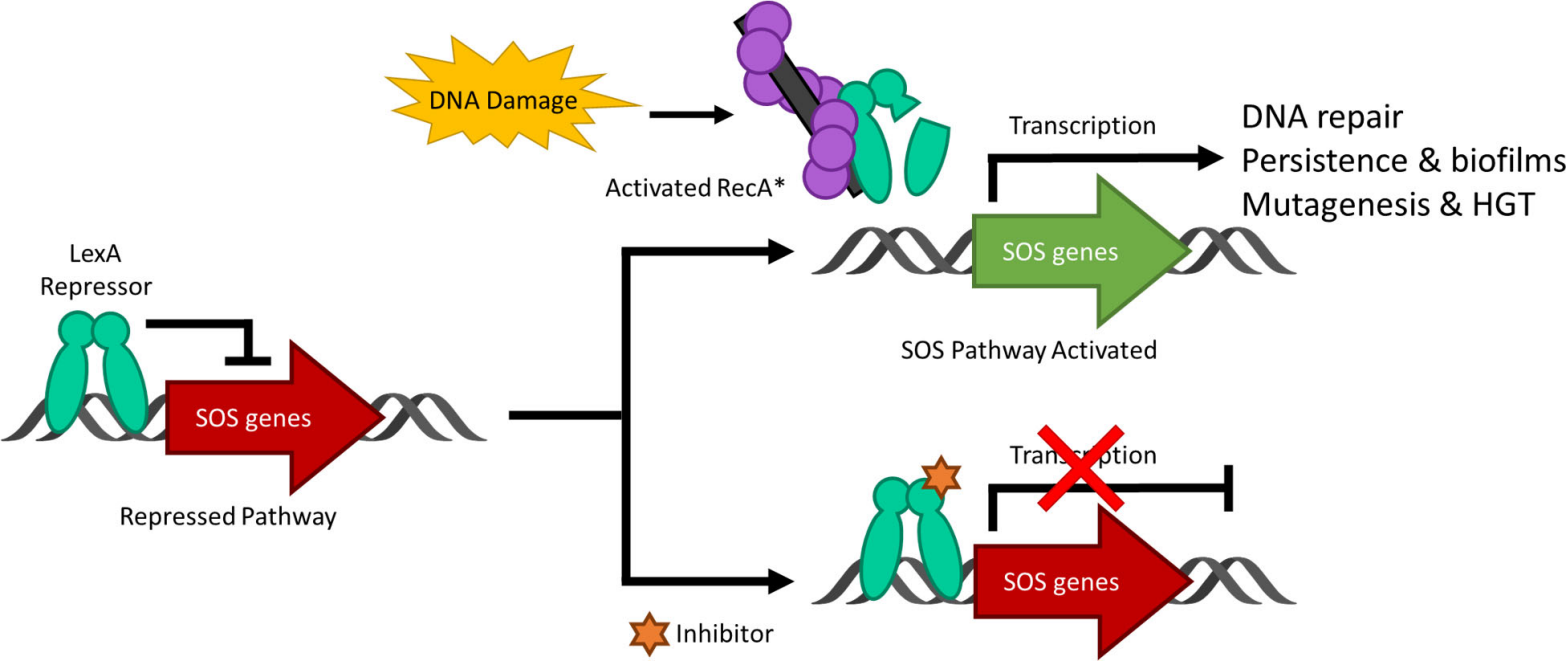
A schematic representing the bacterial SOS response to DNA damage and the inhibition of the pathway. Under standard conditions, the LexA dimer is bound to the promotor region repressing the transcription of the SOS response genes. Upon DNA damage and the activation of RecA to form nucleoprotein RecA* filaments, LexA undergoes proteolysis and derepresses the SOS genes as it is no longer able to bind to the promoter region. When a small molecule inhibitor is bound to LexA, the antibiotic-evasion associated pathway is antagonized and the SOS genes are not transcribed. Note that HGT stands for horizontal gene transfer.

**Figure 2:**
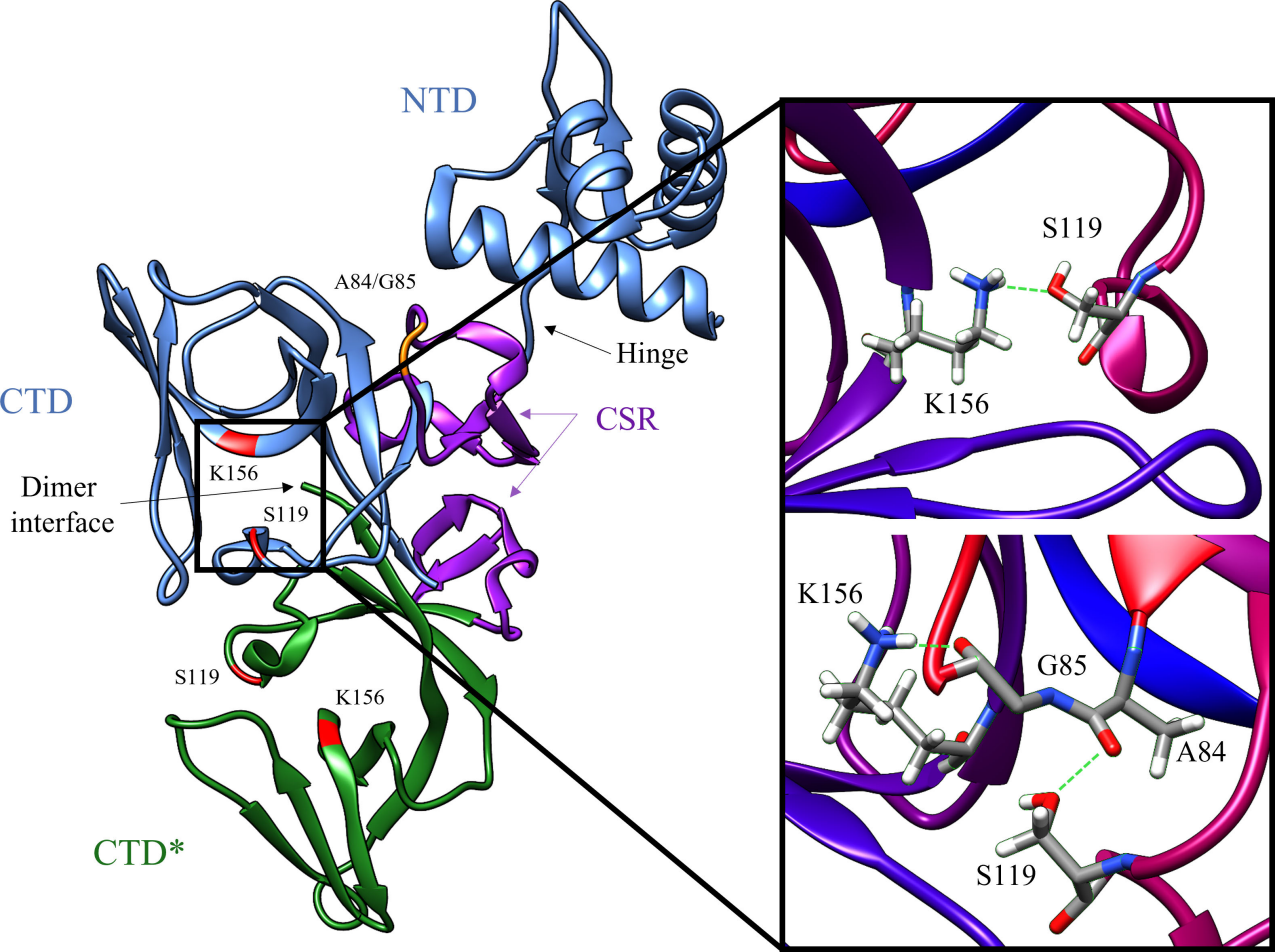
Key structural features of the LexA dimer (PDB ID: 1JHF). One of the monomers is depicted in blue, and the other is in green. The catalytic dyad Ser-119 and Lys156 are highlighted in red, and the scissile bond Ala-84 – Glu-85 is in orange. The CSR which undergoes the conformational shift upon binding to RecA is shown in purple. The insert depicts the transition from the catalytic S119-K156 dyad to the tetrahedral intermediate (PDB ID: 1JHE) as the scissile Ala-84 – Glu-85 moves into the hydrophobic pocket. This conformational change activates LexA from the NC form into the C form. The green dashed lines in the insert represent the hydrogen bonds between residues in the crystal structure. Note that the crystal structure does not contain the NTD of the second monomer in the dimer, due to poor electron density (Luo et al., 2001).

DNA damage to a bacterium compromises the chromosomal integrity and can threaten cell survival. As a countermeasure, bacteria have evolved the damage-inducible “SOS response” (Radman, 1974). The SOS response is regulated by two proteins; LexA, which serves as a transcriptional repressor of >40 genes in *E. coli* (Fernández de Henestrosa et al., 2000) and RecA, which upon DNA damage, forms a RecA nucleoprotein filament (RecA*) that mediates the self-cleavage of LexA. Upon proteolysis, LexA is inactivated as a transcriptional repressor and the SOS response is derepressed (**Figure 1**) (Little, 1984). The LexA NTD (**Figure 2**) normally binds to a 16-19 bp palindromic recognition site in the promoter region of genes in the SOS regulon (Fernández de Henestrosa et al., 2000). The affinity of LexA to this promoter region creates a finely tuned regulator of the SOS response. Genes that have weak LexA binding sites are induced first, while those with tighter binding sites are induced later in the SOS response (Fernández de Henestrosa et al., 2000). Of the more than 40 genes in the SOS regulation, three encode DNA polymerases (pols II, IV and V). In particular, polV is responsible for the majority of damage-induced mutagenesis in *E.coli* (Kato and Shinoura, 1977). Previous studies have reported that the SOS response can be attenuated by genetically inactivating the RecA*/LexA interaction (Mo et al., 2016) and antibiotic-associated mutagenesis is decreased, re-sensitizing resistant strains to DNA damaging antibiotics with the latter being dependent on functional pols II, IV and V (Cirz et al., 2006; Cirz et al., 2007; Li et al., 2010). As a consequence, previous studies have investigated the SOS pathway as target to prevent the acquisition of antibiotic resistance (Cirz et al., 2006; Cirz et al., 2007; Mo et al., 2018; Selwood et al., 2018; Bellio et al., 2020).

**Figure 3:**
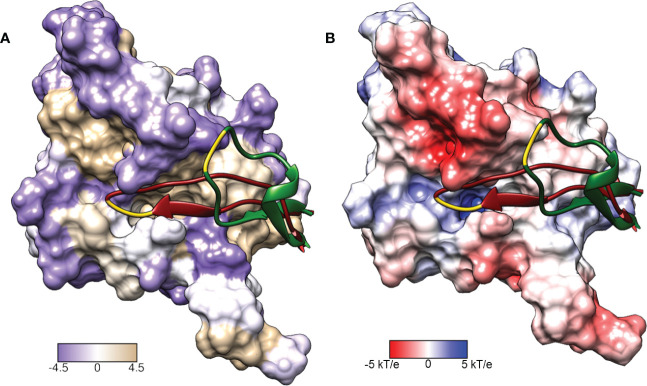
Surface representations of the LexA repressor protein. The ribbon represents the CSR in the cleavable (red, PDB: 1JHE) and non-cleavable (green, PDB: 1JHC) forms. The key scissile bond (Ala-84 – Gly-85) is in yellow. **(A)** depicts the amino acid hydrophobicity using the Kyte-Doolittle scale.**(B)** illustrates the electrostatic potential (kT/e) of the surface, calculated using DelPhi Webserver (Sarkar et al., 2013). These representations depict the catalytic pocket that the CSR moves into when the protein is activated. As the surface representations indicate, the pocket is highly hydrophobic and electrostatically positive.

LexA is a dimeric protein in solution (Mohana-Borges et al., 2000), with each monomer joined by a short flexible linker called the cleavage site region (CSR) between residues 79-95 (**Figure 2**). This linker region undergoes a conformational change from the non-cleavable (NC) form to the cleavable (C) form when LexA binds to RecA*. **Figure 3** illustrates the conformational movement of the CSR. The CSR region of LexA has been described as a β-turn in several instances (Mo et al., 2014; Jaramillo et al., 2022), but is not yet categorised. The N-terminal domain (NTD) binds to the LexA-binding box, while the C-terminal domain contains the protease active site. LexA is a serine protease, in the endopeptidase clan SF, and part of family S24 (Slilaty and Little, 1987; Polgár, 2013) defined by the Ser-119 and Lys-156 catalytic dyad (**Figure 2**). Located in a hydrophobic cleft (**Figure 3**), the Ser-119 of the dyad acts as a nucleophile and the lysine as the acid/base. Over the pH range 7.15-11.77 LexA, undergoes a linear rate of autodigestion, reaching a plateau above pH 10 (Slilaty et al., 1986). In this case, the Lys-156 is deprotonated, and the protein undergoes autocleavage (Slilaty and Little, 1987). In the presence of RecA* nucleoprotein filaments, the CSR loop changes conformation, shifting into the hydrophobic cleft (**Figure 3**) and causing the pK_a_ of the Lys-156 to change, deprotonating it (Polgár, 2013). This forms a transient tetrahedral intermediate between the Ser-119, Ala-84 and Gly-85 (**Figure 2** insert). Cleavage of the protein occurs when the bond between Ala-84 and Glu-85 is hydrolysed by the nucleophilic Ser-119.

LexA was the first of a superfamily of enzymes that have been shown to undergo autoproteolysis (Little, 1984; Slilaty et al., 1986; Burckhardt et al., 1988; McDonald et al., 1999; Luo et al., 2001; Cezairliyan and Sauer, 2009; Gonzalez et al., 2019). This family of enzymes are subjected to RecA-mediated cleavage and are therefore close structural homologs to LexA. This includes UmuD (Burckhardt et al., 1988; McDonald et al., 1999), DinR (Haijema et al., 1996; Winterling et al., 1997), SetR_ICE391_ (Gonzalez et al., 2019) and λ c*I* (Gimble and Sauer, 1985), all of which are involved in the mutagenic SOS response. The scissile Ala-Gly or Cys-Gly residues, along with the catalytic dyad of Ser and Lys are conserved across these proteins. While all of them undergo proteolysis, what sets them apart is the rate at which they undergo cleavage. Point mutations of the residues around the CSR have found that they are essential to mediating the rate at which members of this family undergo cleavage, from speeding it up to preventing cleavage altogether (Gimble and Sauer, 1985; Lin and Little, 1988; Shepley and Little, 1996; McDonald et al., 1998; Beuning et al., 2006; Mo et al., 2014).

While prior work has attempted to develop inhibitors to LexA cleavage, none are potent enough to take to market. A major campaign in a partnership between industry and academia (Mo et al., 2018) reported five molecules with micromolar cytotoxicity. The 1,2,3-triazole lead (GSK-C1) from this first iteration was advanced by Selwood et al. (Selwood et al., 2018) and an analogue of the initial hit was reported to have an IC50 of 9 μM against LexA. Further modifications to the 1,2,3,-triazole core scaffold have been investigated by Jaramillo et al. (Jaramillo et al., 2022), however, none of the compounds had an IC50 below 48 μM. To aid in the discovery of novel inhibitors, an understanding of these compounds’ molecular recognition is necessary. Molecular modelling has proven to be a vital tool in the determination of a structure-activity relationship (SAR) without the need to perform resource-intensive mutagenesis studies (dos Santos et al., 2014). These approaches have also facilitated the ability to screen large chemical spaces in a relatively short amount of time and at little cost compared to their counterpart *in vitro* screening techniques (Bender et al., 2021). A common approach to novel proteins is to virtually screen large libraries of compounds, sometimes reaching into millions of compounds. When there are existing compounds known to bind to a protein it can be appropriate to use them as a rationale to perform a targeted screen of smaller libraries (Sarnpitak et al., 2015). This reduces the computational resources required and speeds up how long the screening campaign takes.

In recent times, covalent inhibitors have gained momentum owing to their potency, selectivity and extended duration of action (Kim et al., 2021; Gao et al., 2022). Covalent inhibitors have an electrophilic warhead that reacts with nucleophilic residues, both reversibly and irreversibly (Kumalo et al., 2015; Ábrányi-Balogh and Keserű, 2022). Selectivity is achieved through non-covalent interactions with the scaffold. Serine is one of the most commonly targeted residues due to its catalytic role in many proteases, and common covalent inhibitors such as penicillin and aspirin are examples of serine warheads (Martin et al., 2019). A recent study explored boronic acids as covalent inhibitors of LexA autocleavage (Bellio et al., 2020). This study found (3-aminophenyl)boronic acid had a K_d_ of 1.07 mM, and that it is predicted to form an acyl-enzyme intermediate with the Ser-119 and form hydrogen bonds with the Lys-156 and Val-153 (Bellio et al., 2020). The same study developed a new equation for describing the inhibition potency of autoproteolytic enzymes (Bellio et al., 2020).

This investigation uses a two-fold approach to find a novel inhibitor scaffold. First, we establishedthe SAR of current non-covalent inhibitors, which was used as a rationale to dock a selected molecule libraries – β-turn mimetics and covalent serine warheads. A small library of the top binding molecules from each of the two libraries was screened *in vitro* after establishing a pseudo high-throughput approach. A K_d_ for the identified covalent proteolytic antagonist was determined, and the unsuccessful nature of the β-turn mimetic screen was discussed. Herein, we describe the discovery of a covalent inhibitor scaffold that inhibits the cleavage of the LexA transcriptional repressor.

## 2 Materials and methods

### 2.1 Hydrophobic and electrostatic potential surface

The hydrophobicity of LexA (**Figure 3A**) was represented according to the Kyte and Doolittle scale (Kyte and Doolittle, 1982) which assigns a hydrophobicity value to each residue, and was visualised with UCSF Chimera version 1.13.1 (Pettersen et al., 2004). The Adaptive Poisson-Boltzmann Solver (APBS) (Jurrus et al., 2018) webserver was used to calculate the electrostatic potential surface of the LexA CTD wild-type monomer. The C-terminal wild type (WT) was prepared by downloading the structural file (PDB: 1JHC) and mutating the Ala-119 to a serine before adding hydrogens. The PDB file was converted to PQR format at pH 7.0 and using PROPKA (Olsson et al., 2011; Søndergaard et al., 2011) to assign protonation states. The PARSE forcefield (Sitkoff et al., 1994; Tang et al., 2007) was used for the conversion. To run APBS, the dielectric constant set to 2.00 Fm^-1^, and the solvent dielectric constant to 78.5400 Fm^-1^. The output electrostatic potential surface was visualised with UCSF Chimera version 1.13.1 (Pettersen et al., 2004) (**Figure 3B**).

### 2.2 Molecular docking with BioSolveIT

The mutant *E. coli* LexA C-terminal fragment (PDB ID: 1JHC) (Luo et al., 2001) was obtained from the Protein Data Bank (Berman et al., 2000). The A119 presented in the 3D structure was changed to serine using Chimera version 1.13.1 (Pettersen et al., 2004) to represent the WT LexA sequence. As there are no structures of LexA co-crystallized with ligands, a comparative assessment of docking programs could not be conducted. We used the FlexX (Rarey et al., 1996) module of SeeSAR version 10.1 (BioSolveIT GmBH, 2020) to dock the three molecules from Mo et al. that bound directly to LexA (Mo et al., 2018). We docked these three to determine their binding modes and use them as a point of comparison (**Figure S1**). HYDE (Reulecke et al., 2008) was used to re-score the docked poses and calculate predicted binding affinities. This scoring function involves the fragmentation of the ligand and selection of a base fragment, which is placed in the active site. A free energy is assigned to each atom depending on the emerging hydrogen bond and dehydration energies of the complex. A tree-search algorithm is then used to incrementally build upon the base fragment. SeeSAR (BioSolveIT GmBH, 2020) was used as it offers a unique suite of tools to understand binding recognition from per-atom scoring and overall concentration ranges for the ligand dissociation constant (Pagadala et al., 2017). The analysis of the β-turn in LexA was accomplished using RamachanDraw v0.2.3 (Cirilo, 2022) and visualised using BIOVIA Discovery Studio version 10.1.0.19295 (Biovia, 2019).

### 2.3 Virtual Screening of β-turn mimetics with BioSolveIt

Having determined the type of β-turn present in the LexA structure, we theorised that the catalytic site would molecularly recognise β-turn mimetics. A structure-based virtual screening was performed with the ChemDiv library peptidomimetics of β-turn motifs (ChemDiv, 2019). The 2276 molecules were docked to the full-length, wild-type LexA CTD protein using FlexX (Rarey et al., 1996). The binding site was automatically identified by SeeSAR (BioSolveIT GmBH, 2020), matching the predicted hydrophobic cleft. Each ligand was docked with 10 poses per molecule generated, and a and a score was generated for each pose using the HYDE scoring function (Schneider et al., 2013). The top 53 compounds were subsequently ordered from ChemDiv to test *in vitro*. Docking poses were visualised using Chimera version 1.13.1 (Pettersen et al., 2004) and 2D residue interaction plots were generated with BIOVIA Discovery Studio version 20.1.0.19295 (Biovia, 2019).

### 2.4 Virtual screening of covalent inhibitors with CovDock

The ChemDiv libraries Covalent Generic and Smart Inhibitors (ChemDiv, 2020) was docked to the WT LexA using the CovDock (Toledo Warshaviak et al., 2014; Zhu et al., 2014) workflow in the Schrödinger Maestro suite 2019-4. This was used as Maestro Suite is considered to have one of the best high-throughput covalent docking implementations (Kumalo et al., 2015; Scarpino et al., 2018). The WT LexA protein structure was prepared using the Schrodinger protein preparation wizard, and the 8607 molecules were prepared using LigPrep (Schrodinger, 2020). The OPLS3e forcefield (Roos et al., 2019) was used to dock both compound libraries. The cubic grid used to select the search space, with an inner box equal to 15 Å and outer box of 25 Å. The grid box was centred on the catalytic Ser-119, which was selected as the active residue. CovDock automatically sorts the input library based on the possible covalent reactions between the warhead functional groups and the selected catalytic residues. The sub libraries were docked with beta-lactam addition; boronic acid addition; conjugate addition to an alkyne (aryl and carbonyl activated), alkene (nitrile activated); epoxide opening, Michael addition, nucleophilic addition to a double and triple bond and nucleophilic substitution. The CovDock module consists of five distinct steps (Zhu et al., 2014). ConfGen (Watts et al., 2010) generated conformations of each molecule, and the three conformations with the lowest conformational energies were selected for Glide docking. CovDock mutated the Ser-119 to an alanine, removing potential interference of the sidechain, and the molecules are docked within 8 Å of the C-beta atom of the S119A. Poses of each molecule scored within 2.5 kcal/mol of the lowest score sampled were retained. Secondly, the Ser-119 was restored, and the side chain conformations were sampled with a rotamer library. The binding modes from step one was sampled to see if the two atoms that would form the covalent bond were within 5 Å of each other. If so, the covalent bond was then formed, and all the changes in bond order, ionization state and chirality were adjusted. Stereoisomers were retained for further optimisation. Next the complexes were minimised in a vacuum to restore standard bond lengths and avoid steric clashes. The molecule cartesian coordinates were clustered with a k-means algorithm and poses were selected and minimised to obtain a Prime energy used to rank poses and select favourable binding geometry. Lastly, the poses were assigned a docking score based on the empirical scoring function that Zhu et al. developed. This score is the average of the pre-reactive Glide score and the Glide score of the ligand in the final complex. This score aims to capture the key elements during the covalent docking process (Zhu et al., 2014; Delre et al., 2020). Of the 8607 input compounds, the top scoring compounds across the reaction types were ranked by docking score and analyzed visually. The 80 top scoring compounds were ordered from ChemDiv (San Diego, CA).

### 2.5 Expression and purification of native *E.coli* LexA and RecA proteins

Native, untagged LexA protein, was expressed from an IPTG-inducible T7 promoter from plasmid pJWL288 (Roland et al., 1992) in the *E.coli* strain, RW644 (Karata et al., 2012). LexA was purified to more than 95% purity by standard protocols (Little, 1984). Native, untagged RecA, protein was expressed from an IPTG-inducible T7 promoter from plasmid pAIR79 (Lusetti et al., 2003) in the *E.coli* strain, EAW68 (Norais et al., 2009), that was also transformed with pT7pol26 (Mertens et al., 1995). RecA was purified to greater than 95% purity by standard protocols (Cox et al., 1981; Lusetti et al., 2003; Ojha et al., 2022).

### 2.6 *In vitro* screen LexA autocleavage reactions

A total of 133 compounds from the two screens were ordered from ChemDiv (San Diego, CA), which consisted of 80 molecules from the covalent screen, and 53 molecules from the β-turn library. An assay that takes advantage of the autocleavage that the protein undergoes in alkaline conditions was based on previously described methods (Mo et al., 2018; Gonzalez et al., 2019). To establish the optimal pH for the screen, a pH response curve was made with the *E. coli* LexA protein. In each reaction 0.4 μg of LexA was used, which was diluted in 10 mM Tris-HCl, 150mM NaCl (4.5 μL). Cleavage buffer consisting of 50 mM CAPS-NaOH (pH 10.00, pH 10.22, pH 10.42, pH 10.60), 150 mM NaCl was added (4.5 μL) to begin autoproteolysis. After 30 min, reactions were terminated by the appropriate addition of 4X SDS sample buffer and freezing on dry ice.

For the screen, 0.4 μg of LexA was used per reaction, which was diluted in 10 mM Tris-HCl, 150mM NaCl (4 μL/sample). To this, compound suspended in DMSO was added to a final concentration of 20 μM (0.5 μL/5.55%) and incubated for 10 min at 37°C. Cleavage buffer consisting of 50 mM CAPS-NaOH (pH 10.00) was then added (4.5 μL) and the mix incubated for 30 min. The reactions were terminated with 4X SDS sample buffer and frozen on dry ice.

The products of the autocleavage reactions were subjected to electrophoresis in SDS-PAGE gels containing 4-12% polyacrylamide (ThermoFisher Bis-Tris, Bolt™). Proteins were stained with Coomassie brilliant blue using a Bio-Rad Trans-Blot Turbo. The gels were imaged on an Odyssey CLx and quantified with Image Studio.

### 2.7 *In vitro* RecA-mediated LexA cleavage reactions

The RecA-mediated cleavage assay was based upon a previously described procedure for the cleavage of SetR_ICE391_ (Gonzalez et al., 2019) with modifications. Two 50 μL master mixes were made in a buffer consisting of 40 mM Tris-HCl (pH 7.4), 10 mM MgCl_2_, 1mM DTT and 30 mM NaCl. One mix contained 2.5 μg of RecA, 200 ng ΦX174 virion (ssDNA) (New England Biolabs; cat; N3023S) and 1 mM ATPγS. The other mix contained 15 μg of LexA and either DMSO (5% of total) or the compound of interest in DMSO (5% of total). These master mixes were pre-incubated at 37°C for 10 minutes to activate RecA and allow the compound to interact with LexA. Afterwards, the mixes were combined and further incubated at 37°C. 14 μL aliquots were taken at 5-minute intervals. The reaction at each timepoint was terminated by the addition of 4 x SDS sample buffer and freezing on dry ice. After electrophoresis of the timepoint samples in SDS-PAGE gels containing 4-12% polyacrylamide (Invitrogen NuPAGE 4-12% Bis-Tris), the gels were stained with Coomassie brilliant blue. Subsequently, the gels were imaged and quantified using Image StudioLite.

### 2.8 Homology with superfamily members

We conducted a protein sequence alignment of *E. coli* LexA (A0A418GQD6), *B. subtilis* DinR (P31080), *E. coli* UmuD (E7BTC7), *S. typhimurium* UmuD (A0A648F2G5) and SetR_ICE391_ (A0A6G8F0T0) from UniProt using the Bio3D package (Grant et al., 2006) implemented in R version 4.2.2 (R Core Team, 2022). The alignment was visualised using ESPript 3.0, and the *E.coli* LexA structural features with the docked compound **1** was visualised with ENDscript 2.0 (Robert and Gouet, 2014).

## 3 Results

### 3.1 β-turn definition

**Figure 4:**
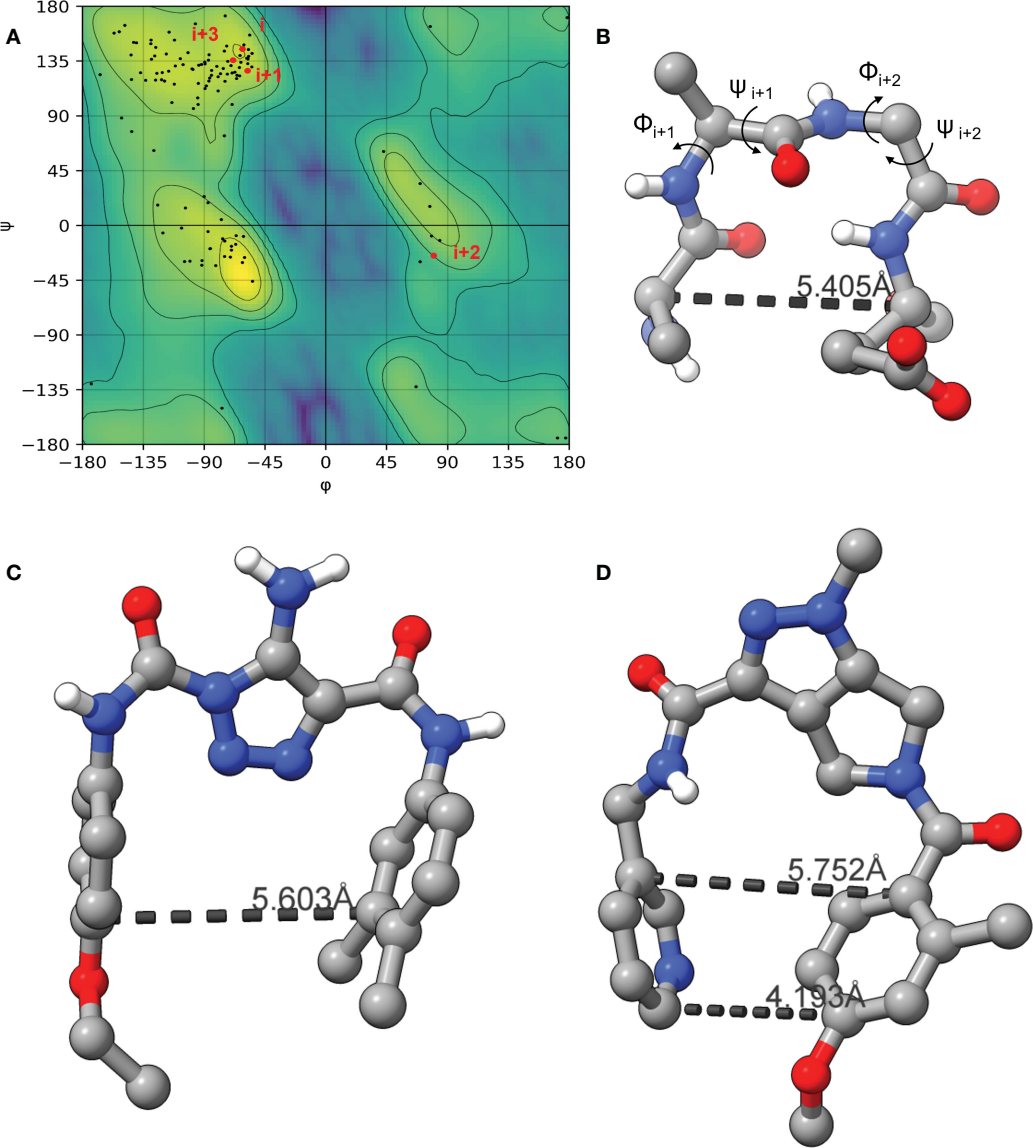
The Ramachandran plot of LexA highlighting the dihedral torsions between β-turn residues of the CSR region are presented in **(A)**. The key atoms in the β-turn of the CSR that contains scissile bond between Ala-84 and Gly-85 are shown in **(B)**. The dihedral torsions of the CSR β-turn are detailed in **Table 1**. These torsions determine the classification of the β-turn. The docked model of the compound GSK-C1 is shown in **(C)**, **(D)** is the only β-turn mimetic compound from our screen that took a conformation reminiscent of the β-turn.

As the LexA CSR is a β-turn, we hypothesized that β-turn peptidomimetics would bind to the same region of the LexA CTD, thereby preventing cleavage from occurring. It was recently predicted (Jaramillo et al., 2022) that the current 5-amino-1-(carbamoylmethyl)-1H-1,2,3-triazole-4-carboxamide scaffold (Selwood et al., 2018) was a β-turn mimetic, as the 1,2,3-triazole ring provides a geometry similar to a β-turn (Mo et al., 2018). Therefore, we were interested in classifying the β-turn of LexA, which would facilitate a visual comparison between the LexA β-turn and the conformation that docked compounds would take. β-turns are classified according to the dihedral torsion angles (Φ and ψ) between the amino acid residues *i+*1 and *i+2* (Venkatachalam, 1968). The standard nomenclature for β-turn types are: I, I’, II, II’, VIII, Vla1, Vla2, Vlb and IV (Hutchinson and Thornton, 1994). As more protein structures are being elucidated, these classifications are being changed, with new turns being defined. This gives definitions to turns that previously were considered “non-standard” but occur with a high frequency. We have analyzed the β-turn in LexA (residues 83-86) to classify it. According to the standard classification, it falls into type II (de Brevern, 2016), however a more recent classification puts it under the SC2-SC10 turn. This is classified according to the dihedral torsions in **Table 1**. In approximately 68% of the turns that fall into this new classifier, glycines occur at the *i*+2 position (Zhang et al., 2022). The distance between the α-carbons of the *i* and *i+3* is 5.405 Å, and the 2.610 Å distance between the *i* oxygen and *i+3* hydrogen indicate a hydrogen bond between the molecules (**Figure 4B**). This is narrower than most standard β-turns, which have distances up to 7 Å (de Brevern, 2016; Ahn et al., 2017).

**Table 1:** Dihedral torsions of the CSR β-turn.

Residue	φ	Ψ
Glu-83 (*i*)	-62.5°	145.65°
Gly-84 (*i+1*)	-58.86°	126.78°
Ala-85 (*i+2*)	79.02°	-24.45°
Ala-86 (*i+3*)	-68.97°	136.73°

These torsions were calculated from PDB 1JHF (Luo et al., 2001) using BIOVIA Discovery Studio (Biovia, 2019). Torsions are between four atoms and are used to classify β-turns.

When the compound GSK-C1 was docked to the hydrophobic cleft, we found it to take on a conformation reminiscent of a β-turn. One of the classifications of a β-turn is that the *i* and *i+3* residues are less than 7.0 Å (Ahn et al., 2017) from each other. In the case of GSK-C1, there is 5.608 Å (**Figure 4C**) between the aromatic rings, occupying a slightly larger space in the pocket than the β-turn does. Based on this modelling we decided to screen a library of β-turn mimetics.

### 3.2 β-turn peptidomimetics screen

A common approach to inhibiting proteases is the use of peptidomimetics (Amblard et al., 2018; Capasso et al., 2021; Bai et al., 2022). Peptidomimetics are slightly modified backbones or sidechains, possibly sharing topological similarities with peptide features. These modifications can be used to hone the properties such as cell permeability, target specificity, and stability. Based on the results of the GSK molecule QSAR assessment and our analysis of the CSR β-turn, we performed a structure-based virtual screen of the 2276 molecules of the ChemDiv β-turn peptidomimetics library (**Table 2**) using the FlexX implementation in SeeSar version 10.1 (BioSolveIT GmBH, 2020). The results of the docking campaign showed that the mimetics occupied the binding site with a similar conformation to GSK-C1, the β-turn mimetic, and some extended down to bind in regions closer to the CSR. The main point of differentiation between the docked compounds was the structural features used to mimic a β-turn. Where there are a variety of β-turn types, denoted by the torsions between residues, these structural features change the turn of a mimetic. Looking at **Table 2**, most of these top compounds contain heterocyclic spiro groups (2, 3, 4, 5, 8, 10). The results also indicate the importance of strong hydrogen donor/acceptor groups in the compounds as they can stabilise the compound within the hydrophobic pocket. The only compound in this library that when docked, took a conformation like that of a β-turn is compared in **Figure 4D**. Molecules were selected based on their score when compared to the benchmark molecules and included an enriched scaffold diversity. We selected the 53 top scoring compounds from the screen to test *in vitro*.

**Table 2:** The top 10 compounds docked to LexA using BioSolveIT, with HYDE rescoring predicted affinities (Schneider et al., 2013).

Ranking	Compound ID	Molecular Weight	Predicted Affinity [μM]	Structure
1	CM1461-0224	397.467	18.48	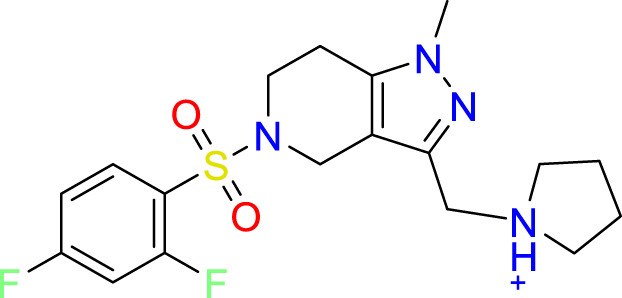
2	T787-5165	398.544	26.51	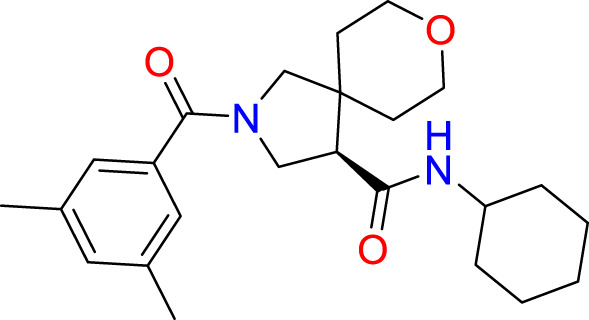
3	S635-3152	355.317	83.81	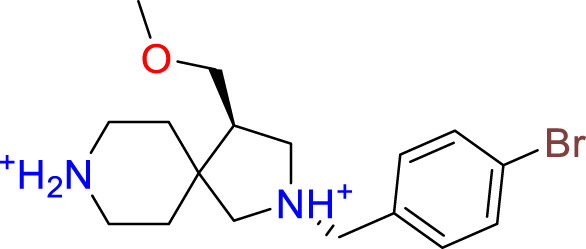
4	S635-2320	357.515	97.81	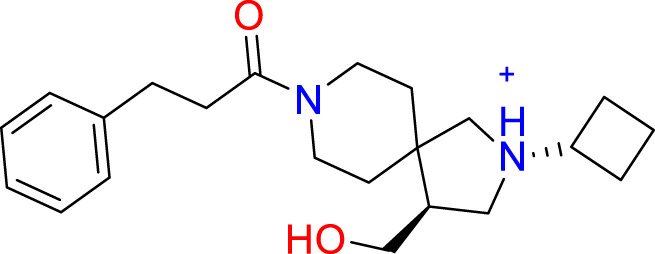
5	S635-4217	398.495	98.84	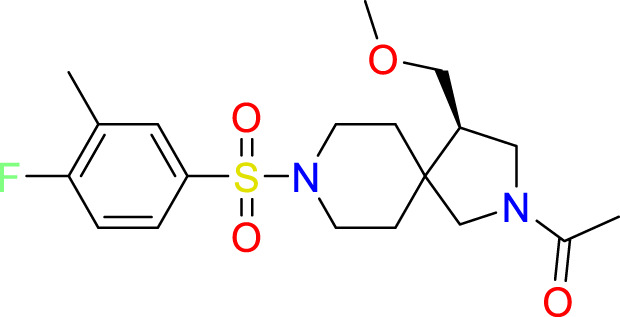
6	L871-0125	522.6	107.81	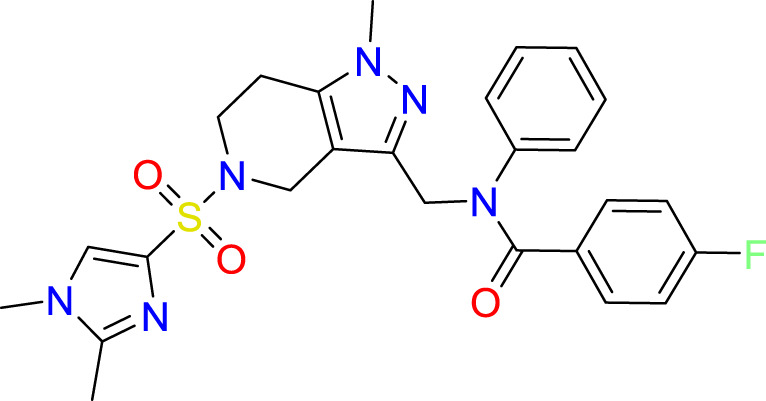
7	CM1461-0349	430.377	118.36	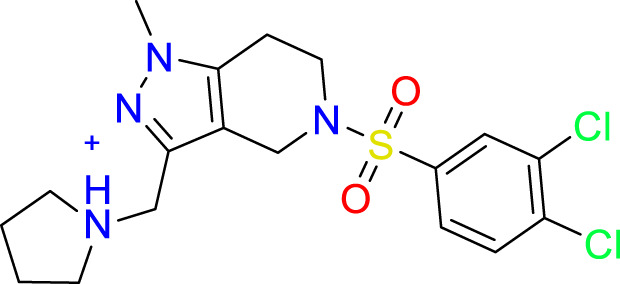
8	T655-0553	397.516	122.96	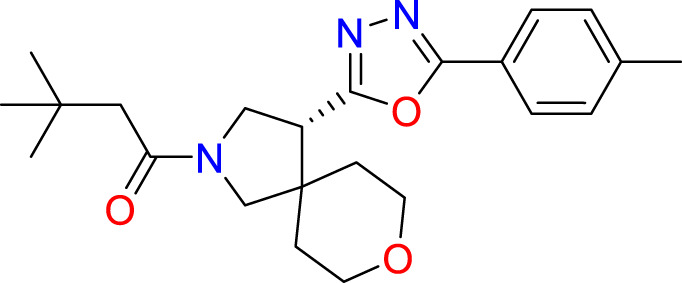
9	S398-2187	405.456	130.07	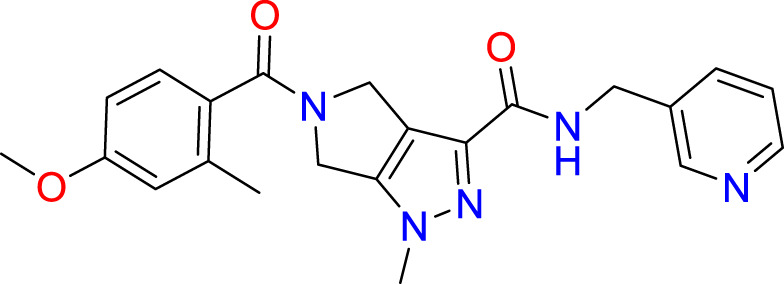
10	S635-2899	368.293	157.31	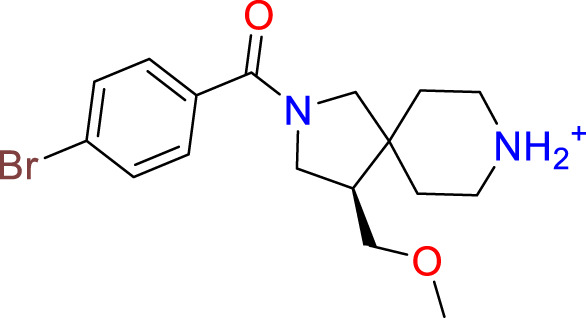

### 3.3 Covalent warhead screen

We performed a structure-based covalent screening protocol after it was shown that it was possible to prevent the cleavage of LexA by targeting the catalytic Ser-119 with covalent warheads (Bellio et al., 2020). Using Schrödinger’s CovDock feature, the ChemDiv Generic and Smart covalent inhibitor libraries were docked. This revealed scaffolds that demonstrated improved binding scores over the boronic acids tested by Bellio et al. (Bellio et al., 2020). The top ten scoring compounds using the OPLS3e forcefield are in **Table 3**. These are predicted to bind by a series of different reactions, dependent on the functional group chemistries available.

**Table 3:** Top-ranked serine covalent warheads from structure-based virtual screening against LexA, predicted with Schrödinger Covdock.

Compound ID	Molecular Weight	Structure	Docking score	Reaction
T002-1796	270.33	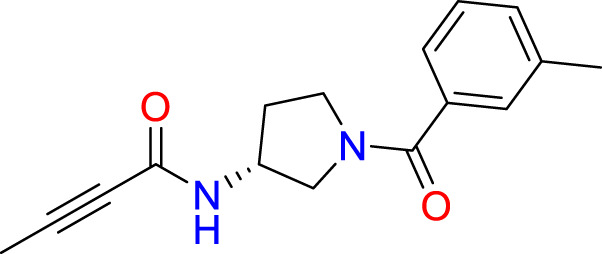	-6.22	Conjugate Addition to Alkyne (carbonyl activated)
T002-1859	274.30	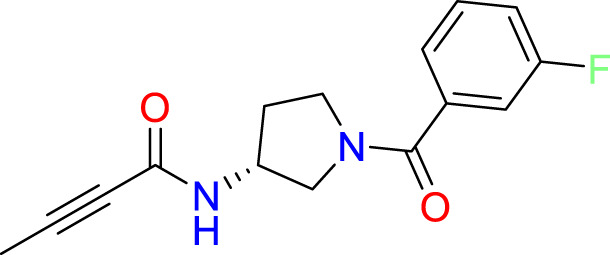	-6.13	Conjugate Addition to Alkyne (carbonyl activated)
T002-1864	284.36	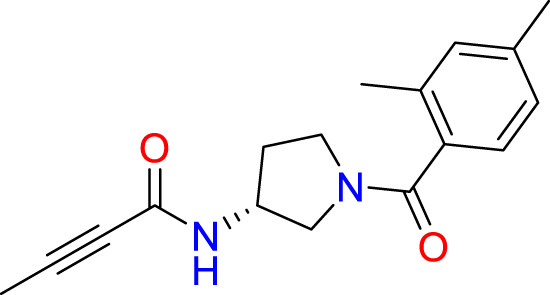	-5.98	Conjugate Addition to Alkyne (carbonyl activated)
M074-0516	244.30	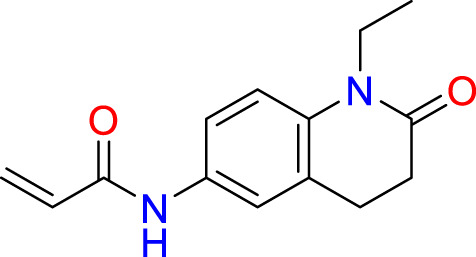	-5.97	Nucleophilic Addition to a Double Bond
ZE09-1281	282.30	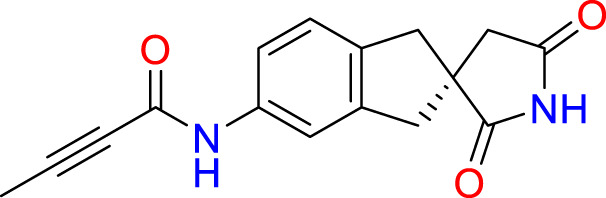	-5.87	Conjugate Addition to Alkyne (carbonyl activated)
S644-0079	414.46	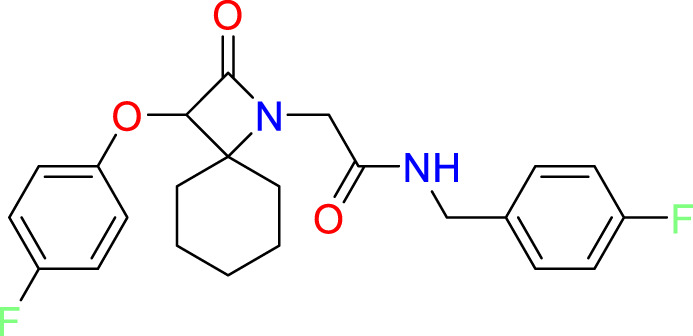	-5.78	Beta Lactam Addition
T002-1895	257.29	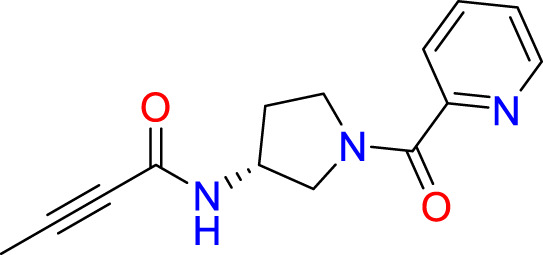	-5.71	Conjugate Addition to Alkyne (carbonyl activated)
0682-0046	252.27	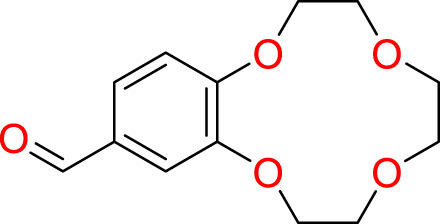	-5.64	Nucleophilic Addition to a Double Bond
S642-0048	380.47	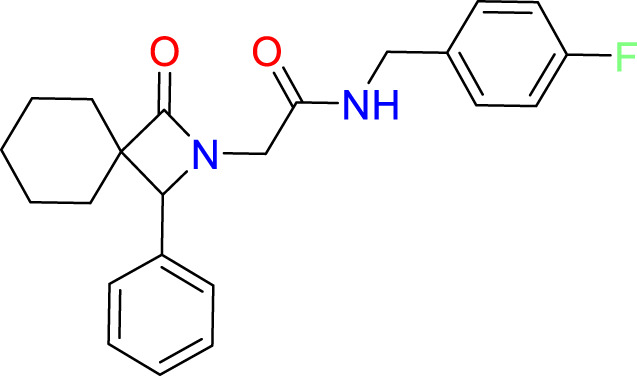	-5.64	Beta Lactam Addition
T002-2908	323.40	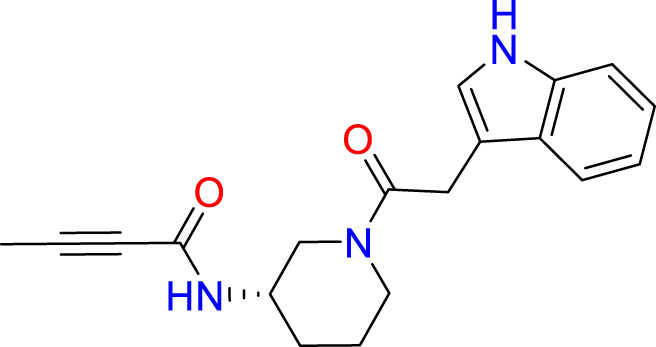	-5.64	Nucleophilic Addition to a Double Bond

### 3.4 *In vitro* screen

**Figure 5:**
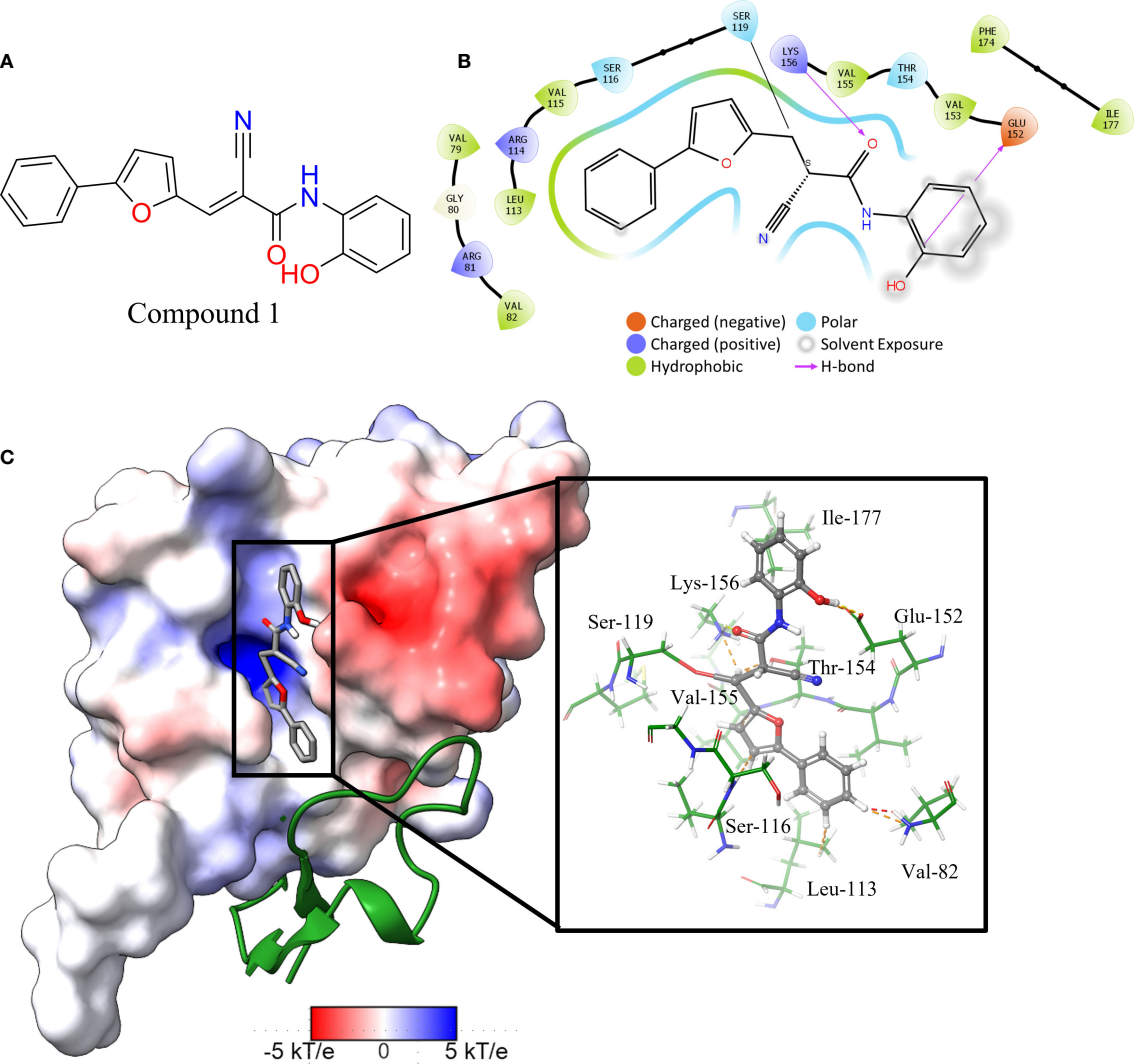
Interaction profile of compound 1. **(A)** 2D structure of compound 1. **(B)** 2D representation of the amino acid residues directly interacting with the compound. Residue color is based on the type, grey atom background is the solvent-accessible surface area. **(C)** The full LexA CTD with the bound compound and the same electrostatic surface as in **Figure 3**, with the DelPhi Webserver generated electrostatic surface. The ribbon also denotes the CSR of LexA. The insert shows the 3-D orientation of the molecule with the surrounding interacting residues.

The *in vitro* screen carried out was based on the LexA’s ability to undergo autoproteolysis in alkaline conditions. Our initial development of the assay determined the optimal conditions for the screen to be pH 10.00 for 30 min. Using this property, we screened the 133 compounds for inhibition activity. Of these, 12 compound exhibited some inhibitory effect under autocleavage conditions (**Table S1**), however once tested in the RecA-mediated counter assay, only one compound exhibited an inhibitory effect on LexA proteolysis. Compound **1** (ChemDiv ID: 2381-1036, **Figure 5A**) covalently bound to the catalytic Ser-119, thereby inhibiting LexA cleavage. This compound reacts with the serine *via* a nitrile-activated conjugate addition, where the alkene reacts to bind to the serine. In docking this compound scored -4.841, worse than the top scoring compounds.

**Figure 6:**
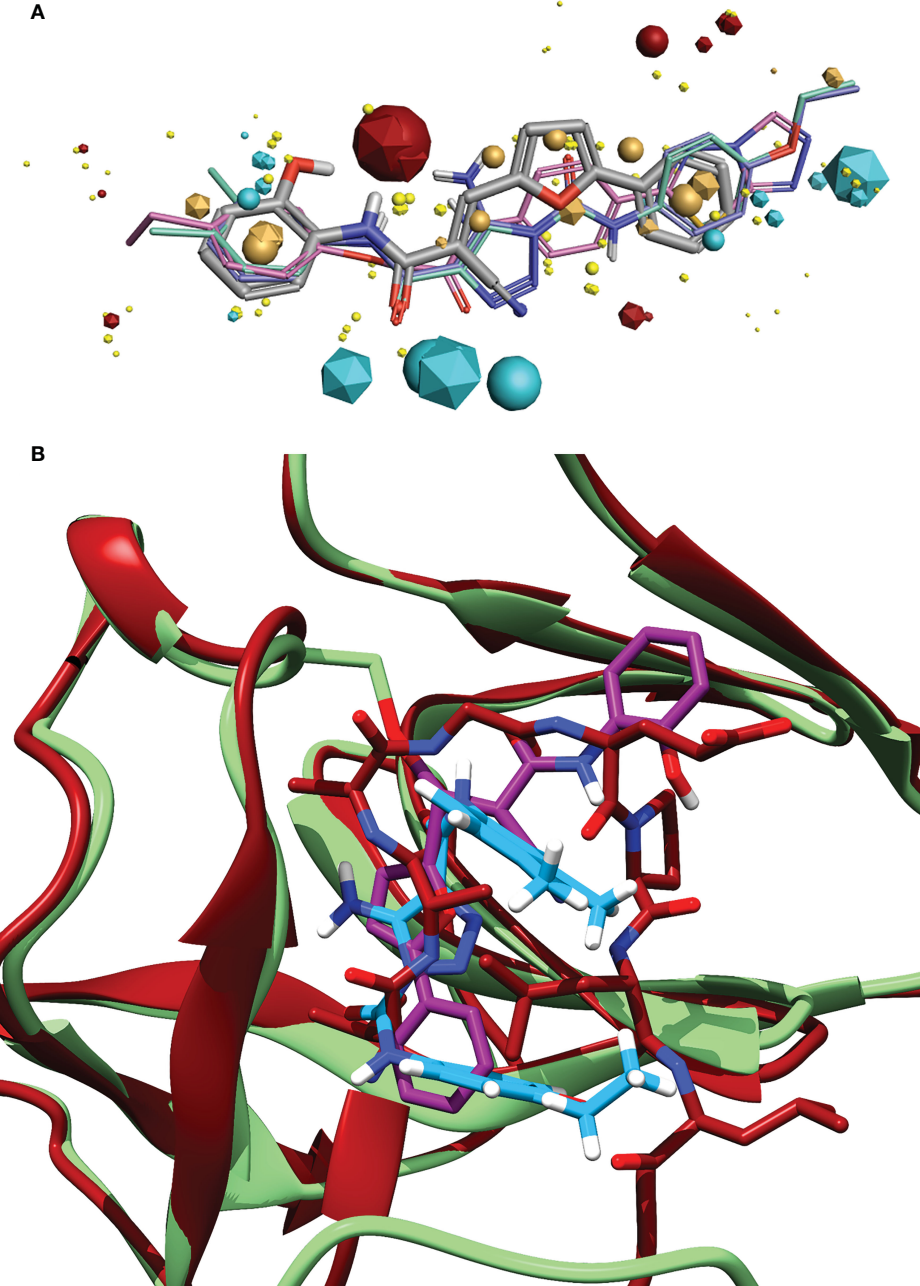
**(A)** Alignment of the identified compound to three previously identified inhibitors, showing the similarity of features between the ligands. The key features are represented by the spheres (Compound 1) and icosahedrons (previous inhibitors). Compound 1 is denoted by the grey carbons, and thick bonds. The previous inhibitors GSK-D1 (pink), GSK-C1 (lilac) and the lead from Selwood et al. (Selwood et al., 2018) are represented by thin lines. Red features are electropositive regions, cyan are electronegative, tan are hydrophobicity fields and yellow van der Waals fields. The size of the field corresponds to the size of the effect. Image generated using Flare ™ from Cresset^®^. **(B)** An alignment of the C-form of LexA (red) with GSK-C1 (blue) and compound 1 (purple) bound to the NC-form of LexA (green). This shows the similarity of these compound conformations to the β-turn of the C-form.

Analysis of the top scoring docked pose of this compound shows that there are three main ways that compound **1** interacts with the LexA hydrophobic pocket (**Figure 5**). The primary interaction is the covalent bond with the oxygen of the catalytic Ser-119. The second essential interaction is the hydrogen bond between the donating Lys-156 and the hydrogen bond accepting oxygen of the acetamide. Thirdly, the terminal aromatic hydroxyl group on compound **1** acts as a hydrogen bond donor to the glutamic acid carboxyl group. This compound takes a conformation that is in line with the LexA CSR when the protease is in the C-form (**Figure 6B**). The hit rate of the virtual screen was 1.22%, the hit rate of the *in vitro* screen was 0.75% calculated according to the methods outlined by Zhu et al. (Zhu et al., 2013). In cases such as this when a novel scaffold is identified, a low hit rate is considered preferable to a high one (Sotriffer, 2011).

We did not find any β-turn mimetics from the ChemDiv β-turn mimetic library that inhibited the RecA-mediated cleavage of LexA. While some demonstration mild inhibition of LexA autoproteolysis under alkaline conditions, when tested with RecA they did not exhibit the same inhibitory effect. An analysis of the β-turn mimetics conformations reveals that most of them do not take the same tight hairpin turn that GSK-C1 or the CSR β-turn does (**Figures 4**, **S2**). In the top 10 scoring β-turn mimetics from the virtual screen, we only see one that takes a hairpin conformation (**Figuresnbsp;4D**, **S2I**) close to that of the CSR β-turn or the conformation that GSK-C1 takes (**Figure 4C**). The compound ranked at 9 in **Table 2** has a distance of 4.193 Å between the terminal carbons of the pyridine and aromatic rings at each end, and 5,752 Å between the carbons at the para positions. This is close to the 5.405 Å between the β-turn *i* and *i+3* α carbons, and the 5.603 Å at the narrowest point of GSK-C1. This compound bound in the reverse position, as the central methylated double heterocycle would have larger steric clashes than those of GSK-C1. GSK-C1 binds in the opposite direction in a manner closer to the CSR β-turn (**Figure S2K**).

### 3.5 Pharmacophore of top hit

Exploring the pharmacophore of compound **1** reveals its similarities to previous hit compounds. Using Cresset’s FieldTemplater software in Flare v6.0 (Cheeseright et al., 2006; Kuhn et al., 2020), we developed a pharmacophore of these active compounds. In **Figure 6A**, the major electropositive and electronegative fields of the inhibitors overlap or are close in position. The same can be observed in the overlapping aromatic rings. Despite the new compound being a covalent inhibitor, it was revealed to have a similarity of 0.768 to the other three compounds, combining the field (electrostatic and hydrostatic) similarity and the shape similarity of the molecules. The similarity to previous inhibitors of LexA cleavage further supports the non-covalent interactions that compound **1** has with the catalytic site of LexA.

### 3.6 *In vitro* inhibitor cleavage profile

**Figure 7:**
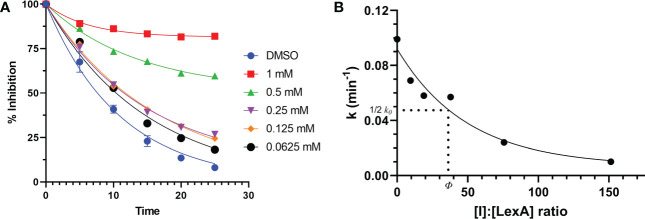
Time course of RecA-mediated LexA proteolysis in the presence of different concentrations of compound 1 **(A)**. Example gels are in Supplementary Material **Figure S4**. This was used to derive values of *k* for each concentration. The second graph **(B)** plots the values of *k* derived from graph **(A)** as a function of the [I]:[LexA] ratio. The value of ϕ is the ratio that is half of the rate constant *k_0_*.


To understand how compound **1** inhibits LexA cleavage over time, a series of RecA-mediated cleavage reactions were conducted. Between 0 and 25 minutes, compound **1** demonstrated inhibition at a series of concentrations (**Figure 7A**, gels in **Figure S3**). Testing between 1 mM and 0.0625 mM concentrations, compound **1** demonstrated inhibitory activity at all these concentrations, with significantly higher activity at 0.5 mM and 1 mM of inhibitor. To compare the inhibition of compound **1** with the other covalent inhibitors of LexA cleavage we used the kinetic model described by Bellio et al. (Bellio et al., 2020) to determine dissociation constant K_d_. LexA proteolysis behaves in a non-Michaelian manner as it is unimolecular, and the protein is consumed during the reaction. As RecA theoretically binds in a 1:1 ratio, as does the inhibitor, we assumed that the proteolysis reaction could be described as:

$$\begin{array}{c} LexA\overset{k}{\to }NTD+CTD\;\left(1\right) \end{array}$$

We assume that [LexA] is the concentration at any time during the reaction and [LexA]_0_ is the initial concentration of LexA. In the case of our experiments, these values were the band intensities, and the ratio was between the initial aliquot and then each subsequent aliquot. As the cleavage occurs as an exponential decay, the integrated rate law that applies to this equation is:


$$\begin{array}{c} {\frac{\left[LexA\right]}{\left[LexA\right]}}_{0}=e^{-kt}\;\left(2\right) \end{array}$$

where *k* is the first order rate constant, and *t* is the time. We fit this equation to the LexA cleavage without inhibitor, to obtain the rate constant *k_0_* (**Figure 7**). By calculating the *k_i_* for each molar ratio ([LexA]/[LexA]_0_) the effectiveness of these inhibitors can then be described with the equation:


$$\begin{array}{c} k_{i}=\frac{k_{0}}{1+\frac{\left[I\right]:\left[LexA\right]}{\text{Φ}}}\;\left(3\right) \end{array}$$

where [I] is the inhibitor concentration and ϕ is the effectiveness at half of the first order rate constant (*k_0_)*. Plotting the *k_i_* values against the molar ratios, it is possible to determine how effective the inhibitor is at inhibiting LexA cleavage (**Figures 7**, **7B**). In the case of compound **1**, the effectiveness was determined to be 43.422, which equates to a K_d_ of 286.36 μM – significantly lower than those of the boronic acid compounds published by Bellio et al. (Bellio et al., 2020).

### 3.7 Comparison of compound 1 binding pocket and related proteins

An analysis of the amino acids interacting with compound **1** from the docking reveals the residues that stabilise the compound in the binding pocket (**Figure 5**), we see the Lys-156 forming hydrogen bond with the oxygen of the acetamide group. The interaction diagram further depicts the residues that stabilise compound **1** in the pocket (**Figure 5B**) and the orientation of the compound in the hydrophobic pocket (**Figure 5C**).

While we determined that compound **1** bound to LexA *in vitro*, it was of interest to investigate if it potentially would bind to structurally similar proteins in other species. We investigated *B. subtilis* DinR, *E. coli* UmuD, *S. typhimurium* UmuD, and SetR_ICE391_ as they share structural homology with LexA. A multiple sequence alignment showed that not only are Ala-84, Glu-85, Ser-119, and Lys-156 conserved (**Figure S5**), but other residues in the catalytic cleft are as well, an expected occurrence due to the retention of function by these proteins (Burckhardt et al., 1988; Haijema et al., 1996; Gonzalez et al., 2019). Using ENDScript 2.0 (Robert and Gouet, 2014), residues within 3.2 Å and 3.2-5 Å of the compound were evaluated (**Figure S5**). Several of these residues, particularly Glu-152 – Lys-156 are mildly conserved across species.

## 4 Discussion

The goal of this study was to continue the search for novel inhibitors of the SOS response in *E. coli* by inhibiting the transcriptional repressor LexA. We approached this through a targeted campaign combining *in silico* and *in vitro* techniques. In this approach, we hypothesized a binary approach searching β-turn peptidomimetics and covalent compound libraries for a molecule that binds to the hydrophobic cleft of LexA and prevent the proteolysis of the protein. The cleavage of this dimer is regulated by the Ser-119 in the active site, which in the NC conformation is in a catalytic dyad with Lys-156. The formation of the transient tetrahedral with Ala-84 and Gly-85 and subsequent hydrolysis of the amide bond regulates the cleavage rate.

Our workflow resulted in the discovery of one compound - (2E)-2-cyano-N-(2-hydroxyphenyl)-3-(5-phenyl-2-furyl)acrylamide - that exhibited inhibitory activity against LexA. This compound, while not as effective as the non-covalent inhibitor that has been published by Selwood et al. (Selwood et al., 2018), further demonstrates the possibility of using covalent inhibitors against RecA-mediated LexA cleavage. The resulting compound shares some similarities, such as the aromatic end-groups and the presence of acetamides in the chains. Using SwissADME (Daina et al., 2017), the pharmacokinetic properties of compound **1** were estimated. Based on the predicted WLOGP and TPSA, the molecule would be absorbed into the GI tract (Daina and Zoete, 2016). Optimization of the compound would seek to improve the pharmacokinetic profile and the inhibitory activity.

We reasoned that the previous 1,2,3-triazole inhibitors (Mo et al., 2018; Selwood et al., 2018) acted as β-turn mimetics. This was supported by the recent paper by Jaramillo et al. (Jaramillo et al., 2022). When comparing the docked structure of GSK-C1 to the CSR turn we determined that it appeared to take a β-turn-like conformation, but in a different orientation to that of the CSR, instead interacting with residues on the CSR (**Figure 6**). While the current lead inhibitors mimic β-turns, our *in silico* screen did not result in any β-turn mimetics that were active in RecA-mediated LexA proteolysis. The limited library selection may have caused this, as the mimetics copied wider β-turns than the LexA CSR; a greater range of protein mimetics and an increased scaffold diversity may have resulted in an improved outcome.

Covalent docking techniques have only recently emerged. Molecular modelling programs are still in the process of implementing tools that facilitate this type of docking. Subsequently, the scoring functions of these programs are still being optimized, and each program takes a different approach to how the docking is done. Instances such as ours are not uncommon - where the lead compounds after a covalent screening campaign are not necessarily the compounds that scored highest in the virtual screen (Shen et al., 2022).

Covalent inhibitors require two main features to be effective. The first is the need for a reactive warhead on the molecule that reacts with the catalytic residue. In the case of compound **1**, there are two electrophilic groups – the alkene and the nitrile. CovDock predicted that the alkene group in the middle of the compound, activated by the nitrile group, is how the compound binds to the catalytic Ser-119. While this is the most likely mechanism, the nitrile group on the compound might also be directly reacting with the serine. The second key feature are the functional groups that denote the non-covalent interactions with the binding pocket. These interactions both position the compound to facilitate the covalent binding, and they also stabilize the molecule in the binding pocket after the fact, slowing or preventing a reversible reaction. Based on our docking, the binding of compound **1** to the LexA pocket is stabilized by the Val-82, Leu-113, Ser-116, Glu-152, Thr-154, Lys-156 cand Ile-177 (**Figure 5B**), leaving space to develop the molecule to target interactions with other residues in the hydrophobic pocket. A larger scaffold would theoretically interact more strongly with residues in the binding pocket. When compared to the prior best covalent inhibitor with a *K_d_* of 1.09 mM (Bellio et al., 2020), this compound has one 238.36 μM. Molecular modelling shows that the orientation of compound **1** in the catalytic pocket lines up with *i* and *i+2* residues of the CSR in the activated C-form (**Figure 6**).

Other members of the LexA superfamily have a low sequence homology (**Figure S5**). Despite this, they have a highly conserved structure, and serve closely related roles in different species. The residues interacting with compound **1** (**Figures 5**, **S6**) are conserved across several of the other proteins of the superfamily which suggests that the molecule may have a similar antagonistic effect. Testing these compounds with proteins in the LexA superfamily to determine if it could be applied as a broad-spectrum treatment would be another step in the further development of this compound but was outside the scope of this investigation.

Future development of compound **1** needs to address the solubility in aqueous solutions. Presently, it has a relatively poor solubility in water, with precipitate observable in 0.5 mM concentrations. Rational modification of the molecule would require improving interactions with the other amino-acid residues in the binding pocket to increase target specificity and affinity, improve the bioavailability of the compound, and improve the aqueous solubility. Importantly, it would need to be determined whether compound **1** is able to permeate the bacterial cell wall, and if not, then what modifications would facilitate such movements. To achieve this, *in vivo* assays testing the compound with a bacterial model is necessary.

The major limitation of this investigation is the lack of studies confirming and improving our understanding of the compound activity; extensive mass spectrometry to show the covalent bond formation, cell-based SOS reporter assays (Selwood et al., 2018) or filamentation assays (Bellio et al., 2020). While these would further confirm binding is occurring it would be more appropriate to deal with the solubility issue and improve the binding first. Despite these caveats, we can make a reasonable assumption that the covalent reaction is indeed occurring due to the catalytic dyad within the catalytic pocket. Ser-119 is the most reactive serine on the protein, as the β-hydroxy group of the serine is polarized due to the presence of the Lys-156 thereby increasing the nucleophilicity of the residue (Bellio et al., 2020).

Where previous papers on LexA kinetics have investigated the kinetic relationship between RecA concentrations and LexA cleavage rates, only one study (Bellio et al., 2020) has defined an approach to kinetically studying the effectiveness of inhibitors against LexA. We were able to adapt this to our approach when defining how effective a compound is at inhibiting LexA cleavage. Moving forward, this robust method is well suited to quantitatively comparing inhibitor effectiveness.

## 5 Conclusion

This work is the first binary approach to finding novel LexA inhibitors – investigating both β-turn mimetics and covalent warheads. The combined *in silico* and *in vitro* workflow based on rational library selection eliminated the need to physically test millions of compounds to identify novel scaffolds against the LexA transcriptional repressor. In studying the β-turn of the CSR, we determined that β-turn mimetics need to take conformations close to that of the protein’s native β-turn, with limited bulky functional groups so that the compound can form a narrow turn. This is why none of the β-turns in the selected library inhibited the RecA-mediated cleavage of LexA. A previously unidentified covalent scaffold that inhibits RecA-mediated LexA cleavage was identified. This scaffold binds the catalytic Ser-119 *via* a different mechanism compared to the boronic acids published by Bellio et al. (Bellio et al., 2020) and does so with a stronger effect. Further optimization of this scaffold is required, to improve solubility and increase non-covalent interactions within the binding pocket. While molecular modelling indicates that the novel compound forms a covalent bond with the catalytic Ser-119, experimental confirmation of this reaction would strengthen these results. Assays that determine the potency of this compound on *E. coli* strains would determine if this compound antagonizes the bacterial SOS response.

## Data availability statement

The original contributions presented in the study are included in the article/**Supplementary Material**. Further inquiries can be directed to the corresponding authors.

## Author contributions

ZS, NG, RW, DR, and LC contributed to conceptualisation of the study and discussion of implications. ZS, NG conceptualised and carried out *in silico* experiments. ZS, RW, DR contributed to *in vitro* methodologies and performing the analysis. ZS and JM performed *in vitro* assays. ZS performed data analysis and prepared the manuscript. RW, JM, DR and NG provided advice and editing of the manuscript and analysis. All authors contributed to the article and approved the submitted version.
