# Supplementary material for: Integration of molecular modelling and in vitro studies to inhibit LexA proteolysis
Source: Front Cell Infect Microbiol. 2023 Mar 3;13:1051602. doi: 10.3389/fcimb.2023.1051602 (PMC10020695; doi:10.3389/fcimb.2023.1051602)
Supplement: Supplementary file 1 [file Table_1.docx]

Supplementary Material

# Supplementary Data

Figure S1: The compounds from Mo *et al.* (Mo et al., 2018) that were determined to bind to LexA in their paper and the comparison of their docked score to the experimental ln(IC50) from the paper. As there is only a sample size of 3, this is not a significant statistic.


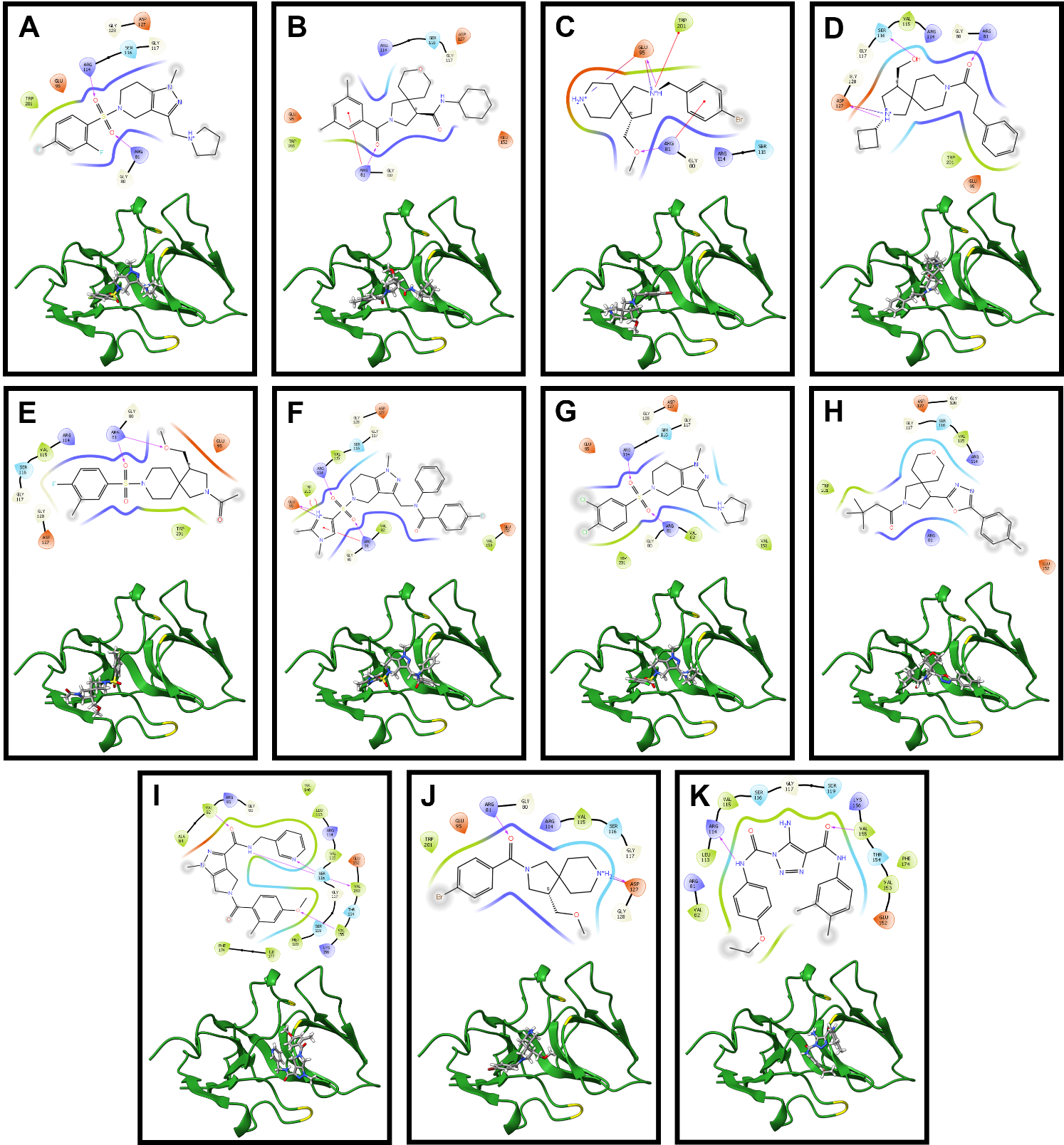
Figure S2: The interaction profile and top docking pose for each of the top scoring beta-turn mimetics (A-J) and the compound GSK-C1 (K) from the Mo *et al.* (Mo et al., 2018) paper. In the docking pose, the LexA CTD is represented by the green ribbon, and the key residues Ala-84, Glu-85, Ser-119 and Lys-156 are highlighted in yellow.

Table S1: The top 12 scoring compounds from the autocleavage LexA screen. The rows highlighted in orange are compounds from the β-turn mimetic library, and rows in white are compounds from the covalent inhibitor library. The row in green is the only compound to also demonstrate inhibition of RecA-mediated cleavage. Note that T787-3223 and S635-2899 are no longer indexed by ChemDiv.

| **Molecule** | **650 μM Inhibition %** | **20 μM Inhibition %** | **1 mM RecA Inhibition %** | **ChemDiv Code** | **Molecular Weight** |
| --- | --- | --- | --- | --- | --- |
| P2-G01 | 52.73 | 19.27 | 2.22 | L871-0125 | 522.61 |
| P2-H01 | 46.83 | 19.97 | -23.13 | L871-0162 | 532.64 |
| P1-D02 | 23.28 | 15.20 | 81.93 | 2381-1036 | 330.35 |
| P1-G03 | 20.32 | 14.49 | 7.284 | S630-0461 | 401.49 |
| P2-C03 | 17.28 | -3.58 | -34.63 | CM2345-1581 | 364.51 |
| P1-A02 | 14.74 | 14.00 | -1.92 | 1487-1661 | 420.47 |
| P1-F08 | 12.82 | 15.62 | -37.55 | T002-1466 | 327.34 |
| P2-C06 | 12.38 | 13.15 | -23.33 | T787-3223 | 257.29 |
| P1-E02 | 12.07 | 13.94 | -21.28 | 2514-2295 | 433.39 |
| P2-D03 | 9.41 | -3.40 | 4.57 | S635-2899 | 403.75 |
| P2-H05 | 2.52 | 14.33 | 3.72 | T655-0422 | 409.45 |
| P2-B06 | -4.10 | 20.99 | 5.45 | T787-2220 | 365.86 |


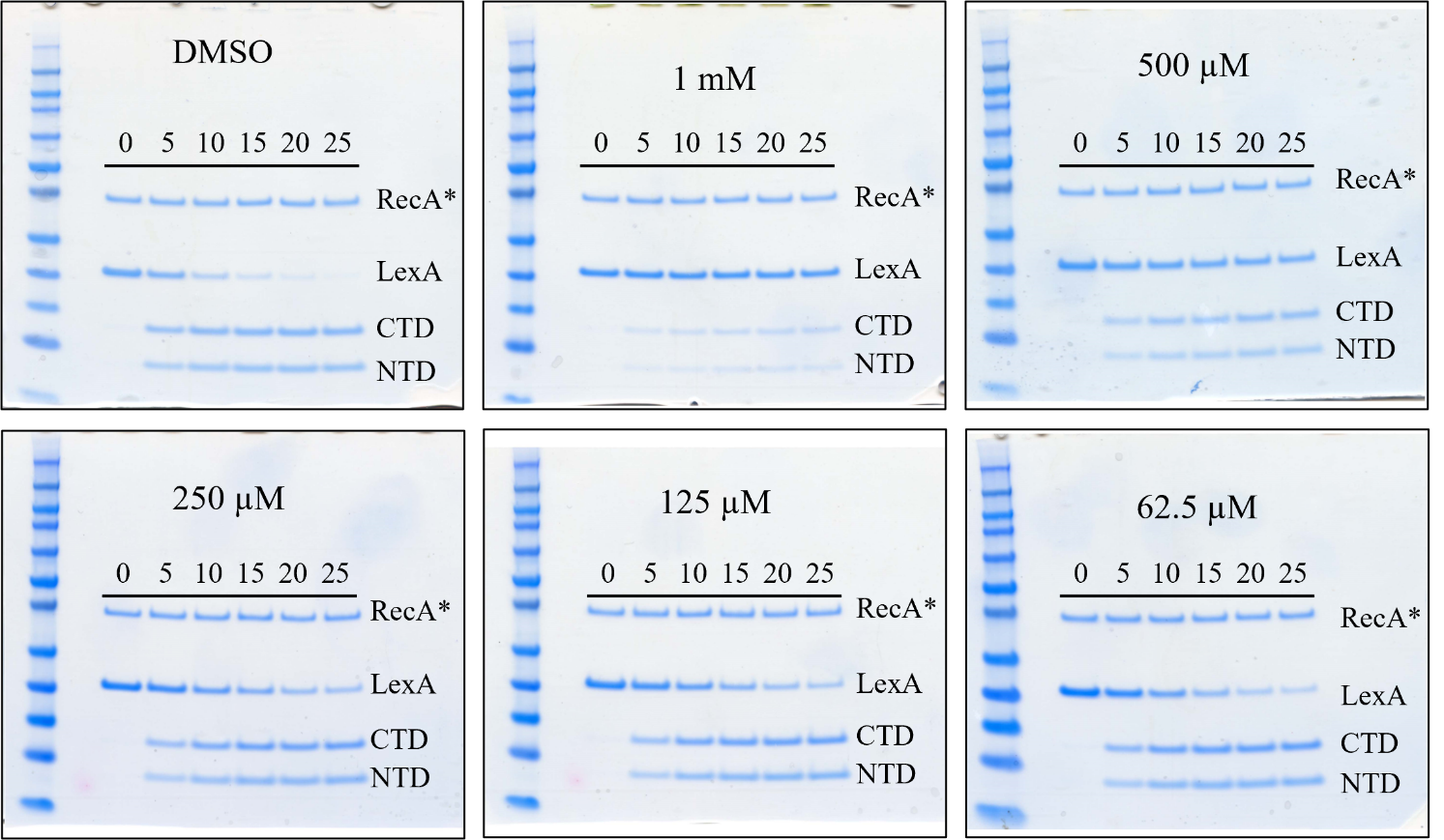


Figure S3: Representative gels of the RecA-mediated cleavage with the different concentrations of the inhibitor 2382-1036. Note that the time scale is in minutes, from 0-25 min.


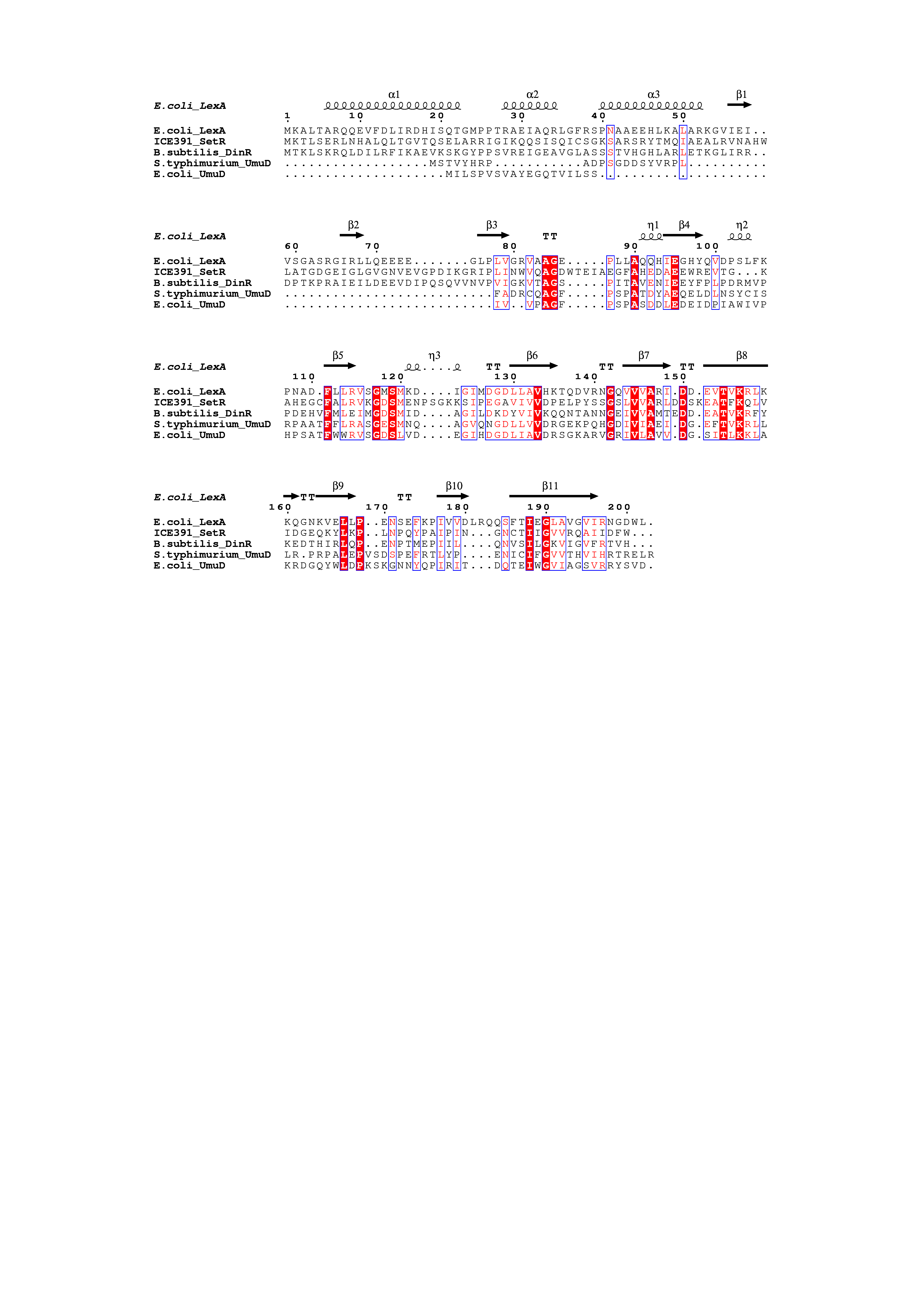
Figure S5: The sequence alignment of the LexA family of proteases, including the shared structural features. Conserved residues are shaded in red blocks, and red letters indicate semi-conserved.


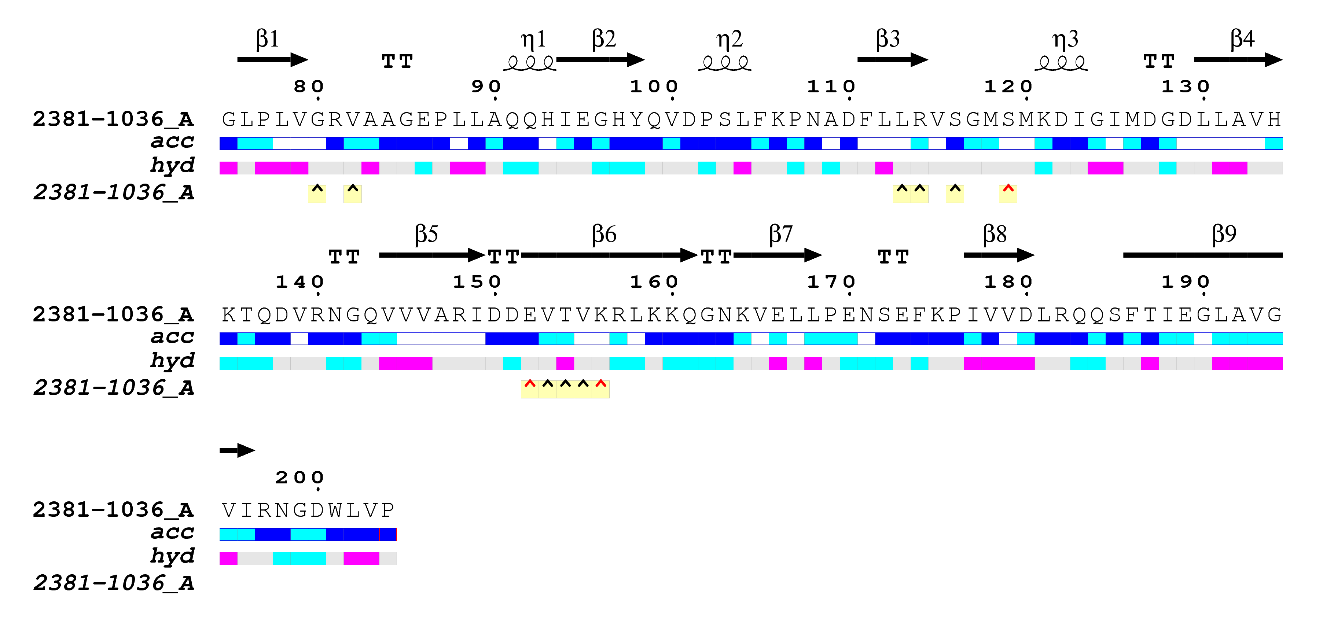


Figure S6: The residues interacting with the bound compound 1, calculated using ENDscript (Robert and Gouet, 2014). The red carrots on the bottom row indicate residues < 3.2 Å away from the compound, and black carrots indicate residues between 3.2 – 5 Å from compound 1.
