# Supplementary material for: Integration of molecular modelling and in vitro studies to inhibit LexA proteolysis
Source: Front Cell Infect Microbiol. 2023 Mar 3;13:1051602. doi: 10.3389/fcimb.2023.1051602 (PMC10020695; doi:10.3389/fcimb.2023.1051602)
Supplement: Supplementary file 2 [file Presentation_1.pptx]

## Slide 1
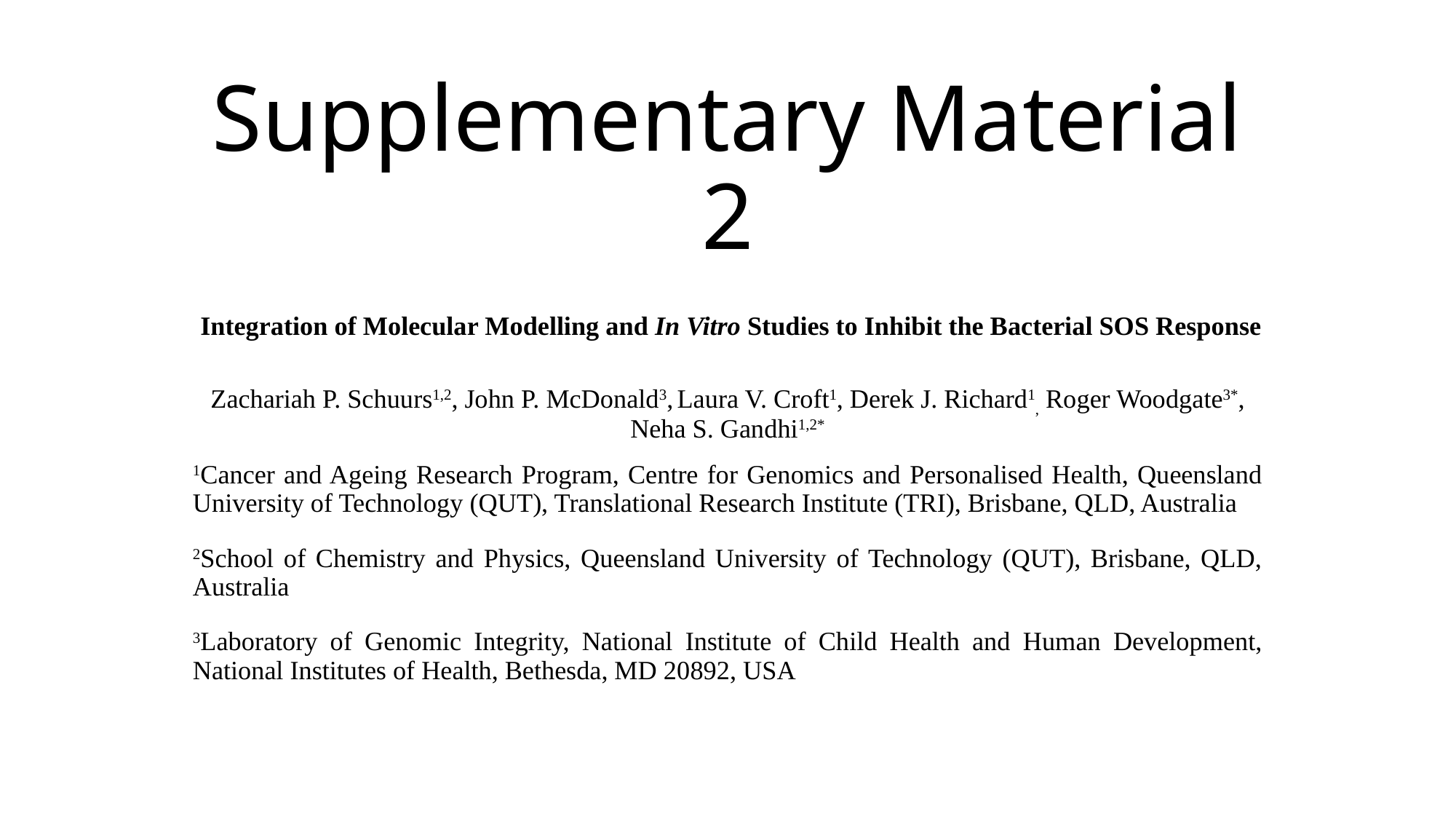

# Supplementary Material 2
 Integration of Molecular Modelling and In Vitro Studies to Inhibit the Bacterial SOS Response
Zachariah P. Schuurs1,2, John P. McDonald3, Laura V. Croft1, Derek J. Richard1, Roger Woodgate3*, Neha S. Gandhi1,2*
1Cancer and Ageing Research Program, Centre for Genomics and Personalised Health, Queensland University of Technology (QUT), Translational Research Institute (TRI), Brisbane, QLD, Australia
2School of Chemistry and Physics, Queensland University of Technology (QUT), Brisbane, QLD, Australia
3Laboratory of Genomic Integrity, National Institute of Child Health and Human Development, National Institutes of Health, Bethesda, MD 20892, USA

## Slide 2
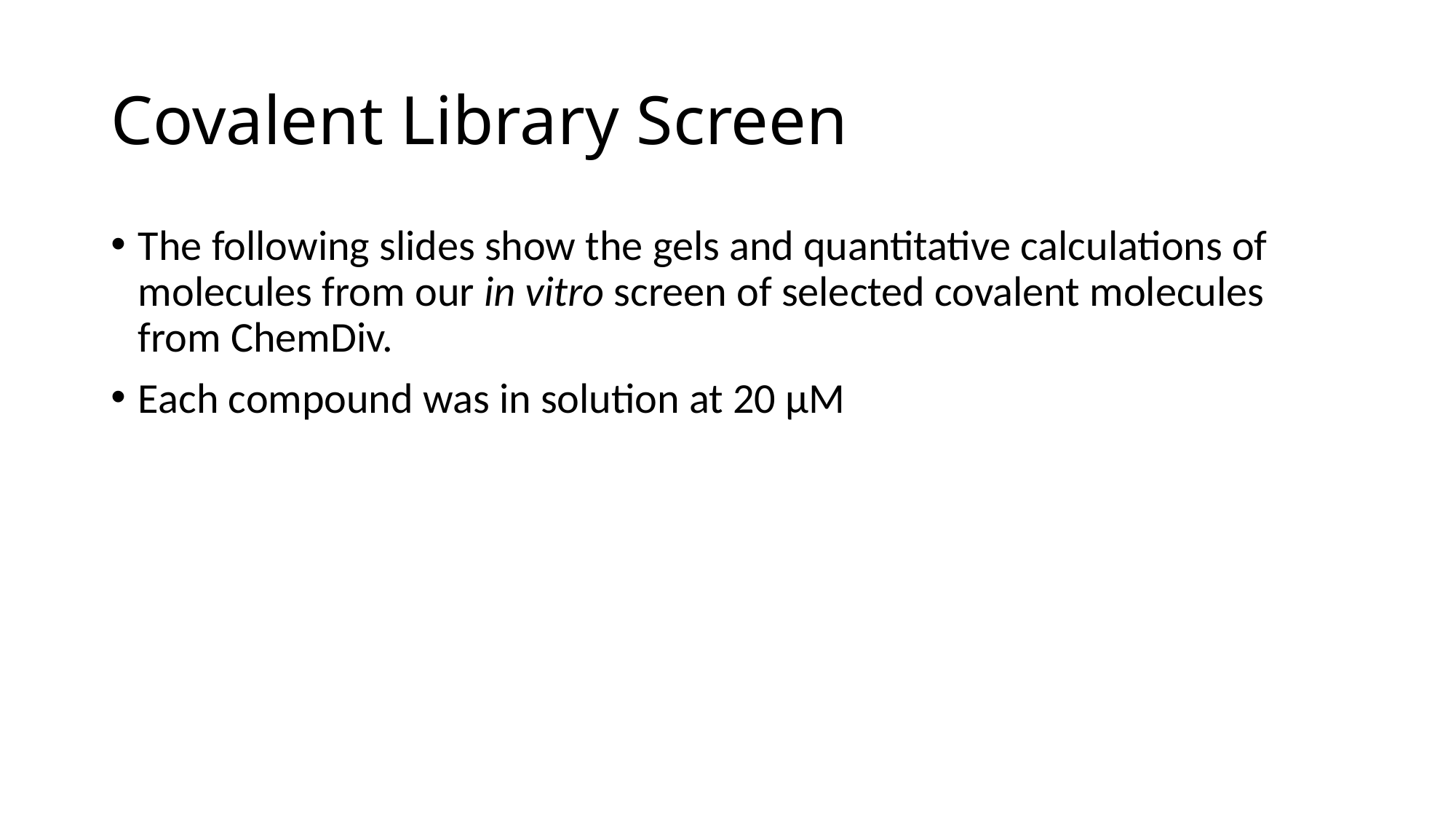

# Covalent Library Screen
The following slides show the gels and quantitative calculations of molecules from our in vitro screen of selected covalent molecules from ChemDiv.
Each compound was in solution at 20 μM

## Slide 3
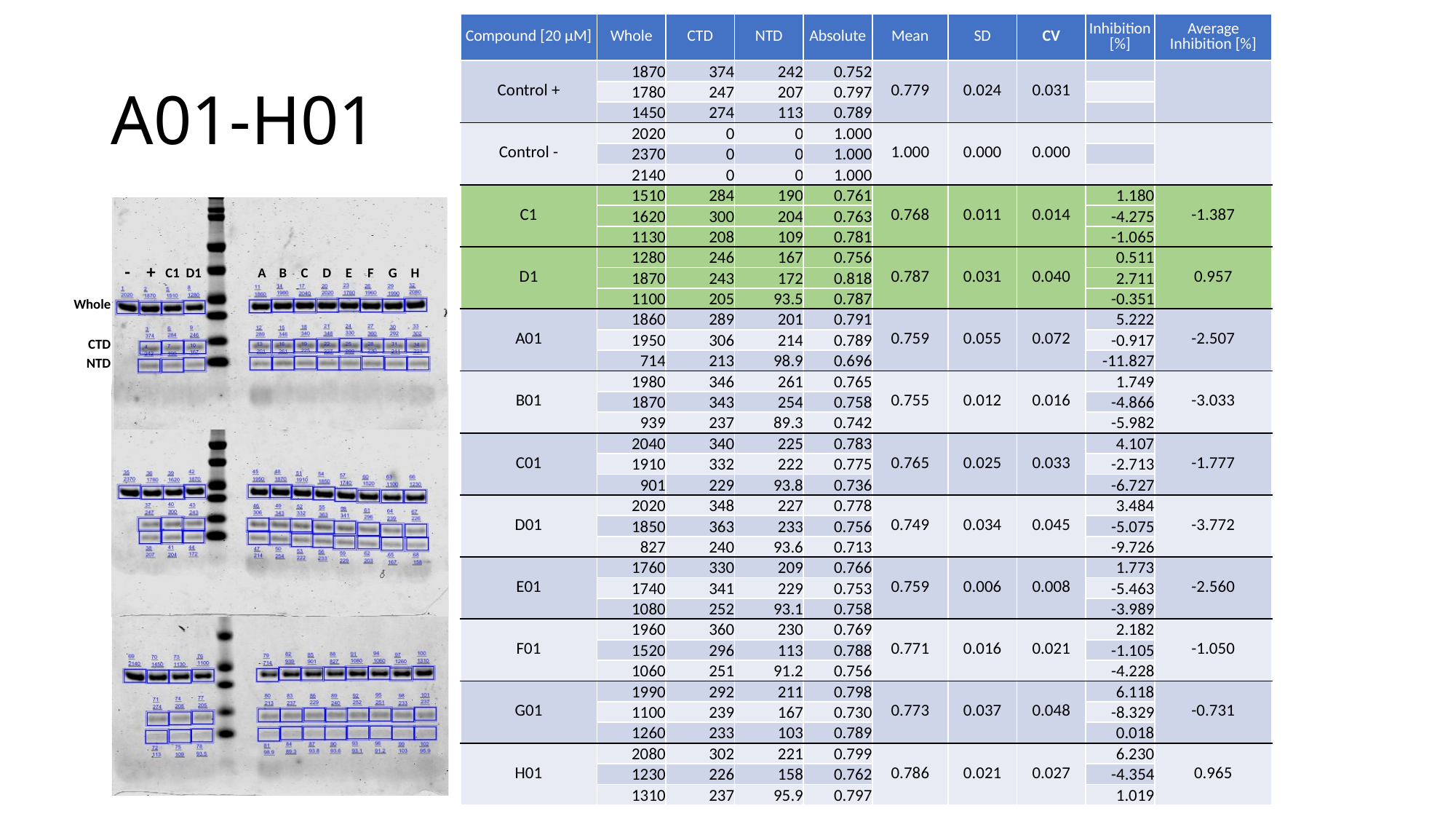

| Compound [20 µM] | Whole | CTD | NTD | Absolute | Mean | SD | CV | Inhibition [%] | Average Inhibition [%] |
| --- | --- | --- | --- | --- | --- | --- | --- | --- | --- |
| Control + | 1870 | 374 | 242 | 0.752 | 0.779 | 0.024 | 0.031 | | |
| | 1780 | 247 | 207 | 0.797 | | | | | |
| | 1450 | 274 | 113 | 0.789 | | | | | |
| Control - | 2020 | 0 | 0 | 1.000 | 1.000 | 0.000 | 0.000 | | |
| | 2370 | 0 | 0 | 1.000 | | | | | |
| | 2140 | 0 | 0 | 1.000 | | | | | |
| C1 | 1510 | 284 | 190 | 0.761 | 0.768 | 0.011 | 0.014 | 1.180 | -1.387 |
| | 1620 | 300 | 204 | 0.763 | | | | -4.275 | |
| | 1130 | 208 | 109 | 0.781 | | | | -1.065 | |
| D1 | 1280 | 246 | 167 | 0.756 | 0.787 | 0.031 | 0.040 | 0.511 | 0.957 |
| | 1870 | 243 | 172 | 0.818 | | | | 2.711 | |
| | 1100 | 205 | 93.5 | 0.787 | | | | -0.351 | |
| A01 | 1860 | 289 | 201 | 0.791 | 0.759 | 0.055 | 0.072 | 5.222 | -2.507 |
| | 1950 | 306 | 214 | 0.789 | | | | -0.917 | |
| | 714 | 213 | 98.9 | 0.696 | | | | -11.827 | |
| B01 | 1980 | 346 | 261 | 0.765 | 0.755 | 0.012 | 0.016 | 1.749 | -3.033 |
| | 1870 | 343 | 254 | 0.758 | | | | -4.866 | |
| | 939 | 237 | 89.3 | 0.742 | | | | -5.982 | |
| C01 | 2040 | 340 | 225 | 0.783 | 0.765 | 0.025 | 0.033 | 4.107 | -1.777 |
| | 1910 | 332 | 222 | 0.775 | | | | -2.713 | |
| | 901 | 229 | 93.8 | 0.736 | | | | -6.727 | |
| D01 | 2020 | 348 | 227 | 0.778 | 0.749 | 0.034 | 0.045 | 3.484 | -3.772 |
| | 1850 | 363 | 233 | 0.756 | | | | -5.075 | |
| | 827 | 240 | 93.6 | 0.713 | | | | -9.726 | |
| E01 | 1760 | 330 | 209 | 0.766 | 0.759 | 0.006 | 0.008 | 1.773 | -2.560 |
| | 1740 | 341 | 229 | 0.753 | | | | -5.463 | |
| | 1080 | 252 | 93.1 | 0.758 | | | | -3.989 | |
| F01 | 1960 | 360 | 230 | 0.769 | 0.771 | 0.016 | 0.021 | 2.182 | -1.050 |
| | 1520 | 296 | 113 | 0.788 | | | | -1.105 | |
| | 1060 | 251 | 91.2 | 0.756 | | | | -4.228 | |
| G01 | 1990 | 292 | 211 | 0.798 | 0.773 | 0.037 | 0.048 | 6.118 | -0.731 |
| | 1100 | 239 | 167 | 0.730 | | | | -8.329 | |
| | 1260 | 233 | 103 | 0.789 | | | | 0.018 | |
| H01 | 2080 | 302 | 221 | 0.799 | 0.786 | 0.021 | 0.027 | 6.230 | 0.965 |
| | 1230 | 226 | 158 | 0.762 | | | | -4.354 | |
| | 1310 | 237 | 95.9 | 0.797 | | | | 1.019 | |
# A01-H01
| A | B | C | D | E | F | G | H |
| --- | --- | --- | --- | --- | --- | --- | --- |
| - | + | C1 | D1 |
| --- | --- | --- | --- |
| Whole |
| --- |
| |
| CTD |
| NTD |

## Slide 4
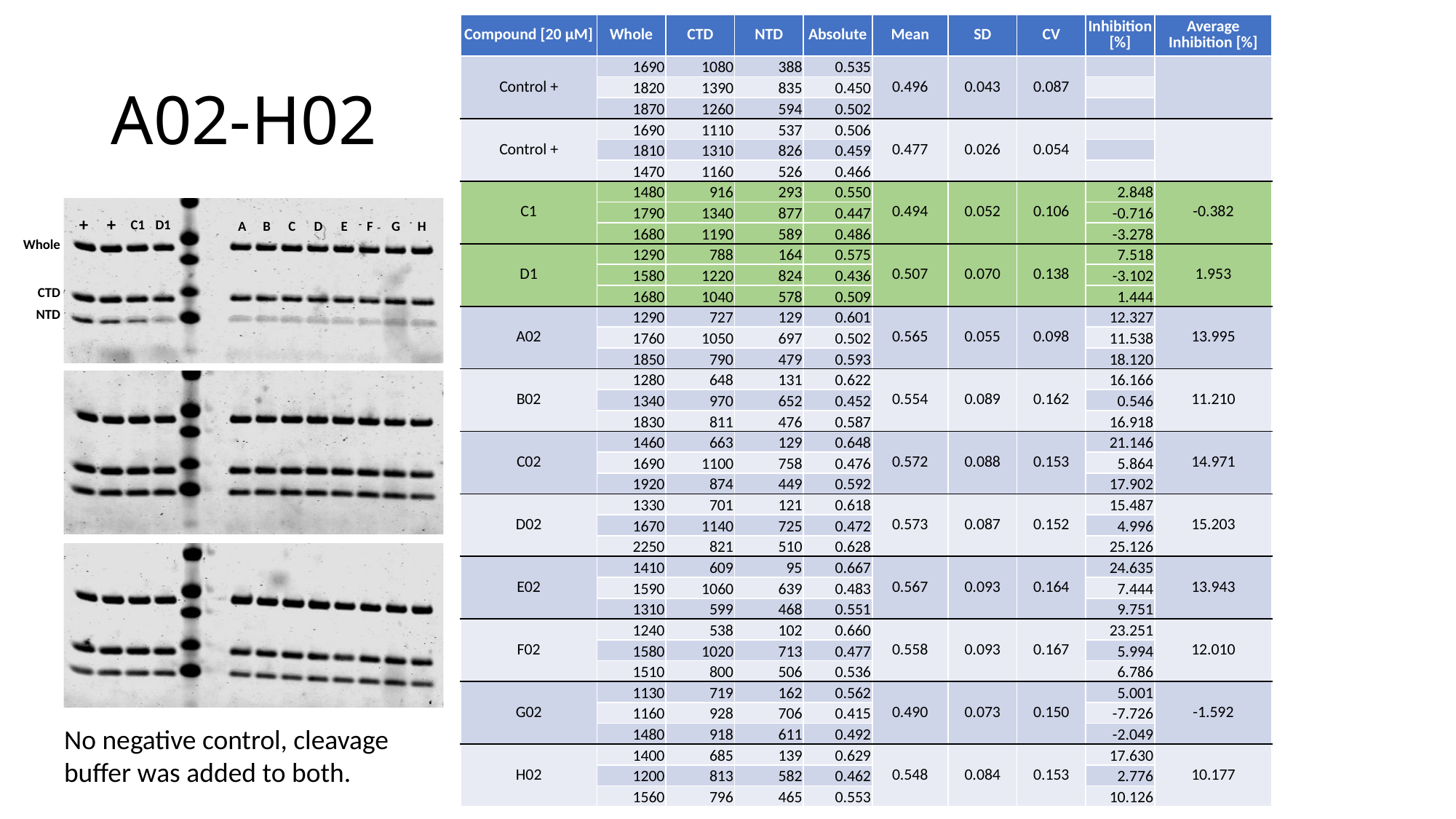

| Compound [20 µM] | Whole | CTD | NTD | Absolute | Mean | SD | CV | Inhibition [%] | Average Inhibition [%] |
| --- | --- | --- | --- | --- | --- | --- | --- | --- | --- |
| Control + | 1690 | 1080 | 388 | 0.535 | 0.496 | 0.043 | 0.087 | | |
| | 1820 | 1390 | 835 | 0.450 | | | | | |
| | 1870 | 1260 | 594 | 0.502 | | | | | |
| Control + | 1690 | 1110 | 537 | 0.506 | 0.477 | 0.026 | 0.054 | | |
| | 1810 | 1310 | 826 | 0.459 | | | | | |
| | 1470 | 1160 | 526 | 0.466 | | | | | |
| C1 | 1480 | 916 | 293 | 0.550 | 0.494 | 0.052 | 0.106 | 2.848 | -0.382 |
| | 1790 | 1340 | 877 | 0.447 | | | | -0.716 | |
| | 1680 | 1190 | 589 | 0.486 | | | | -3.278 | |
| D1 | 1290 | 788 | 164 | 0.575 | 0.507 | 0.070 | 0.138 | 7.518 | 1.953 |
| | 1580 | 1220 | 824 | 0.436 | | | | -3.102 | |
| | 1680 | 1040 | 578 | 0.509 | | | | 1.444 | |
| A02 | 1290 | 727 | 129 | 0.601 | 0.565 | 0.055 | 0.098 | 12.327 | 13.995 |
| | 1760 | 1050 | 697 | 0.502 | | | | 11.538 | |
| | 1850 | 790 | 479 | 0.593 | | | | 18.120 | |
| B02 | 1280 | 648 | 131 | 0.622 | 0.554 | 0.089 | 0.162 | 16.166 | 11.210 |
| | 1340 | 970 | 652 | 0.452 | | | | 0.546 | |
| | 1830 | 811 | 476 | 0.587 | | | | 16.918 | |
| C02 | 1460 | 663 | 129 | 0.648 | 0.572 | 0.088 | 0.153 | 21.146 | 14.971 |
| | 1690 | 1100 | 758 | 0.476 | | | | 5.864 | |
| | 1920 | 874 | 449 | 0.592 | | | | 17.902 | |
| D02 | 1330 | 701 | 121 | 0.618 | 0.573 | 0.087 | 0.152 | 15.487 | 15.203 |
| | 1670 | 1140 | 725 | 0.472 | | | | 4.996 | |
| | 2250 | 821 | 510 | 0.628 | | | | 25.126 | |
| E02 | 1410 | 609 | 95 | 0.667 | 0.567 | 0.093 | 0.164 | 24.635 | 13.943 |
| | 1590 | 1060 | 639 | 0.483 | | | | 7.444 | |
| | 1310 | 599 | 468 | 0.551 | | | | 9.751 | |
| F02 | 1240 | 538 | 102 | 0.660 | 0.558 | 0.093 | 0.167 | 23.251 | 12.010 |
| | 1580 | 1020 | 713 | 0.477 | | | | 5.994 | |
| | 1510 | 800 | 506 | 0.536 | | | | 6.786 | |
| G02 | 1130 | 719 | 162 | 0.562 | 0.490 | 0.073 | 0.150 | 5.001 | -1.592 |
| | 1160 | 928 | 706 | 0.415 | | | | -7.726 | |
| | 1480 | 918 | 611 | 0.492 | | | | -2.049 | |
| H02 | 1400 | 685 | 139 | 0.629 | 0.548 | 0.084 | 0.153 | 17.630 | 10.177 |
| | 1200 | 813 | 582 | 0.462 | | | | 2.776 | |
| | 1560 | 796 | 465 | 0.553 | | | | 10.126 | |
# A02-H02
| + | + | C1 | D1 |
| --- | --- | --- | --- |
| A | B | C | D | E | F | G | H |
| --- | --- | --- | --- | --- | --- | --- | --- |
| Whole |
| --- |
| |
| CTD |
| NTD |
No negative control, cleavage buffer was added to both.

## Slide 5
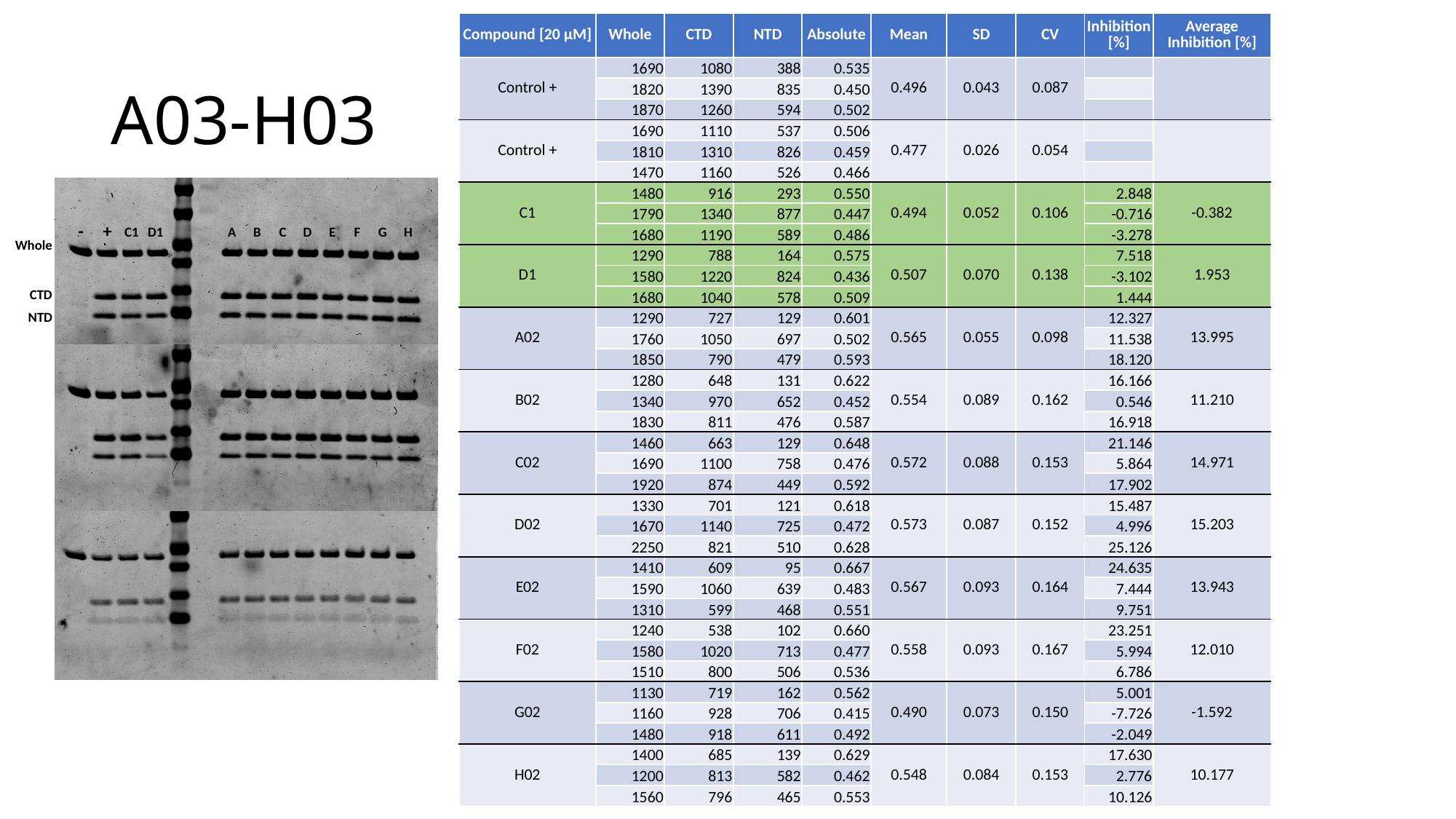

| Compound [20 µM] | Whole | CTD | NTD | Absolute | Mean | SD | CV | Inhibition [%] | Average Inhibition [%] |
| --- | --- | --- | --- | --- | --- | --- | --- | --- | --- |
| Control + | 1690 | 1080 | 388 | 0.535 | 0.496 | 0.043 | 0.087 | | |
| | 1820 | 1390 | 835 | 0.450 | | | | | |
| | 1870 | 1260 | 594 | 0.502 | | | | | |
| Control + | 1690 | 1110 | 537 | 0.506 | 0.477 | 0.026 | 0.054 | | |
| | 1810 | 1310 | 826 | 0.459 | | | | | |
| | 1470 | 1160 | 526 | 0.466 | | | | | |
| C1 | 1480 | 916 | 293 | 0.550 | 0.494 | 0.052 | 0.106 | 2.848 | -0.382 |
| | 1790 | 1340 | 877 | 0.447 | | | | -0.716 | |
| | 1680 | 1190 | 589 | 0.486 | | | | -3.278 | |
| D1 | 1290 | 788 | 164 | 0.575 | 0.507 | 0.070 | 0.138 | 7.518 | 1.953 |
| | 1580 | 1220 | 824 | 0.436 | | | | -3.102 | |
| | 1680 | 1040 | 578 | 0.509 | | | | 1.444 | |
| A02 | 1290 | 727 | 129 | 0.601 | 0.565 | 0.055 | 0.098 | 12.327 | 13.995 |
| | 1760 | 1050 | 697 | 0.502 | | | | 11.538 | |
| | 1850 | 790 | 479 | 0.593 | | | | 18.120 | |
| B02 | 1280 | 648 | 131 | 0.622 | 0.554 | 0.089 | 0.162 | 16.166 | 11.210 |
| | 1340 | 970 | 652 | 0.452 | | | | 0.546 | |
| | 1830 | 811 | 476 | 0.587 | | | | 16.918 | |
| C02 | 1460 | 663 | 129 | 0.648 | 0.572 | 0.088 | 0.153 | 21.146 | 14.971 |
| | 1690 | 1100 | 758 | 0.476 | | | | 5.864 | |
| | 1920 | 874 | 449 | 0.592 | | | | 17.902 | |
| D02 | 1330 | 701 | 121 | 0.618 | 0.573 | 0.087 | 0.152 | 15.487 | 15.203 |
| | 1670 | 1140 | 725 | 0.472 | | | | 4.996 | |
| | 2250 | 821 | 510 | 0.628 | | | | 25.126 | |
| E02 | 1410 | 609 | 95 | 0.667 | 0.567 | 0.093 | 0.164 | 24.635 | 13.943 |
| | 1590 | 1060 | 639 | 0.483 | | | | 7.444 | |
| | 1310 | 599 | 468 | 0.551 | | | | 9.751 | |
| F02 | 1240 | 538 | 102 | 0.660 | 0.558 | 0.093 | 0.167 | 23.251 | 12.010 |
| | 1580 | 1020 | 713 | 0.477 | | | | 5.994 | |
| | 1510 | 800 | 506 | 0.536 | | | | 6.786 | |
| G02 | 1130 | 719 | 162 | 0.562 | 0.490 | 0.073 | 0.150 | 5.001 | -1.592 |
| | 1160 | 928 | 706 | 0.415 | | | | -7.726 | |
| | 1480 | 918 | 611 | 0.492 | | | | -2.049 | |
| H02 | 1400 | 685 | 139 | 0.629 | 0.548 | 0.084 | 0.153 | 17.630 | 10.177 |
| | 1200 | 813 | 582 | 0.462 | | | | 2.776 | |
| | 1560 | 796 | 465 | 0.553 | | | | 10.126 | |
# A03-H03
| - | + | C1 | D1 |
| --- | --- | --- | --- |
| A | B | C | D | E | F | G | H |
| --- | --- | --- | --- | --- | --- | --- | --- |
| Whole |
| --- |
| |
| CTD |
| NTD |

## Slide 6
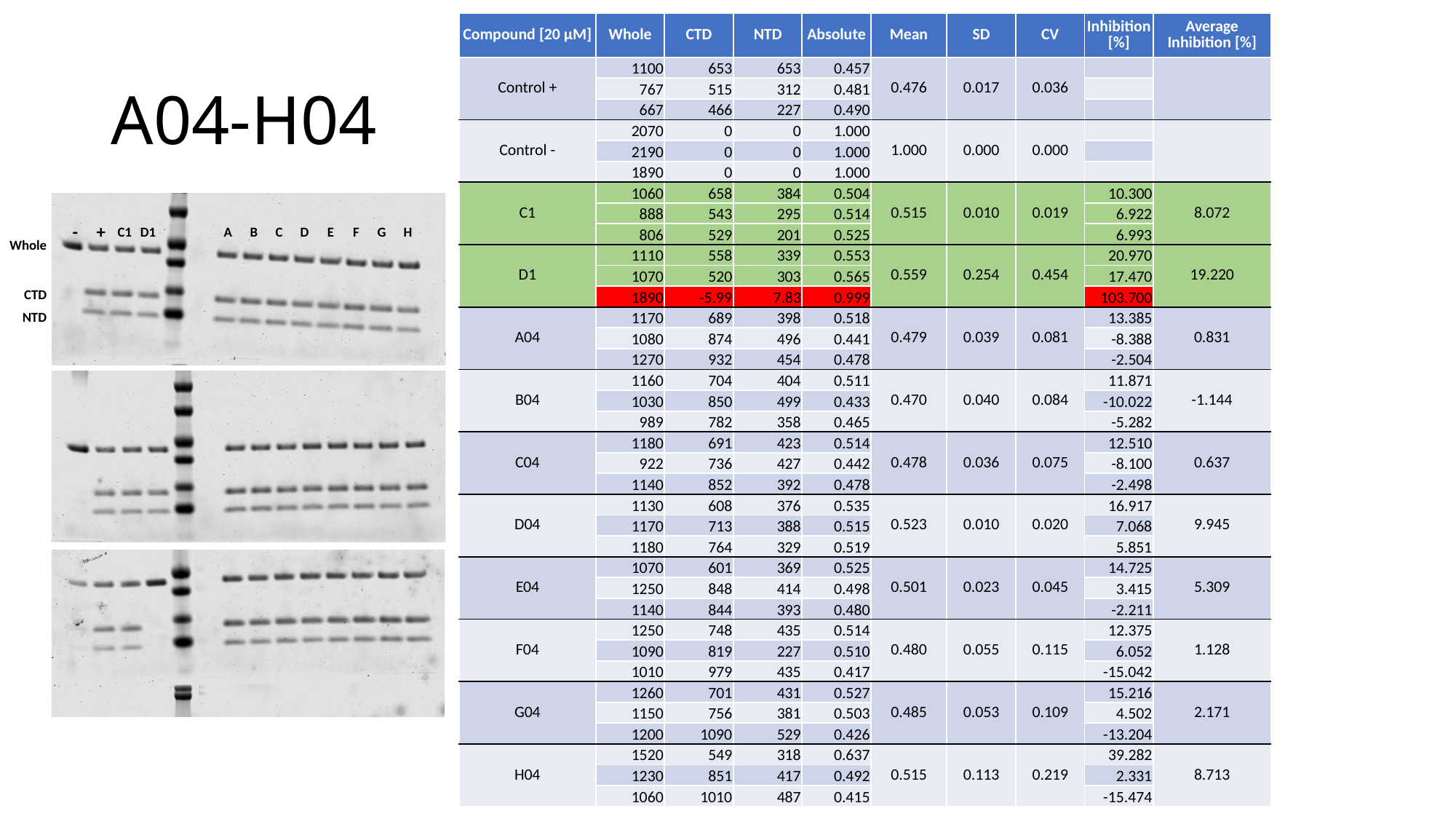

| Compound [20 µM] | Whole | CTD | NTD | Absolute | Mean | SD | CV | Inhibition [%] | Average Inhibition [%] |
| --- | --- | --- | --- | --- | --- | --- | --- | --- | --- |
| Control + | 1100 | 653 | 653 | 0.457 | 0.476 | 0.017 | 0.036 | | |
| | 767 | 515 | 312 | 0.481 | | | | | |
| | 667 | 466 | 227 | 0.490 | | | | | |
| Control - | 2070 | 0 | 0 | 1.000 | 1.000 | 0.000 | 0.000 | | |
| | 2190 | 0 | 0 | 1.000 | | | | | |
| | 1890 | 0 | 0 | 1.000 | | | | | |
| C1 | 1060 | 658 | 384 | 0.504 | 0.515 | 0.010 | 0.019 | 10.300 | 8.072 |
| | 888 | 543 | 295 | 0.514 | | | | 6.922 | |
| | 806 | 529 | 201 | 0.525 | | | | 6.993 | |
| D1 | 1110 | 558 | 339 | 0.553 | 0.559 | 0.254 | 0.454 | 20.970 | 19.220 |
| | 1070 | 520 | 303 | 0.565 | | | | 17.470 | |
| | 1890 | -5.99 | 7.83 | 0.999 | | | | 103.700 | |
| A04 | 1170 | 689 | 398 | 0.518 | 0.479 | 0.039 | 0.081 | 13.385 | 0.831 |
| | 1080 | 874 | 496 | 0.441 | | | | -8.388 | |
| | 1270 | 932 | 454 | 0.478 | | | | -2.504 | |
| B04 | 1160 | 704 | 404 | 0.511 | 0.470 | 0.040 | 0.084 | 11.871 | -1.144 |
| | 1030 | 850 | 499 | 0.433 | | | | -10.022 | |
| | 989 | 782 | 358 | 0.465 | | | | -5.282 | |
| C04 | 1180 | 691 | 423 | 0.514 | 0.478 | 0.036 | 0.075 | 12.510 | 0.637 |
| | 922 | 736 | 427 | 0.442 | | | | -8.100 | |
| | 1140 | 852 | 392 | 0.478 | | | | -2.498 | |
| D04 | 1130 | 608 | 376 | 0.535 | 0.523 | 0.010 | 0.020 | 16.917 | 9.945 |
| | 1170 | 713 | 388 | 0.515 | | | | 7.068 | |
| | 1180 | 764 | 329 | 0.519 | | | | 5.851 | |
| E04 | 1070 | 601 | 369 | 0.525 | 0.501 | 0.023 | 0.045 | 14.725 | 5.309 |
| | 1250 | 848 | 414 | 0.498 | | | | 3.415 | |
| | 1140 | 844 | 393 | 0.480 | | | | -2.211 | |
| F04 | 1250 | 748 | 435 | 0.514 | 0.480 | 0.055 | 0.115 | 12.375 | 1.128 |
| | 1090 | 819 | 227 | 0.510 | | | | 6.052 | |
| | 1010 | 979 | 435 | 0.417 | | | | -15.042 | |
| G04 | 1260 | 701 | 431 | 0.527 | 0.485 | 0.053 | 0.109 | 15.216 | 2.171 |
| | 1150 | 756 | 381 | 0.503 | | | | 4.502 | |
| | 1200 | 1090 | 529 | 0.426 | | | | -13.204 | |
| H04 | 1520 | 549 | 318 | 0.637 | 0.515 | 0.113 | 0.219 | 39.282 | 8.713 |
| | 1230 | 851 | 417 | 0.492 | | | | 2.331 | |
| | 1060 | 1010 | 487 | 0.415 | | | | -15.474 | |
# A04-H04
| - | + | C1 | D1 |
| --- | --- | --- | --- |
| A | B | C | D | E | F | G | H |
| --- | --- | --- | --- | --- | --- | --- | --- |
| Whole |
| --- |
| |
| CTD |
| NTD |

## Slide 7
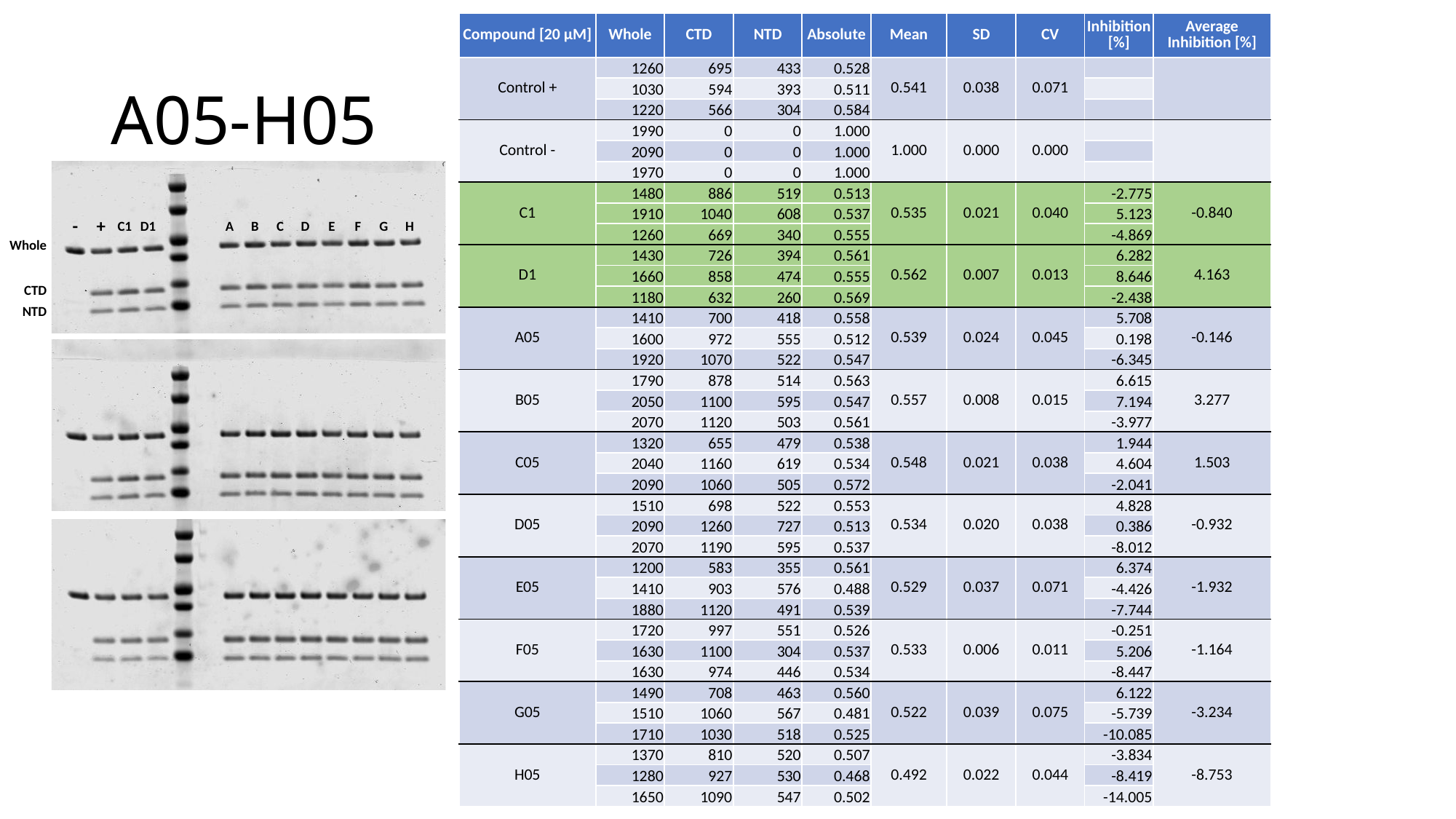

| Compound [20 µM] | Whole | CTD | NTD | Absolute | Mean | SD | CV | Inhibition [%] | Average Inhibition [%] |
| --- | --- | --- | --- | --- | --- | --- | --- | --- | --- |
| Control + | 1260 | 695 | 433 | 0.528 | 0.541 | 0.038 | 0.071 | | |
| | 1030 | 594 | 393 | 0.511 | | | | | |
| | 1220 | 566 | 304 | 0.584 | | | | | |
| Control - | 1990 | 0 | 0 | 1.000 | 1.000 | 0.000 | 0.000 | | |
| | 2090 | 0 | 0 | 1.000 | | | | | |
| | 1970 | 0 | 0 | 1.000 | | | | | |
| C1 | 1480 | 886 | 519 | 0.513 | 0.535 | 0.021 | 0.040 | -2.775 | -0.840 |
| | 1910 | 1040 | 608 | 0.537 | | | | 5.123 | |
| | 1260 | 669 | 340 | 0.555 | | | | -4.869 | |
| D1 | 1430 | 726 | 394 | 0.561 | 0.562 | 0.007 | 0.013 | 6.282 | 4.163 |
| | 1660 | 858 | 474 | 0.555 | | | | 8.646 | |
| | 1180 | 632 | 260 | 0.569 | | | | -2.438 | |
| A05 | 1410 | 700 | 418 | 0.558 | 0.539 | 0.024 | 0.045 | 5.708 | -0.146 |
| | 1600 | 972 | 555 | 0.512 | | | | 0.198 | |
| | 1920 | 1070 | 522 | 0.547 | | | | -6.345 | |
| B05 | 1790 | 878 | 514 | 0.563 | 0.557 | 0.008 | 0.015 | 6.615 | 3.277 |
| | 2050 | 1100 | 595 | 0.547 | | | | 7.194 | |
| | 2070 | 1120 | 503 | 0.561 | | | | -3.977 | |
| C05 | 1320 | 655 | 479 | 0.538 | 0.548 | 0.021 | 0.038 | 1.944 | 1.503 |
| | 2040 | 1160 | 619 | 0.534 | | | | 4.604 | |
| | 2090 | 1060 | 505 | 0.572 | | | | -2.041 | |
| D05 | 1510 | 698 | 522 | 0.553 | 0.534 | 0.020 | 0.038 | 4.828 | -0.932 |
| | 2090 | 1260 | 727 | 0.513 | | | | 0.386 | |
| | 2070 | 1190 | 595 | 0.537 | | | | -8.012 | |
| E05 | 1200 | 583 | 355 | 0.561 | 0.529 | 0.037 | 0.071 | 6.374 | -1.932 |
| | 1410 | 903 | 576 | 0.488 | | | | -4.426 | |
| | 1880 | 1120 | 491 | 0.539 | | | | -7.744 | |
| F05 | 1720 | 997 | 551 | 0.526 | 0.533 | 0.006 | 0.011 | -0.251 | -1.164 |
| | 1630 | 1100 | 304 | 0.537 | | | | 5.206 | |
| | 1630 | 974 | 446 | 0.534 | | | | -8.447 | |
| G05 | 1490 | 708 | 463 | 0.560 | 0.522 | 0.039 | 0.075 | 6.122 | -3.234 |
| | 1510 | 1060 | 567 | 0.481 | | | | -5.739 | |
| | 1710 | 1030 | 518 | 0.525 | | | | -10.085 | |
| H05 | 1370 | 810 | 520 | 0.507 | 0.492 | 0.022 | 0.044 | -3.834 | -8.753 |
| | 1280 | 927 | 530 | 0.468 | | | | -8.419 | |
| | 1650 | 1090 | 547 | 0.502 | | | | -14.005 | |
# A05-H05
| - | + | C1 | D1 |
| --- | --- | --- | --- |
| A | B | C | D | E | F | G | H |
| --- | --- | --- | --- | --- | --- | --- | --- |
| Whole |
| --- |
| |
| CTD |
| NTD |

## Slide 8
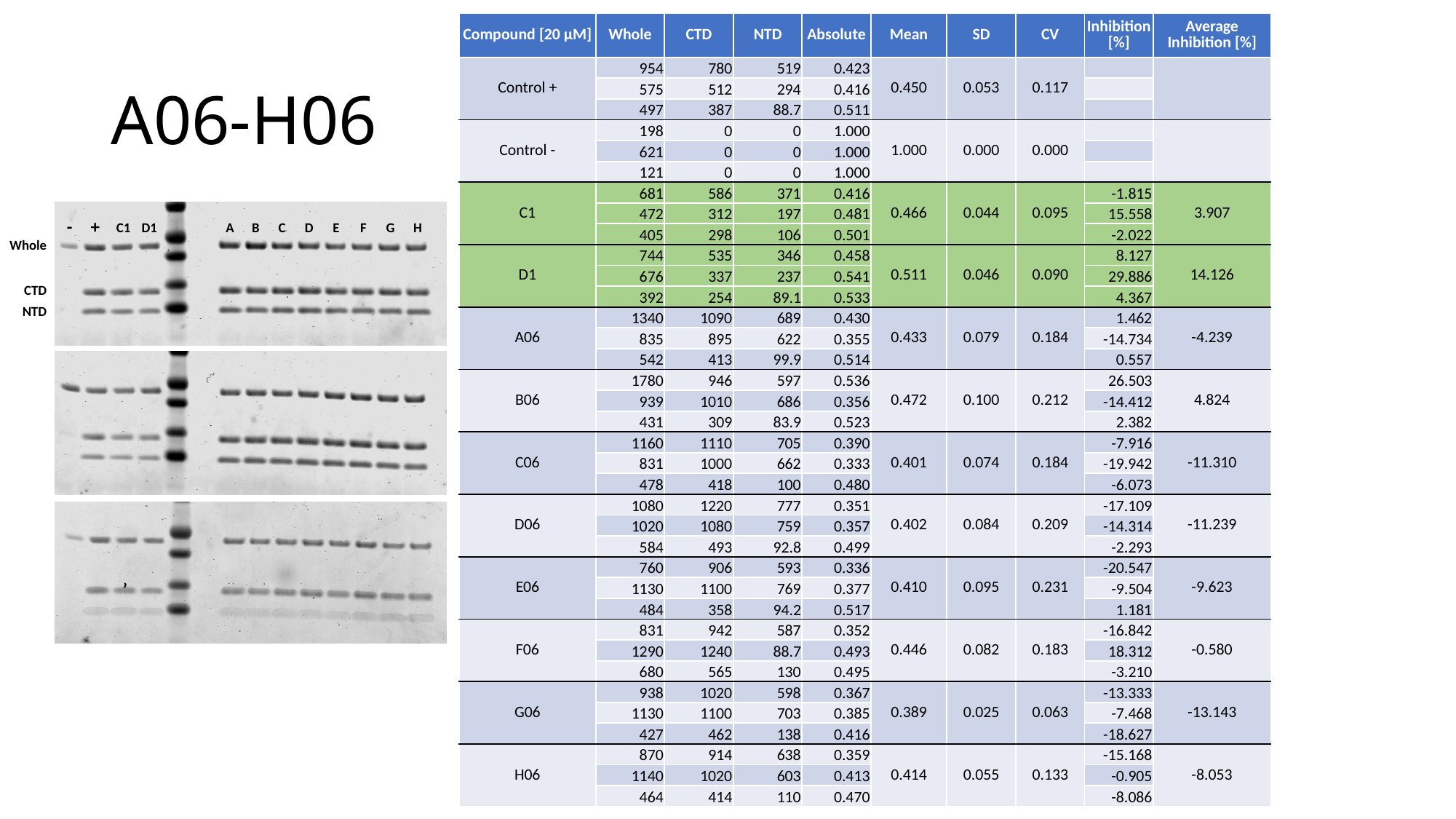

| Compound [20 µM] | Whole | CTD | NTD | Absolute | Mean | SD | CV | Inhibition [%] | Average Inhibition [%] |
| --- | --- | --- | --- | --- | --- | --- | --- | --- | --- |
| Control + | 954 | 780 | 519 | 0.423 | 0.450 | 0.053 | 0.117 | | |
| | 575 | 512 | 294 | 0.416 | | | | | |
| | 497 | 387 | 88.7 | 0.511 | | | | | |
| Control - | 198 | 0 | 0 | 1.000 | 1.000 | 0.000 | 0.000 | | |
| | 621 | 0 | 0 | 1.000 | | | | | |
| | 121 | 0 | 0 | 1.000 | | | | | |
| C1 | 681 | 586 | 371 | 0.416 | 0.466 | 0.044 | 0.095 | -1.815 | 3.907 |
| | 472 | 312 | 197 | 0.481 | | | | 15.558 | |
| | 405 | 298 | 106 | 0.501 | | | | -2.022 | |
| D1 | 744 | 535 | 346 | 0.458 | 0.511 | 0.046 | 0.090 | 8.127 | 14.126 |
| | 676 | 337 | 237 | 0.541 | | | | 29.886 | |
| | 392 | 254 | 89.1 | 0.533 | | | | 4.367 | |
| A06 | 1340 | 1090 | 689 | 0.430 | 0.433 | 0.079 | 0.184 | 1.462 | -4.239 |
| | 835 | 895 | 622 | 0.355 | | | | -14.734 | |
| | 542 | 413 | 99.9 | 0.514 | | | | 0.557 | |
| B06 | 1780 | 946 | 597 | 0.536 | 0.472 | 0.100 | 0.212 | 26.503 | 4.824 |
| | 939 | 1010 | 686 | 0.356 | | | | -14.412 | |
| | 431 | 309 | 83.9 | 0.523 | | | | 2.382 | |
| C06 | 1160 | 1110 | 705 | 0.390 | 0.401 | 0.074 | 0.184 | -7.916 | -11.310 |
| | 831 | 1000 | 662 | 0.333 | | | | -19.942 | |
| | 478 | 418 | 100 | 0.480 | | | | -6.073 | |
| D06 | 1080 | 1220 | 777 | 0.351 | 0.402 | 0.084 | 0.209 | -17.109 | -11.239 |
| | 1020 | 1080 | 759 | 0.357 | | | | -14.314 | |
| | 584 | 493 | 92.8 | 0.499 | | | | -2.293 | |
| E06 | 760 | 906 | 593 | 0.336 | 0.410 | 0.095 | 0.231 | -20.547 | -9.623 |
| | 1130 | 1100 | 769 | 0.377 | | | | -9.504 | |
| | 484 | 358 | 94.2 | 0.517 | | | | 1.181 | |
| F06 | 831 | 942 | 587 | 0.352 | 0.446 | 0.082 | 0.183 | -16.842 | -0.580 |
| | 1290 | 1240 | 88.7 | 0.493 | | | | 18.312 | |
| | 680 | 565 | 130 | 0.495 | | | | -3.210 | |
| G06 | 938 | 1020 | 598 | 0.367 | 0.389 | 0.025 | 0.063 | -13.333 | -13.143 |
| | 1130 | 1100 | 703 | 0.385 | | | | -7.468 | |
| | 427 | 462 | 138 | 0.416 | | | | -18.627 | |
| H06 | 870 | 914 | 638 | 0.359 | 0.414 | 0.055 | 0.133 | -15.168 | -8.053 |
| | 1140 | 1020 | 603 | 0.413 | | | | -0.905 | |
| | 464 | 414 | 110 | 0.470 | | | | -8.086 | |
# A06-H06
| - | + | C1 | D1 |
| --- | --- | --- | --- |
| A | B | C | D | E | F | G | H |
| --- | --- | --- | --- | --- | --- | --- | --- |
| Whole |
| --- |
| |
| CTD |
| NTD |

## Slide 9
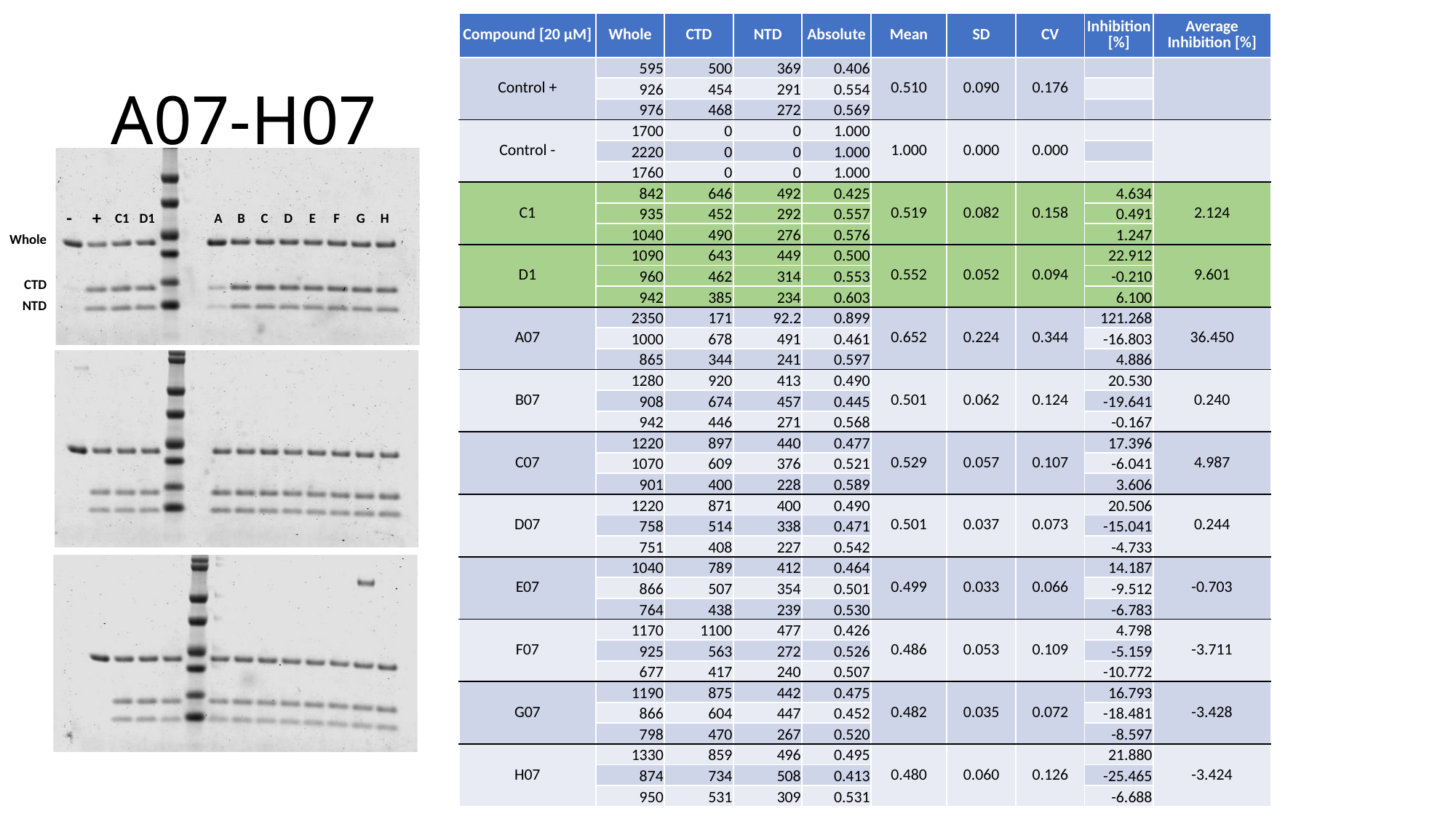

| Compound [20 µM] | Whole | CTD | NTD | Absolute | Mean | SD | CV | Inhibition [%] | Average Inhibition [%] |
| --- | --- | --- | --- | --- | --- | --- | --- | --- | --- |
| Control + | 595 | 500 | 369 | 0.406 | 0.510 | 0.090 | 0.176 | | |
| | 926 | 454 | 291 | 0.554 | | | | | |
| | 976 | 468 | 272 | 0.569 | | | | | |
| Control - | 1700 | 0 | 0 | 1.000 | 1.000 | 0.000 | 0.000 | | |
| | 2220 | 0 | 0 | 1.000 | | | | | |
| | 1760 | 0 | 0 | 1.000 | | | | | |
| C1 | 842 | 646 | 492 | 0.425 | 0.519 | 0.082 | 0.158 | 4.634 | 2.124 |
| | 935 | 452 | 292 | 0.557 | | | | 0.491 | |
| | 1040 | 490 | 276 | 0.576 | | | | 1.247 | |
| D1 | 1090 | 643 | 449 | 0.500 | 0.552 | 0.052 | 0.094 | 22.912 | 9.601 |
| | 960 | 462 | 314 | 0.553 | | | | -0.210 | |
| | 942 | 385 | 234 | 0.603 | | | | 6.100 | |
| A07 | 2350 | 171 | 92.2 | 0.899 | 0.652 | 0.224 | 0.344 | 121.268 | 36.450 |
| | 1000 | 678 | 491 | 0.461 | | | | -16.803 | |
| | 865 | 344 | 241 | 0.597 | | | | 4.886 | |
| B07 | 1280 | 920 | 413 | 0.490 | 0.501 | 0.062 | 0.124 | 20.530 | 0.240 |
| | 908 | 674 | 457 | 0.445 | | | | -19.641 | |
| | 942 | 446 | 271 | 0.568 | | | | -0.167 | |
| C07 | 1220 | 897 | 440 | 0.477 | 0.529 | 0.057 | 0.107 | 17.396 | 4.987 |
| | 1070 | 609 | 376 | 0.521 | | | | -6.041 | |
| | 901 | 400 | 228 | 0.589 | | | | 3.606 | |
| D07 | 1220 | 871 | 400 | 0.490 | 0.501 | 0.037 | 0.073 | 20.506 | 0.244 |
| | 758 | 514 | 338 | 0.471 | | | | -15.041 | |
| | 751 | 408 | 227 | 0.542 | | | | -4.733 | |
| E07 | 1040 | 789 | 412 | 0.464 | 0.499 | 0.033 | 0.066 | 14.187 | -0.703 |
| | 866 | 507 | 354 | 0.501 | | | | -9.512 | |
| | 764 | 438 | 239 | 0.530 | | | | -6.783 | |
| F07 | 1170 | 1100 | 477 | 0.426 | 0.486 | 0.053 | 0.109 | 4.798 | -3.711 |
| | 925 | 563 | 272 | 0.526 | | | | -5.159 | |
| | 677 | 417 | 240 | 0.507 | | | | -10.772 | |
| G07 | 1190 | 875 | 442 | 0.475 | 0.482 | 0.035 | 0.072 | 16.793 | -3.428 |
| | 866 | 604 | 447 | 0.452 | | | | -18.481 | |
| | 798 | 470 | 267 | 0.520 | | | | -8.597 | |
| H07 | 1330 | 859 | 496 | 0.495 | 0.480 | 0.060 | 0.126 | 21.880 | -3.424 |
| | 874 | 734 | 508 | 0.413 | | | | -25.465 | |
| | 950 | 531 | 309 | 0.531 | | | | -6.688 | |
# A07-H07
| - | + | C1 | D1 |
| --- | --- | --- | --- |
| A | B | C | D | E | F | G | H |
| --- | --- | --- | --- | --- | --- | --- | --- |
| Whole |
| --- |
| |
| CTD |
| NTD |

## Slide 10
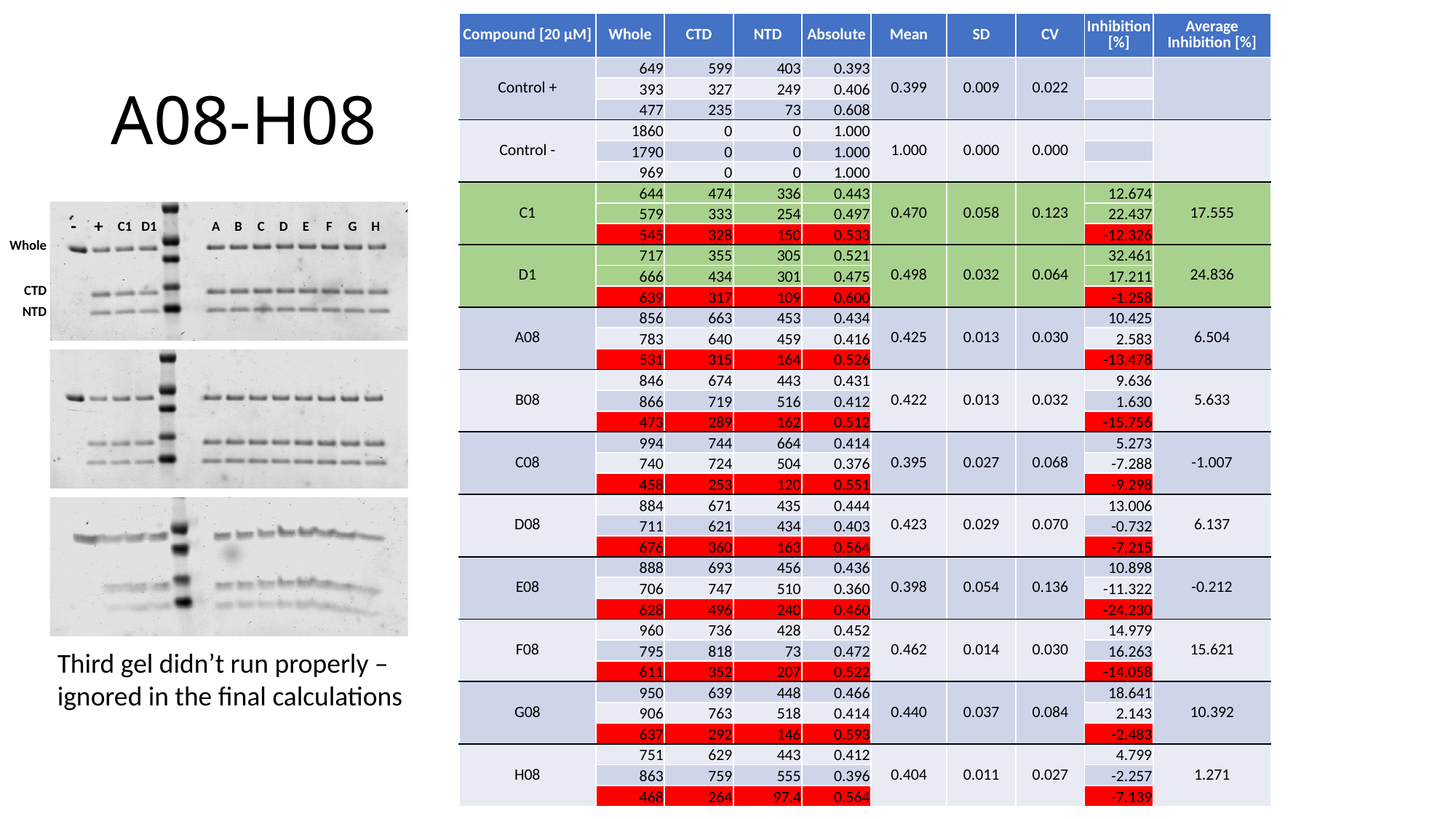

| Compound [20 µM] | Whole | CTD | NTD | Absolute | Mean | SD | CV | Inhibition [%] | Average Inhibition [%] |
| --- | --- | --- | --- | --- | --- | --- | --- | --- | --- |
| Control + | 649 | 599 | 403 | 0.393 | 0.399 | 0.009 | 0.022 | | |
| | 393 | 327 | 249 | 0.406 | | | | | |
| | 477 | 235 | 73 | 0.608 | | | | | |
| Control - | 1860 | 0 | 0 | 1.000 | 1.000 | 0.000 | 0.000 | | |
| | 1790 | 0 | 0 | 1.000 | | | | | |
| | 969 | 0 | 0 | 1.000 | | | | | |
| C1 | 644 | 474 | 336 | 0.443 | 0.470 | 0.058 | 0.123 | 12.674 | 17.555 |
| | 579 | 333 | 254 | 0.497 | | | | 22.437 | |
| | 545 | 328 | 150 | 0.533 | | | | -12.326 | |
| D1 | 717 | 355 | 305 | 0.521 | 0.498 | 0.032 | 0.064 | 32.461 | 24.836 |
| | 666 | 434 | 301 | 0.475 | | | | 17.211 | |
| | 639 | 317 | 109 | 0.600 | | | | -1.258 | |
| A08 | 856 | 663 | 453 | 0.434 | 0.425 | 0.013 | 0.030 | 10.425 | 6.504 |
| | 783 | 640 | 459 | 0.416 | | | | 2.583 | |
| | 531 | 315 | 164 | 0.526 | | | | -13.478 | |
| B08 | 846 | 674 | 443 | 0.431 | 0.422 | 0.013 | 0.032 | 9.636 | 5.633 |
| | 866 | 719 | 516 | 0.412 | | | | 1.630 | |
| | 473 | 289 | 162 | 0.512 | | | | -15.756 | |
| C08 | 994 | 744 | 664 | 0.414 | 0.395 | 0.027 | 0.068 | 5.273 | -1.007 |
| | 740 | 724 | 504 | 0.376 | | | | -7.288 | |
| | 458 | 253 | 120 | 0.551 | | | | -9.298 | |
| D08 | 884 | 671 | 435 | 0.444 | 0.423 | 0.029 | 0.070 | 13.006 | 6.137 |
| | 711 | 621 | 434 | 0.403 | | | | -0.732 | |
| | 676 | 360 | 163 | 0.564 | | | | -7.215 | |
| E08 | 888 | 693 | 456 | 0.436 | 0.398 | 0.054 | 0.136 | 10.898 | -0.212 |
| | 706 | 747 | 510 | 0.360 | | | | -11.322 | |
| | 628 | 496 | 240 | 0.460 | | | | -24.230 | |
| F08 | 960 | 736 | 428 | 0.452 | 0.462 | 0.014 | 0.030 | 14.979 | 15.621 |
| | 795 | 818 | 73 | 0.472 | | | | 16.263 | |
| | 611 | 352 | 207 | 0.522 | | | | -14.058 | |
| G08 | 950 | 639 | 448 | 0.466 | 0.440 | 0.037 | 0.084 | 18.641 | 10.392 |
| | 906 | 763 | 518 | 0.414 | | | | 2.143 | |
| | 637 | 292 | 146 | 0.593 | | | | -2.483 | |
| H08 | 751 | 629 | 443 | 0.412 | 0.404 | 0.011 | 0.027 | 4.799 | 1.271 |
| | 863 | 759 | 555 | 0.396 | | | | -2.257 | |
| | 468 | 264 | 97.4 | 0.564 | | | | -7.139 | |
# A08-H08
| - | + | C1 | D1 |
| --- | --- | --- | --- |
| A | B | C | D | E | F | G | H |
| --- | --- | --- | --- | --- | --- | --- | --- |
| Whole |
| --- |
| |
| CTD |
| NTD |
Third gel didn’t run properly – ignored in the final calculations

## Slide 11
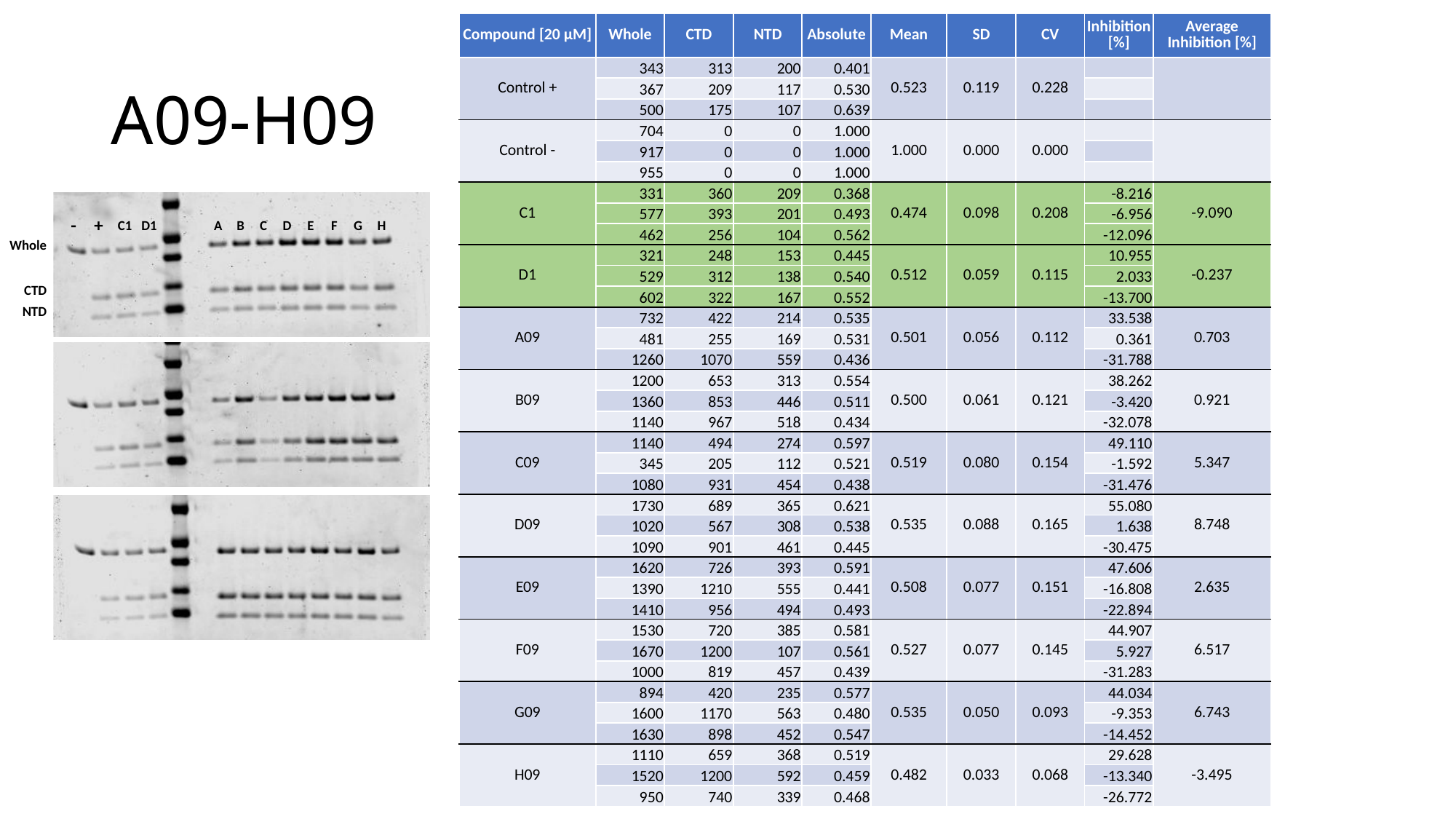

| Compound [20 µM] | Whole | CTD | NTD | Absolute | Mean | SD | CV | Inhibition [%] | Average Inhibition [%] |
| --- | --- | --- | --- | --- | --- | --- | --- | --- | --- |
| Control + | 343 | 313 | 200 | 0.401 | 0.523 | 0.119 | 0.228 | | |
| | 367 | 209 | 117 | 0.530 | | | | | |
| | 500 | 175 | 107 | 0.639 | | | | | |
| Control - | 704 | 0 | 0 | 1.000 | 1.000 | 0.000 | 0.000 | | |
| | 917 | 0 | 0 | 1.000 | | | | | |
| | 955 | 0 | 0 | 1.000 | | | | | |
| C1 | 331 | 360 | 209 | 0.368 | 0.474 | 0.098 | 0.208 | -8.216 | -9.090 |
| | 577 | 393 | 201 | 0.493 | | | | -6.956 | |
| | 462 | 256 | 104 | 0.562 | | | | -12.096 | |
| D1 | 321 | 248 | 153 | 0.445 | 0.512 | 0.059 | 0.115 | 10.955 | -0.237 |
| | 529 | 312 | 138 | 0.540 | | | | 2.033 | |
| | 602 | 322 | 167 | 0.552 | | | | -13.700 | |
| A09 | 732 | 422 | 214 | 0.535 | 0.501 | 0.056 | 0.112 | 33.538 | 0.703 |
| | 481 | 255 | 169 | 0.531 | | | | 0.361 | |
| | 1260 | 1070 | 559 | 0.436 | | | | -31.788 | |
| B09 | 1200 | 653 | 313 | 0.554 | 0.500 | 0.061 | 0.121 | 38.262 | 0.921 |
| | 1360 | 853 | 446 | 0.511 | | | | -3.420 | |
| | 1140 | 967 | 518 | 0.434 | | | | -32.078 | |
| C09 | 1140 | 494 | 274 | 0.597 | 0.519 | 0.080 | 0.154 | 49.110 | 5.347 |
| | 345 | 205 | 112 | 0.521 | | | | -1.592 | |
| | 1080 | 931 | 454 | 0.438 | | | | -31.476 | |
| D09 | 1730 | 689 | 365 | 0.621 | 0.535 | 0.088 | 0.165 | 55.080 | 8.748 |
| | 1020 | 567 | 308 | 0.538 | | | | 1.638 | |
| | 1090 | 901 | 461 | 0.445 | | | | -30.475 | |
| E09 | 1620 | 726 | 393 | 0.591 | 0.508 | 0.077 | 0.151 | 47.606 | 2.635 |
| | 1390 | 1210 | 555 | 0.441 | | | | -16.808 | |
| | 1410 | 956 | 494 | 0.493 | | | | -22.894 | |
| F09 | 1530 | 720 | 385 | 0.581 | 0.527 | 0.077 | 0.145 | 44.907 | 6.517 |
| | 1670 | 1200 | 107 | 0.561 | | | | 5.927 | |
| | 1000 | 819 | 457 | 0.439 | | | | -31.283 | |
| G09 | 894 | 420 | 235 | 0.577 | 0.535 | 0.050 | 0.093 | 44.034 | 6.743 |
| | 1600 | 1170 | 563 | 0.480 | | | | -9.353 | |
| | 1630 | 898 | 452 | 0.547 | | | | -14.452 | |
| H09 | 1110 | 659 | 368 | 0.519 | 0.482 | 0.033 | 0.068 | 29.628 | -3.495 |
| | 1520 | 1200 | 592 | 0.459 | | | | -13.340 | |
| | 950 | 740 | 339 | 0.468 | | | | -26.772 | |
# A09-H09
| - | + | C1 | D1 |
| --- | --- | --- | --- |
| A | B | C | D | E | F | G | H |
| --- | --- | --- | --- | --- | --- | --- | --- |
| Whole |
| --- |
| |
| CTD |
| NTD |

## Slide 12
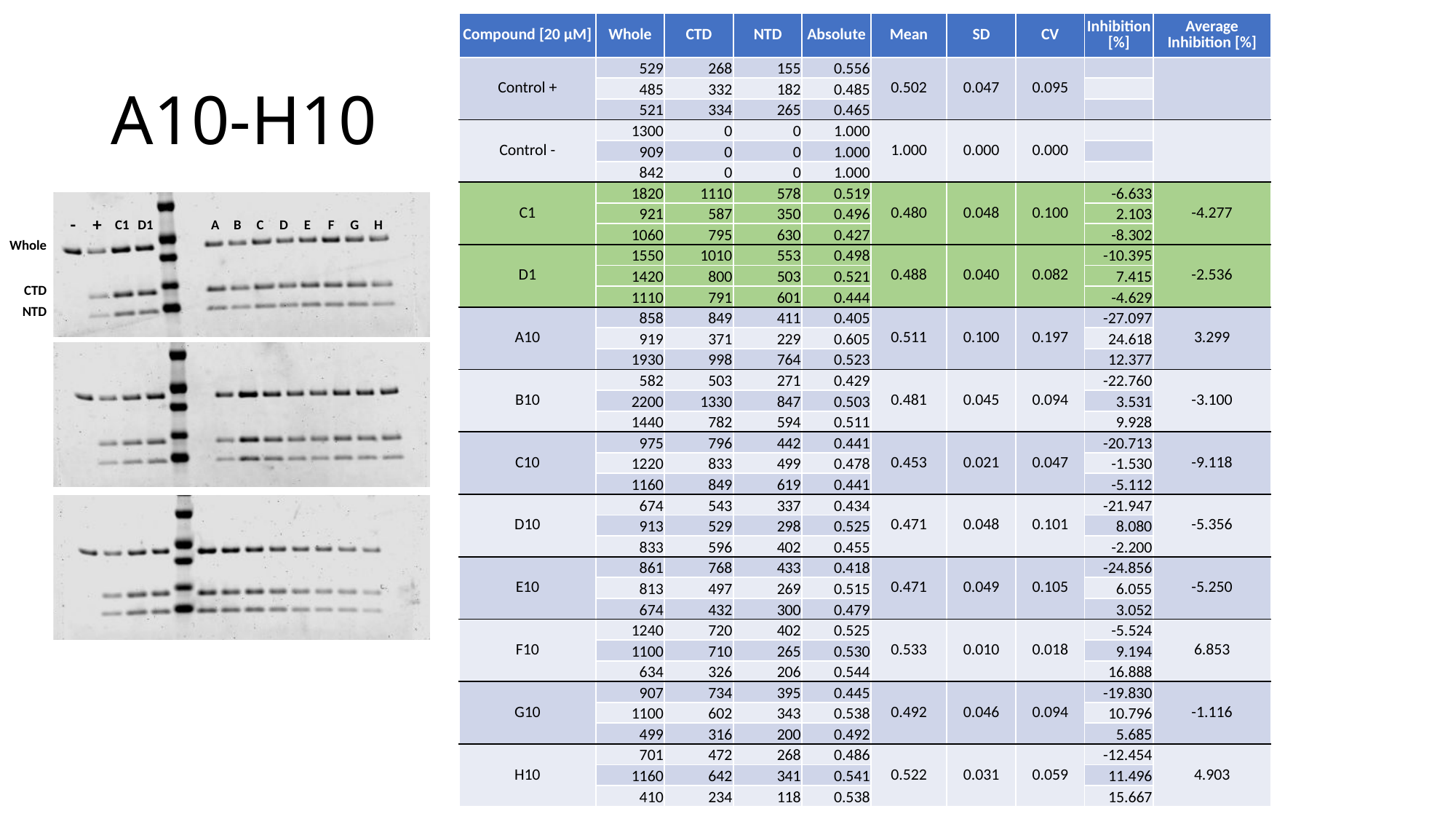

| Compound [20 µM] | Whole | CTD | NTD | Absolute | Mean | SD | CV | Inhibition [%] | Average Inhibition [%] |
| --- | --- | --- | --- | --- | --- | --- | --- | --- | --- |
| Control + | 529 | 268 | 155 | 0.556 | 0.502 | 0.047 | 0.095 | | |
| | 485 | 332 | 182 | 0.485 | | | | | |
| | 521 | 334 | 265 | 0.465 | | | | | |
| Control - | 1300 | 0 | 0 | 1.000 | 1.000 | 0.000 | 0.000 | | |
| | 909 | 0 | 0 | 1.000 | | | | | |
| | 842 | 0 | 0 | 1.000 | | | | | |
| C1 | 1820 | 1110 | 578 | 0.519 | 0.480 | 0.048 | 0.100 | -6.633 | -4.277 |
| | 921 | 587 | 350 | 0.496 | | | | 2.103 | |
| | 1060 | 795 | 630 | 0.427 | | | | -8.302 | |
| D1 | 1550 | 1010 | 553 | 0.498 | 0.488 | 0.040 | 0.082 | -10.395 | -2.536 |
| | 1420 | 800 | 503 | 0.521 | | | | 7.415 | |
| | 1110 | 791 | 601 | 0.444 | | | | -4.629 | |
| A10 | 858 | 849 | 411 | 0.405 | 0.511 | 0.100 | 0.197 | -27.097 | 3.299 |
| | 919 | 371 | 229 | 0.605 | | | | 24.618 | |
| | 1930 | 998 | 764 | 0.523 | | | | 12.377 | |
| B10 | 582 | 503 | 271 | 0.429 | 0.481 | 0.045 | 0.094 | -22.760 | -3.100 |
| | 2200 | 1330 | 847 | 0.503 | | | | 3.531 | |
| | 1440 | 782 | 594 | 0.511 | | | | 9.928 | |
| C10 | 975 | 796 | 442 | 0.441 | 0.453 | 0.021 | 0.047 | -20.713 | -9.118 |
| | 1220 | 833 | 499 | 0.478 | | | | -1.530 | |
| | 1160 | 849 | 619 | 0.441 | | | | -5.112 | |
| D10 | 674 | 543 | 337 | 0.434 | 0.471 | 0.048 | 0.101 | -21.947 | -5.356 |
| | 913 | 529 | 298 | 0.525 | | | | 8.080 | |
| | 833 | 596 | 402 | 0.455 | | | | -2.200 | |
| E10 | 861 | 768 | 433 | 0.418 | 0.471 | 0.049 | 0.105 | -24.856 | -5.250 |
| | 813 | 497 | 269 | 0.515 | | | | 6.055 | |
| | 674 | 432 | 300 | 0.479 | | | | 3.052 | |
| F10 | 1240 | 720 | 402 | 0.525 | 0.533 | 0.010 | 0.018 | -5.524 | 6.853 |
| | 1100 | 710 | 265 | 0.530 | | | | 9.194 | |
| | 634 | 326 | 206 | 0.544 | | | | 16.888 | |
| G10 | 907 | 734 | 395 | 0.445 | 0.492 | 0.046 | 0.094 | -19.830 | -1.116 |
| | 1100 | 602 | 343 | 0.538 | | | | 10.796 | |
| | 499 | 316 | 200 | 0.492 | | | | 5.685 | |
| H10 | 701 | 472 | 268 | 0.486 | 0.522 | 0.031 | 0.059 | -12.454 | 4.903 |
| | 1160 | 642 | 341 | 0.541 | | | | 11.496 | |
| | 410 | 234 | 118 | 0.538 | | | | 15.667 | |
# A10-H10
| - | + | C1 | D1 |
| --- | --- | --- | --- |
| A | B | C | D | E | F | G | H |
| --- | --- | --- | --- | --- | --- | --- | --- |
| Whole |
| --- |
| |
| CTD |
| NTD |

## Slide 13
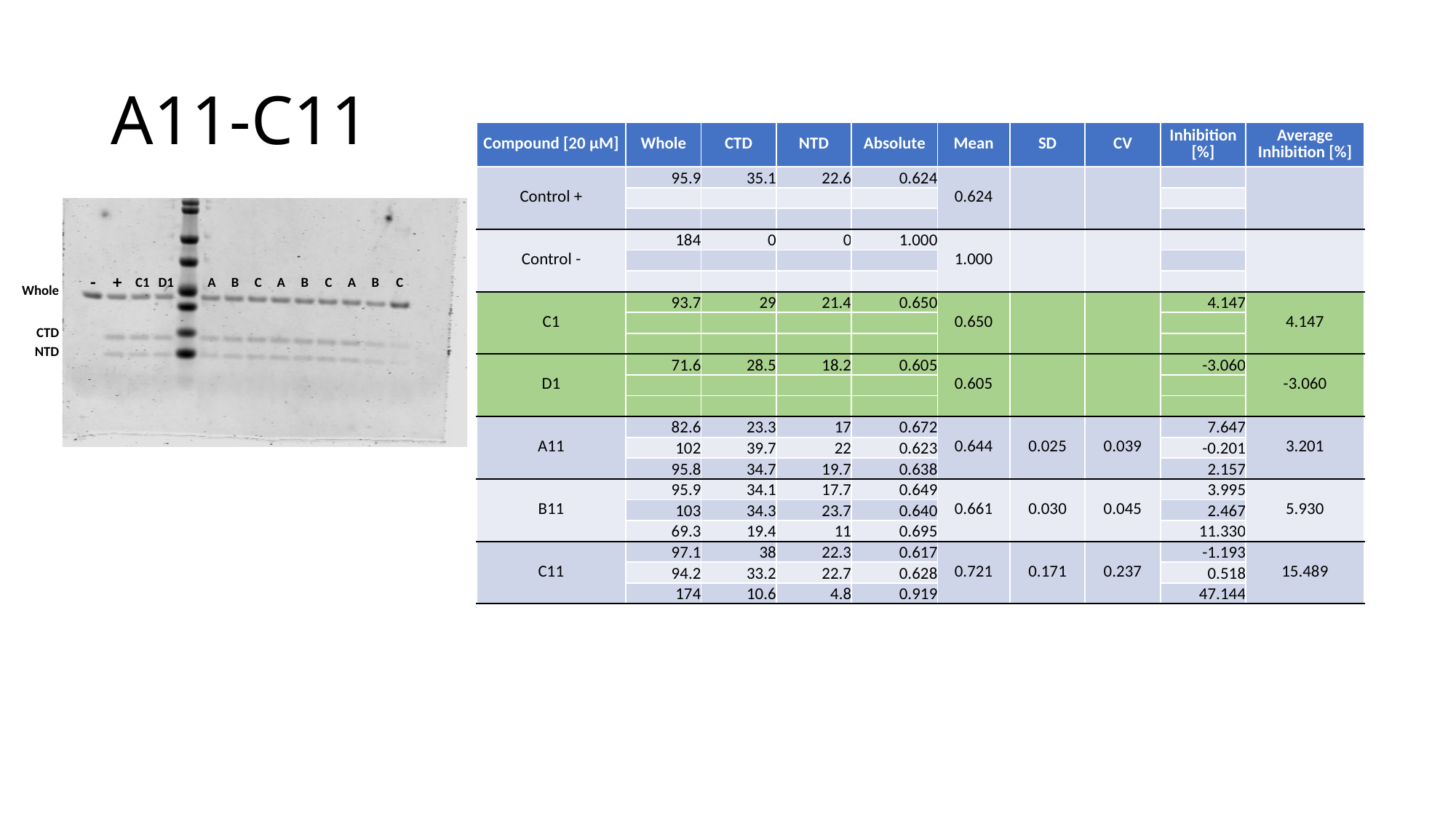

# A11-C11
| Compound [20 µM] | Whole | CTD | NTD | Absolute | Mean | SD | CV | Inhibition [%] | Average Inhibition [%] |
| --- | --- | --- | --- | --- | --- | --- | --- | --- | --- |
| Control + | 95.9 | 35.1 | 22.6 | 0.624 | 0.624 | | | | |
| | | | | | | | | | |
| | | | | | | | | | |
| Control - | 184 | 0 | 0 | 1.000 | 1.000 | | | | |
| | | | | | | | | | |
| | | | | | | | | | |
| C1 | 93.7 | 29 | 21.4 | 0.650 | 0.650 | | | 4.147 | 4.147 |
| | | | | | | | | | |
| | | | | | | | | | |
| D1 | 71.6 | 28.5 | 18.2 | 0.605 | 0.605 | | | -3.060 | -3.060 |
| | | | | | | | | | |
| | | | | | | | | | |
| A11 | 82.6 | 23.3 | 17 | 0.672 | 0.644 | 0.025 | 0.039 | 7.647 | 3.201 |
| | 102 | 39.7 | 22 | 0.623 | | | | -0.201 | |
| | 95.8 | 34.7 | 19.7 | 0.638 | | | | 2.157 | |
| B11 | 95.9 | 34.1 | 17.7 | 0.649 | 0.661 | 0.030 | 0.045 | 3.995 | 5.930 |
| | 103 | 34.3 | 23.7 | 0.640 | | | | 2.467 | |
| | 69.3 | 19.4 | 11 | 0.695 | | | | 11.330 | |
| C11 | 97.1 | 38 | 22.3 | 0.617 | 0.721 | 0.171 | 0.237 | -1.193 | 15.489 |
| | 94.2 | 33.2 | 22.7 | 0.628 | | | | 0.518 | |
| | 174 | 10.6 | 4.8 | 0.919 | | | | 47.144 | |
| - | + | C1 | D1 |
| --- | --- | --- | --- |
| A | B | C | A | B | C | A | B | C |
| --- | --- | --- | --- | --- | --- | --- | --- | --- |
| Whole |
| --- |
| |
| CTD |
| NTD |

## Slide 14
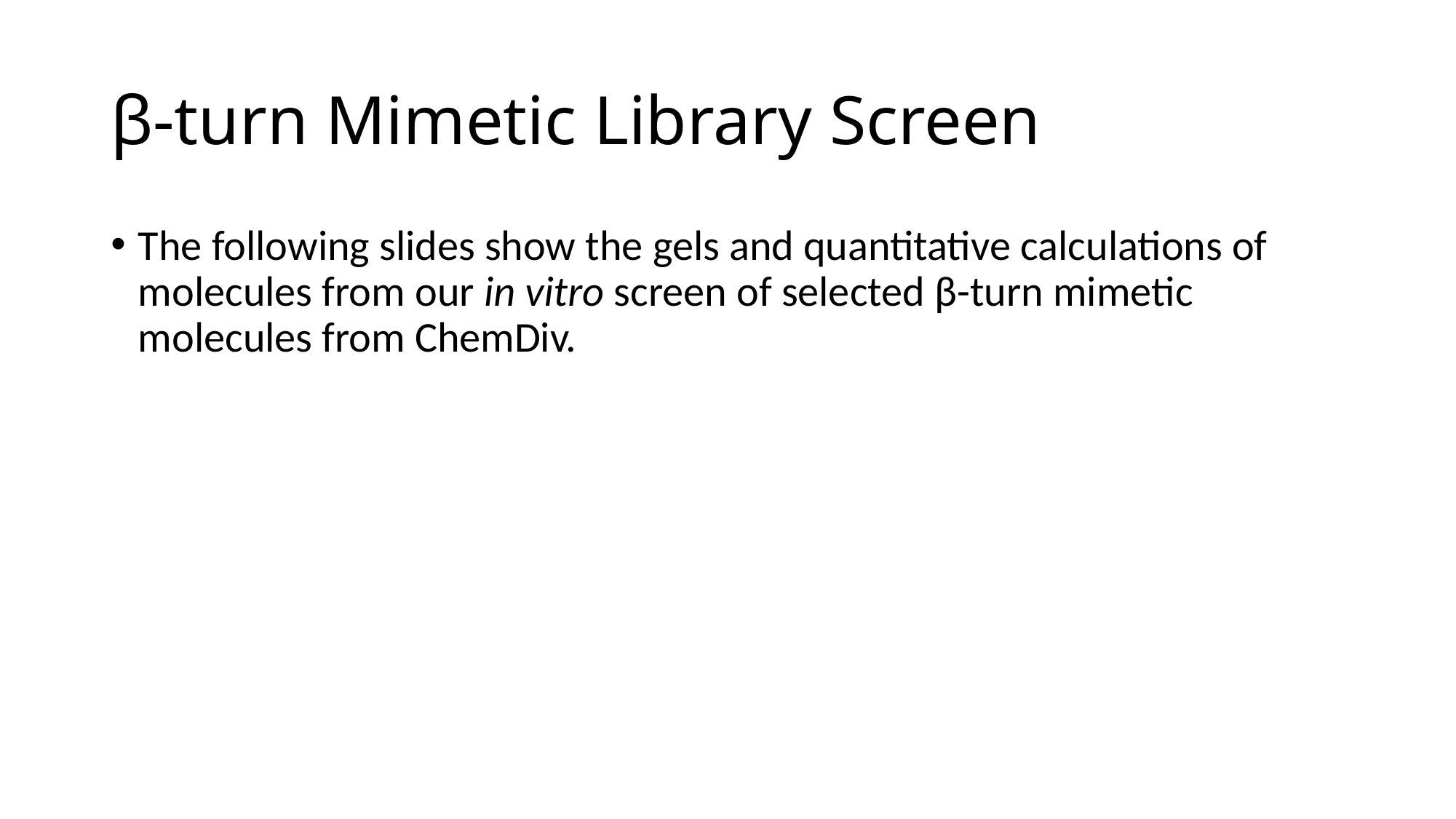

# β-turn Mimetic Library Screen
The following slides show the gels and quantitative calculations of molecules from our in vitro screen of selected β-turn mimetic molecules from ChemDiv.

## Slide 15
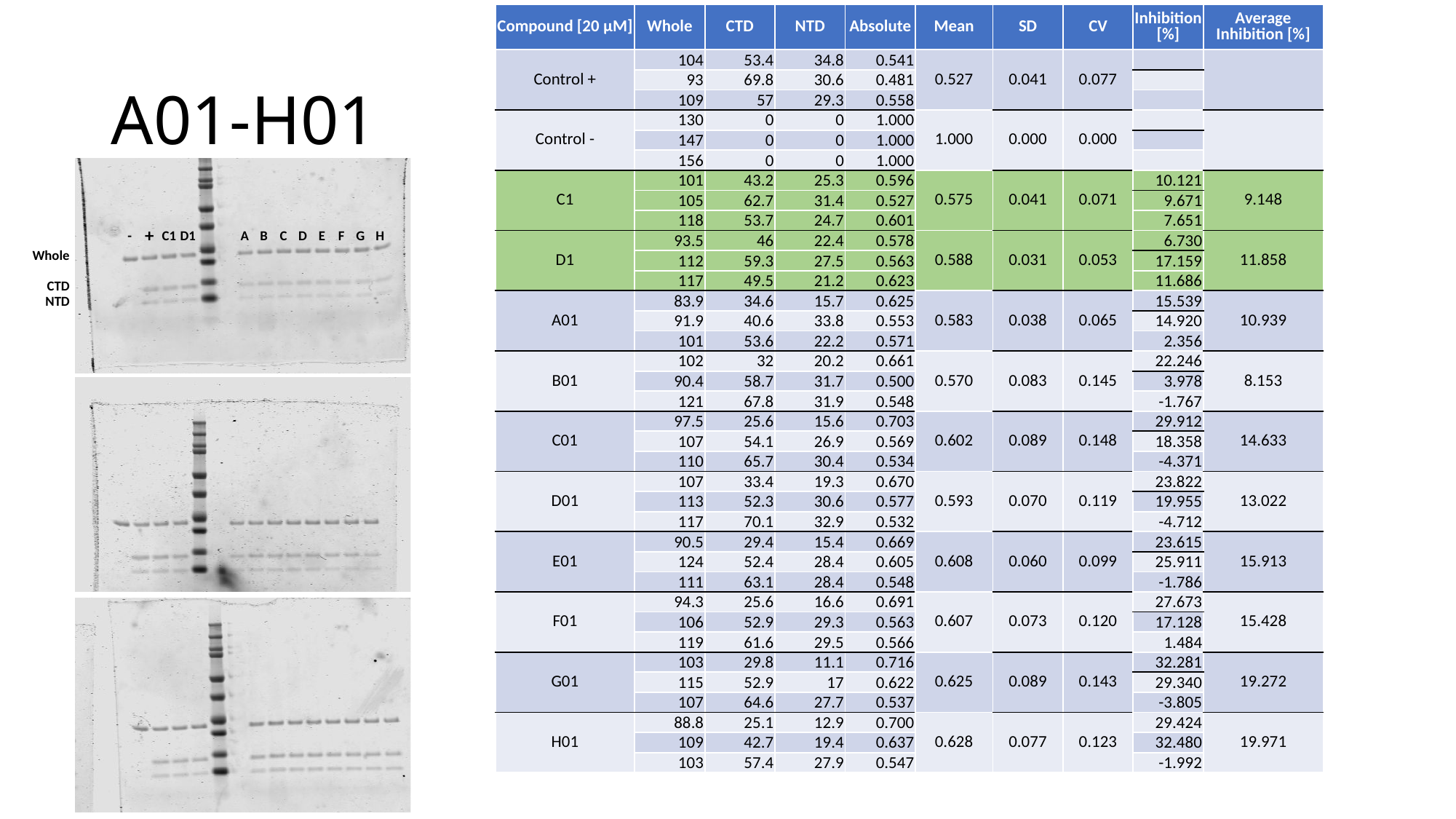

| Compound [20 µM] | Whole | CTD | NTD | Absolute | Mean | SD | CV | Inhibition [%] | Average Inhibition [%] |
| --- | --- | --- | --- | --- | --- | --- | --- | --- | --- |
| Control + | 104 | 53.4 | 34.8 | 0.541 | 0.527 | 0.041 | 0.077 | | |
| | 93 | 69.8 | 30.6 | 0.481 | | | | | |
| | 109 | 57 | 29.3 | 0.558 | | | | | |
| Control - | 130 | 0 | 0 | 1.000 | 1.000 | 0.000 | 0.000 | | |
| | 147 | 0 | 0 | 1.000 | | | | | |
| | 156 | 0 | 0 | 1.000 | | | | | |
| C1 | 101 | 43.2 | 25.3 | 0.596 | 0.575 | 0.041 | 0.071 | 10.121 | 9.148 |
| | 105 | 62.7 | 31.4 | 0.527 | | | | 9.671 | |
| | 118 | 53.7 | 24.7 | 0.601 | | | | 7.651 | |
| D1 | 93.5 | 46 | 22.4 | 0.578 | 0.588 | 0.031 | 0.053 | 6.730 | 11.858 |
| | 112 | 59.3 | 27.5 | 0.563 | | | | 17.159 | |
| | 117 | 49.5 | 21.2 | 0.623 | | | | 11.686 | |
| A01 | 83.9 | 34.6 | 15.7 | 0.625 | 0.583 | 0.038 | 0.065 | 15.539 | 10.939 |
| | 91.9 | 40.6 | 33.8 | 0.553 | | | | 14.920 | |
| | 101 | 53.6 | 22.2 | 0.571 | | | | 2.356 | |
| B01 | 102 | 32 | 20.2 | 0.661 | 0.570 | 0.083 | 0.145 | 22.246 | 8.153 |
| | 90.4 | 58.7 | 31.7 | 0.500 | | | | 3.978 | |
| | 121 | 67.8 | 31.9 | 0.548 | | | | -1.767 | |
| C01 | 97.5 | 25.6 | 15.6 | 0.703 | 0.602 | 0.089 | 0.148 | 29.912 | 14.633 |
| | 107 | 54.1 | 26.9 | 0.569 | | | | 18.358 | |
| | 110 | 65.7 | 30.4 | 0.534 | | | | -4.371 | |
| D01 | 107 | 33.4 | 19.3 | 0.670 | 0.593 | 0.070 | 0.119 | 23.822 | 13.022 |
| | 113 | 52.3 | 30.6 | 0.577 | | | | 19.955 | |
| | 117 | 70.1 | 32.9 | 0.532 | | | | -4.712 | |
| E01 | 90.5 | 29.4 | 15.4 | 0.669 | 0.608 | 0.060 | 0.099 | 23.615 | 15.913 |
| | 124 | 52.4 | 28.4 | 0.605 | | | | 25.911 | |
| | 111 | 63.1 | 28.4 | 0.548 | | | | -1.786 | |
| F01 | 94.3 | 25.6 | 16.6 | 0.691 | 0.607 | 0.073 | 0.120 | 27.673 | 15.428 |
| | 106 | 52.9 | 29.3 | 0.563 | | | | 17.128 | |
| | 119 | 61.6 | 29.5 | 0.566 | | | | 1.484 | |
| G01 | 103 | 29.8 | 11.1 | 0.716 | 0.625 | 0.089 | 0.143 | 32.281 | 19.272 |
| | 115 | 52.9 | 17 | 0.622 | | | | 29.340 | |
| | 107 | 64.6 | 27.7 | 0.537 | | | | -3.805 | |
| H01 | 88.8 | 25.1 | 12.9 | 0.700 | 0.628 | 0.077 | 0.123 | 29.424 | 19.971 |
| | 109 | 42.7 | 19.4 | 0.637 | | | | 32.480 | |
| | 103 | 57.4 | 27.9 | 0.547 | | | | -1.992 | |
# A01-H01
| A | B | C | D | E | F | G | H |
| --- | --- | --- | --- | --- | --- | --- | --- |
| - | + | C1 | D1 |
| --- | --- | --- | --- |
| Whole |
| --- |
| |
| CTD |
| NTD |

## Slide 16
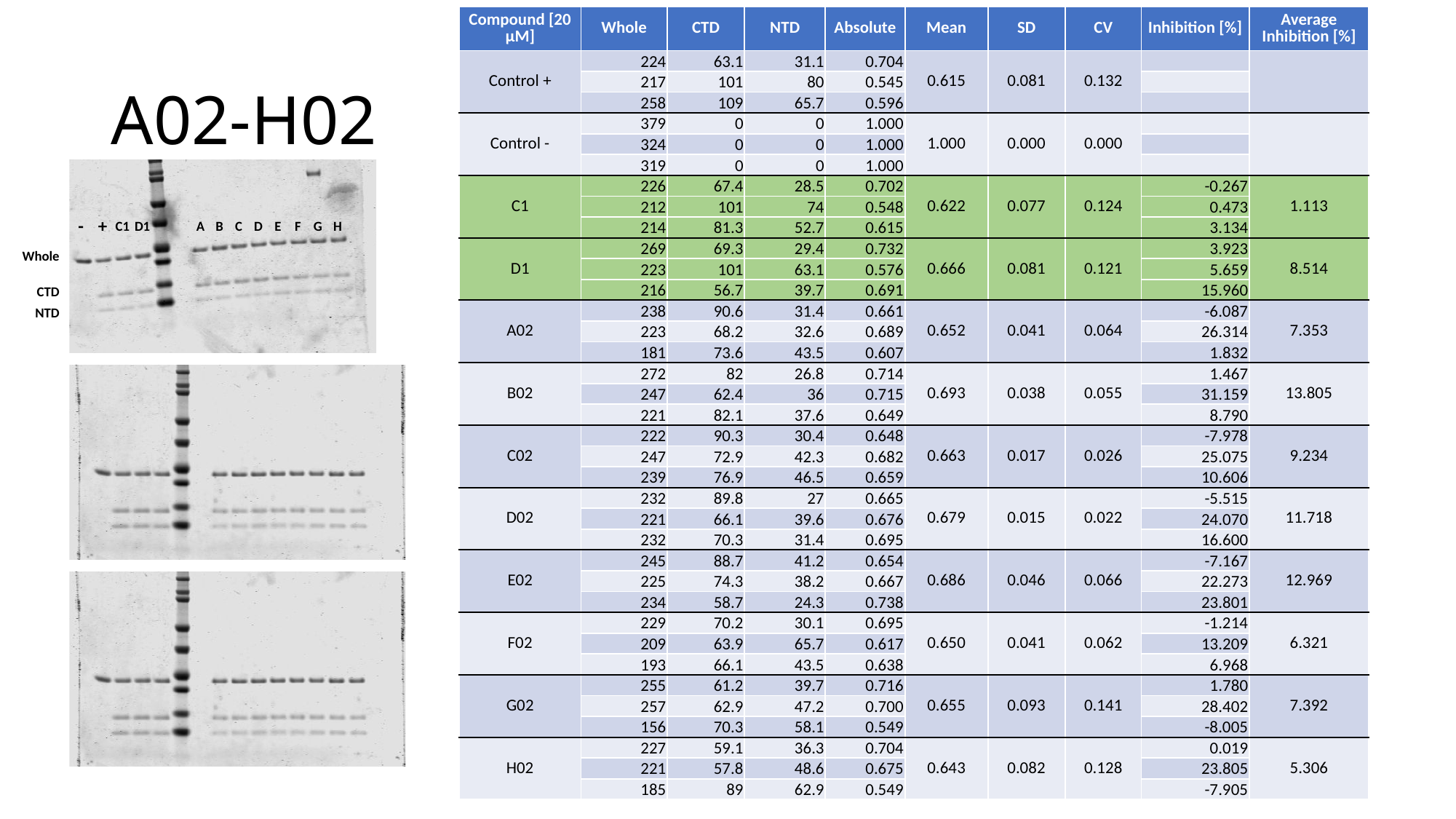

| Compound [20 µM] | Whole | CTD | NTD | Absolute | Mean | SD | CV | Inhibition [%] | Average Inhibition [%] |
| --- | --- | --- | --- | --- | --- | --- | --- | --- | --- |
| Control + | 224 | 63.1 | 31.1 | 0.704 | 0.615 | 0.081 | 0.132 | | |
| | 217 | 101 | 80 | 0.545 | | | | | |
| | 258 | 109 | 65.7 | 0.596 | | | | | |
| Control - | 379 | 0 | 0 | 1.000 | 1.000 | 0.000 | 0.000 | | |
| | 324 | 0 | 0 | 1.000 | | | | | |
| | 319 | 0 | 0 | 1.000 | | | | | |
| C1 | 226 | 67.4 | 28.5 | 0.702 | 0.622 | 0.077 | 0.124 | -0.267 | 1.113 |
| | 212 | 101 | 74 | 0.548 | | | | 0.473 | |
| | 214 | 81.3 | 52.7 | 0.615 | | | | 3.134 | |
| D1 | 269 | 69.3 | 29.4 | 0.732 | 0.666 | 0.081 | 0.121 | 3.923 | 8.514 |
| | 223 | 101 | 63.1 | 0.576 | | | | 5.659 | |
| | 216 | 56.7 | 39.7 | 0.691 | | | | 15.960 | |
| A02 | 238 | 90.6 | 31.4 | 0.661 | 0.652 | 0.041 | 0.064 | -6.087 | 7.353 |
| | 223 | 68.2 | 32.6 | 0.689 | | | | 26.314 | |
| | 181 | 73.6 | 43.5 | 0.607 | | | | 1.832 | |
| B02 | 272 | 82 | 26.8 | 0.714 | 0.693 | 0.038 | 0.055 | 1.467 | 13.805 |
| | 247 | 62.4 | 36 | 0.715 | | | | 31.159 | |
| | 221 | 82.1 | 37.6 | 0.649 | | | | 8.790 | |
| C02 | 222 | 90.3 | 30.4 | 0.648 | 0.663 | 0.017 | 0.026 | -7.978 | 9.234 |
| | 247 | 72.9 | 42.3 | 0.682 | | | | 25.075 | |
| | 239 | 76.9 | 46.5 | 0.659 | | | | 10.606 | |
| D02 | 232 | 89.8 | 27 | 0.665 | 0.679 | 0.015 | 0.022 | -5.515 | 11.718 |
| | 221 | 66.1 | 39.6 | 0.676 | | | | 24.070 | |
| | 232 | 70.3 | 31.4 | 0.695 | | | | 16.600 | |
| E02 | 245 | 88.7 | 41.2 | 0.654 | 0.686 | 0.046 | 0.066 | -7.167 | 12.969 |
| | 225 | 74.3 | 38.2 | 0.667 | | | | 22.273 | |
| | 234 | 58.7 | 24.3 | 0.738 | | | | 23.801 | |
| F02 | 229 | 70.2 | 30.1 | 0.695 | 0.650 | 0.041 | 0.062 | -1.214 | 6.321 |
| | 209 | 63.9 | 65.7 | 0.617 | | | | 13.209 | |
| | 193 | 66.1 | 43.5 | 0.638 | | | | 6.968 | |
| G02 | 255 | 61.2 | 39.7 | 0.716 | 0.655 | 0.093 | 0.141 | 1.780 | 7.392 |
| | 257 | 62.9 | 47.2 | 0.700 | | | | 28.402 | |
| | 156 | 70.3 | 58.1 | 0.549 | | | | -8.005 | |
| H02 | 227 | 59.1 | 36.3 | 0.704 | 0.643 | 0.082 | 0.128 | 0.019 | 5.306 |
| | 221 | 57.8 | 48.6 | 0.675 | | | | 23.805 | |
| | 185 | 89 | 62.9 | 0.549 | | | | -7.905 | |
# A02-H02
| - | + | C1 | D1 |
| --- | --- | --- | --- |
| A | B | C | D | E | F | G | H |
| --- | --- | --- | --- | --- | --- | --- | --- |
| Whole |
| --- |
| |
| CTD |
| NTD |

## Slide 17
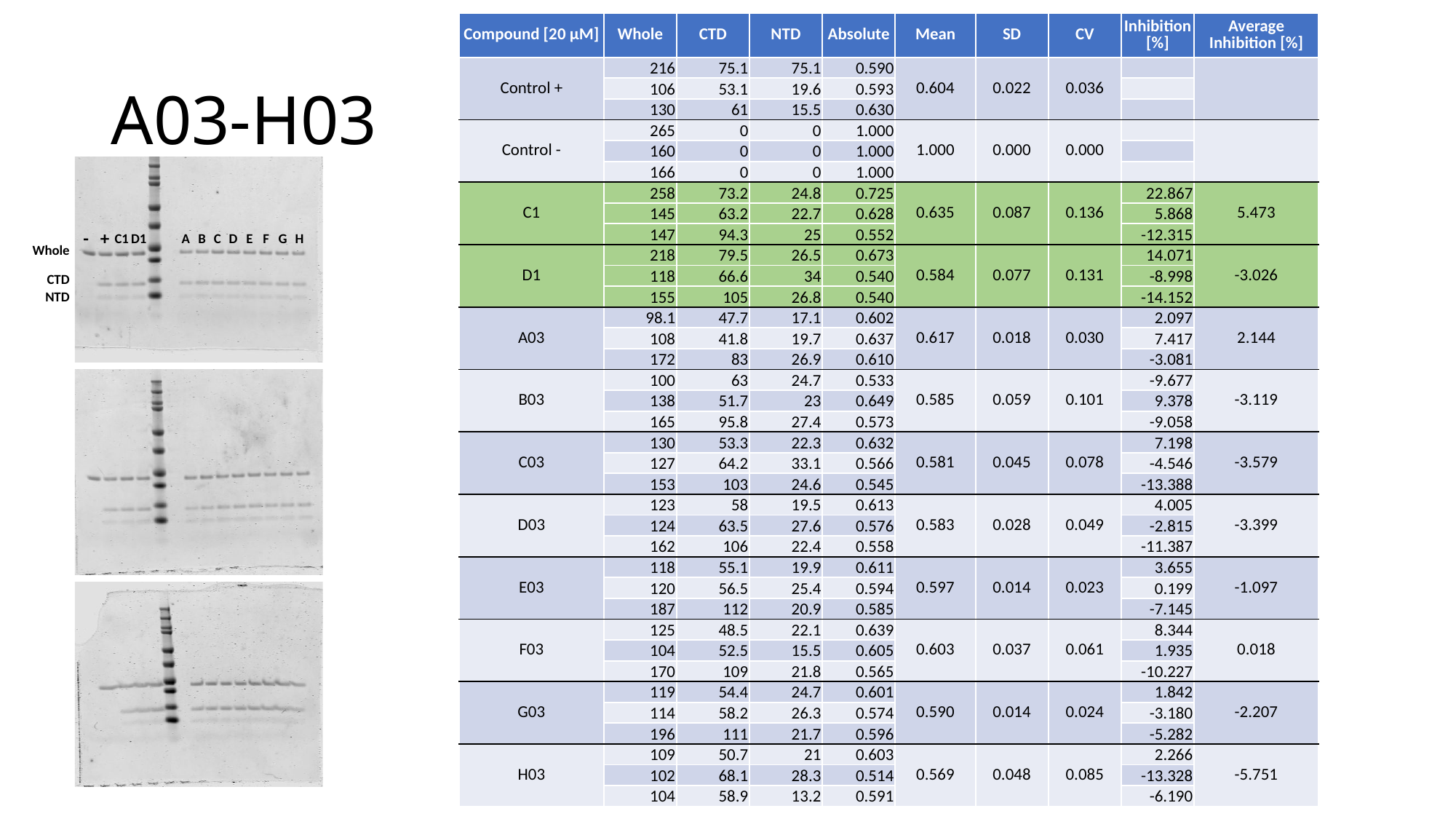

| Compound [20 µM] | Whole | CTD | NTD | Absolute | Mean | SD | CV | Inhibition [%] | Average Inhibition [%] |
| --- | --- | --- | --- | --- | --- | --- | --- | --- | --- |
| Control + | 216 | 75.1 | 75.1 | 0.590 | 0.604 | 0.022 | 0.036 | | |
| | 106 | 53.1 | 19.6 | 0.593 | | | | | |
| | 130 | 61 | 15.5 | 0.630 | | | | | |
| Control - | 265 | 0 | 0 | 1.000 | 1.000 | 0.000 | 0.000 | | |
| | 160 | 0 | 0 | 1.000 | | | | | |
| | 166 | 0 | 0 | 1.000 | | | | | |
| C1 | 258 | 73.2 | 24.8 | 0.725 | 0.635 | 0.087 | 0.136 | 22.867 | 5.473 |
| | 145 | 63.2 | 22.7 | 0.628 | | | | 5.868 | |
| | 147 | 94.3 | 25 | 0.552 | | | | -12.315 | |
| D1 | 218 | 79.5 | 26.5 | 0.673 | 0.584 | 0.077 | 0.131 | 14.071 | -3.026 |
| | 118 | 66.6 | 34 | 0.540 | | | | -8.998 | |
| | 155 | 105 | 26.8 | 0.540 | | | | -14.152 | |
| A03 | 98.1 | 47.7 | 17.1 | 0.602 | 0.617 | 0.018 | 0.030 | 2.097 | 2.144 |
| | 108 | 41.8 | 19.7 | 0.637 | | | | 7.417 | |
| | 172 | 83 | 26.9 | 0.610 | | | | -3.081 | |
| B03 | 100 | 63 | 24.7 | 0.533 | 0.585 | 0.059 | 0.101 | -9.677 | -3.119 |
| | 138 | 51.7 | 23 | 0.649 | | | | 9.378 | |
| | 165 | 95.8 | 27.4 | 0.573 | | | | -9.058 | |
| C03 | 130 | 53.3 | 22.3 | 0.632 | 0.581 | 0.045 | 0.078 | 7.198 | -3.579 |
| | 127 | 64.2 | 33.1 | 0.566 | | | | -4.546 | |
| | 153 | 103 | 24.6 | 0.545 | | | | -13.388 | |
| D03 | 123 | 58 | 19.5 | 0.613 | 0.583 | 0.028 | 0.049 | 4.005 | -3.399 |
| | 124 | 63.5 | 27.6 | 0.576 | | | | -2.815 | |
| | 162 | 106 | 22.4 | 0.558 | | | | -11.387 | |
| E03 | 118 | 55.1 | 19.9 | 0.611 | 0.597 | 0.014 | 0.023 | 3.655 | -1.097 |
| | 120 | 56.5 | 25.4 | 0.594 | | | | 0.199 | |
| | 187 | 112 | 20.9 | 0.585 | | | | -7.145 | |
| F03 | 125 | 48.5 | 22.1 | 0.639 | 0.603 | 0.037 | 0.061 | 8.344 | 0.018 |
| | 104 | 52.5 | 15.5 | 0.605 | | | | 1.935 | |
| | 170 | 109 | 21.8 | 0.565 | | | | -10.227 | |
| G03 | 119 | 54.4 | 24.7 | 0.601 | 0.590 | 0.014 | 0.024 | 1.842 | -2.207 |
| | 114 | 58.2 | 26.3 | 0.574 | | | | -3.180 | |
| | 196 | 111 | 21.7 | 0.596 | | | | -5.282 | |
| H03 | 109 | 50.7 | 21 | 0.603 | 0.569 | 0.048 | 0.085 | 2.266 | -5.751 |
| | 102 | 68.1 | 28.3 | 0.514 | | | | -13.328 | |
| | 104 | 58.9 | 13.2 | 0.591 | | | | -6.190 | |
# A03-H03
| - | + | C1 | D1 |
| --- | --- | --- | --- |
| A | B | C | D | E | F | G | H |
| --- | --- | --- | --- | --- | --- | --- | --- |
| Whole |
| --- |
| |
| CTD |
| NTD |

## Slide 18
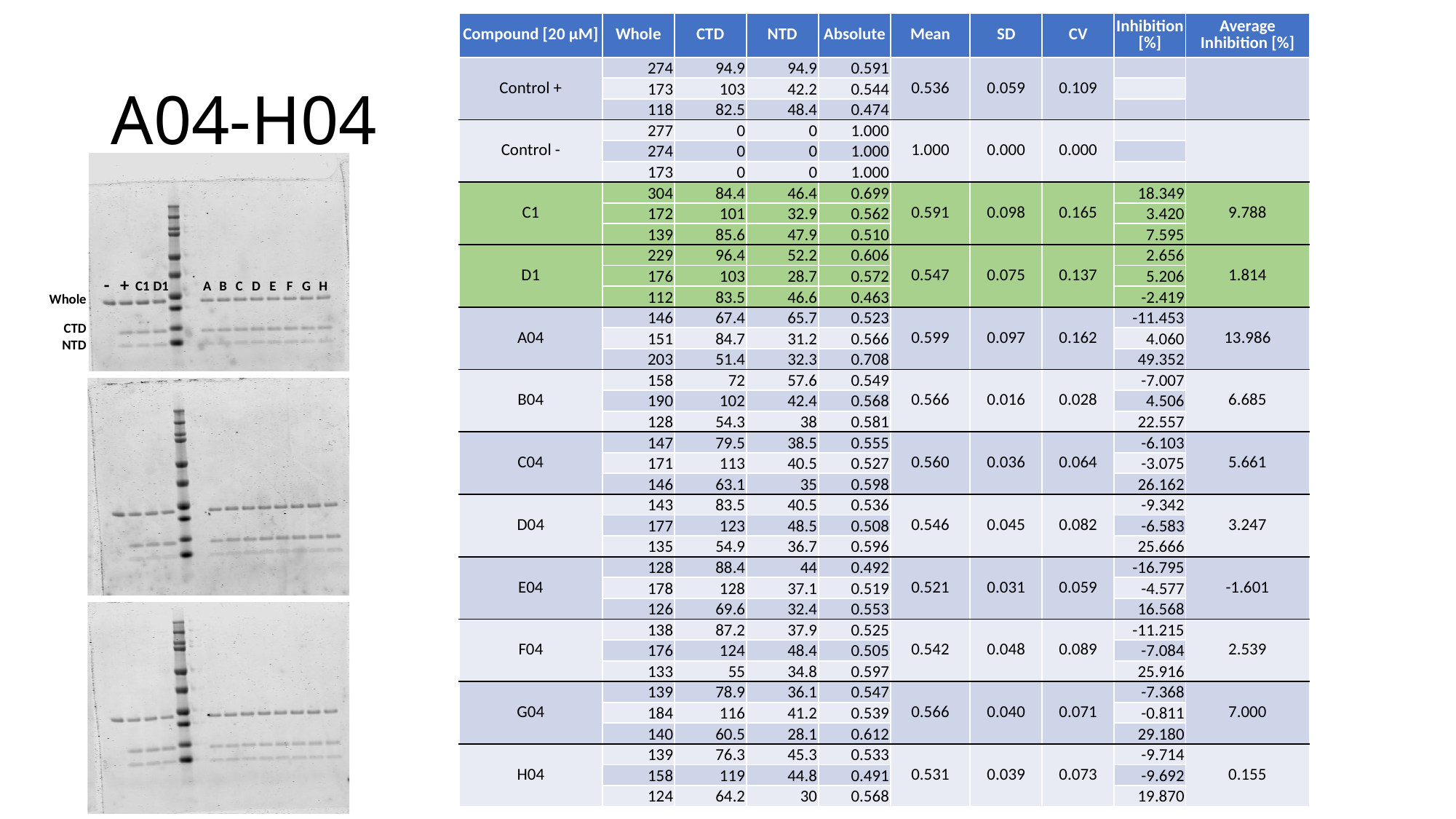

| Compound [20 µM] | Whole | CTD | NTD | Absolute | Mean | SD | CV | Inhibition [%] | Average Inhibition [%] |
| --- | --- | --- | --- | --- | --- | --- | --- | --- | --- |
| Control + | 274 | 94.9 | 94.9 | 0.591 | 0.536 | 0.059 | 0.109 | | |
| | 173 | 103 | 42.2 | 0.544 | | | | | |
| | 118 | 82.5 | 48.4 | 0.474 | | | | | |
| Control - | 277 | 0 | 0 | 1.000 | 1.000 | 0.000 | 0.000 | | |
| | 274 | 0 | 0 | 1.000 | | | | | |
| | 173 | 0 | 0 | 1.000 | | | | | |
| C1 | 304 | 84.4 | 46.4 | 0.699 | 0.591 | 0.098 | 0.165 | 18.349 | 9.788 |
| | 172 | 101 | 32.9 | 0.562 | | | | 3.420 | |
| | 139 | 85.6 | 47.9 | 0.510 | | | | 7.595 | |
| D1 | 229 | 96.4 | 52.2 | 0.606 | 0.547 | 0.075 | 0.137 | 2.656 | 1.814 |
| | 176 | 103 | 28.7 | 0.572 | | | | 5.206 | |
| | 112 | 83.5 | 46.6 | 0.463 | | | | -2.419 | |
| A04 | 146 | 67.4 | 65.7 | 0.523 | 0.599 | 0.097 | 0.162 | -11.453 | 13.986 |
| | 151 | 84.7 | 31.2 | 0.566 | | | | 4.060 | |
| | 203 | 51.4 | 32.3 | 0.708 | | | | 49.352 | |
| B04 | 158 | 72 | 57.6 | 0.549 | 0.566 | 0.016 | 0.028 | -7.007 | 6.685 |
| | 190 | 102 | 42.4 | 0.568 | | | | 4.506 | |
| | 128 | 54.3 | 38 | 0.581 | | | | 22.557 | |
| C04 | 147 | 79.5 | 38.5 | 0.555 | 0.560 | 0.036 | 0.064 | -6.103 | 5.661 |
| | 171 | 113 | 40.5 | 0.527 | | | | -3.075 | |
| | 146 | 63.1 | 35 | 0.598 | | | | 26.162 | |
| D04 | 143 | 83.5 | 40.5 | 0.536 | 0.546 | 0.045 | 0.082 | -9.342 | 3.247 |
| | 177 | 123 | 48.5 | 0.508 | | | | -6.583 | |
| | 135 | 54.9 | 36.7 | 0.596 | | | | 25.666 | |
| E04 | 128 | 88.4 | 44 | 0.492 | 0.521 | 0.031 | 0.059 | -16.795 | -1.601 |
| | 178 | 128 | 37.1 | 0.519 | | | | -4.577 | |
| | 126 | 69.6 | 32.4 | 0.553 | | | | 16.568 | |
| F04 | 138 | 87.2 | 37.9 | 0.525 | 0.542 | 0.048 | 0.089 | -11.215 | 2.539 |
| | 176 | 124 | 48.4 | 0.505 | | | | -7.084 | |
| | 133 | 55 | 34.8 | 0.597 | | | | 25.916 | |
| G04 | 139 | 78.9 | 36.1 | 0.547 | 0.566 | 0.040 | 0.071 | -7.368 | 7.000 |
| | 184 | 116 | 41.2 | 0.539 | | | | -0.811 | |
| | 140 | 60.5 | 28.1 | 0.612 | | | | 29.180 | |
| H04 | 139 | 76.3 | 45.3 | 0.533 | 0.531 | 0.039 | 0.073 | -9.714 | 0.155 |
| | 158 | 119 | 44.8 | 0.491 | | | | -9.692 | |
| | 124 | 64.2 | 30 | 0.568 | | | | 19.870 | |
# A04-H04
| - | + | C1 | D1 |
| --- | --- | --- | --- |
| A | B | C | D | E | F | G | H |
| --- | --- | --- | --- | --- | --- | --- | --- |
| Whole |
| --- |
| |
| CTD |
| NTD |

## Slide 19
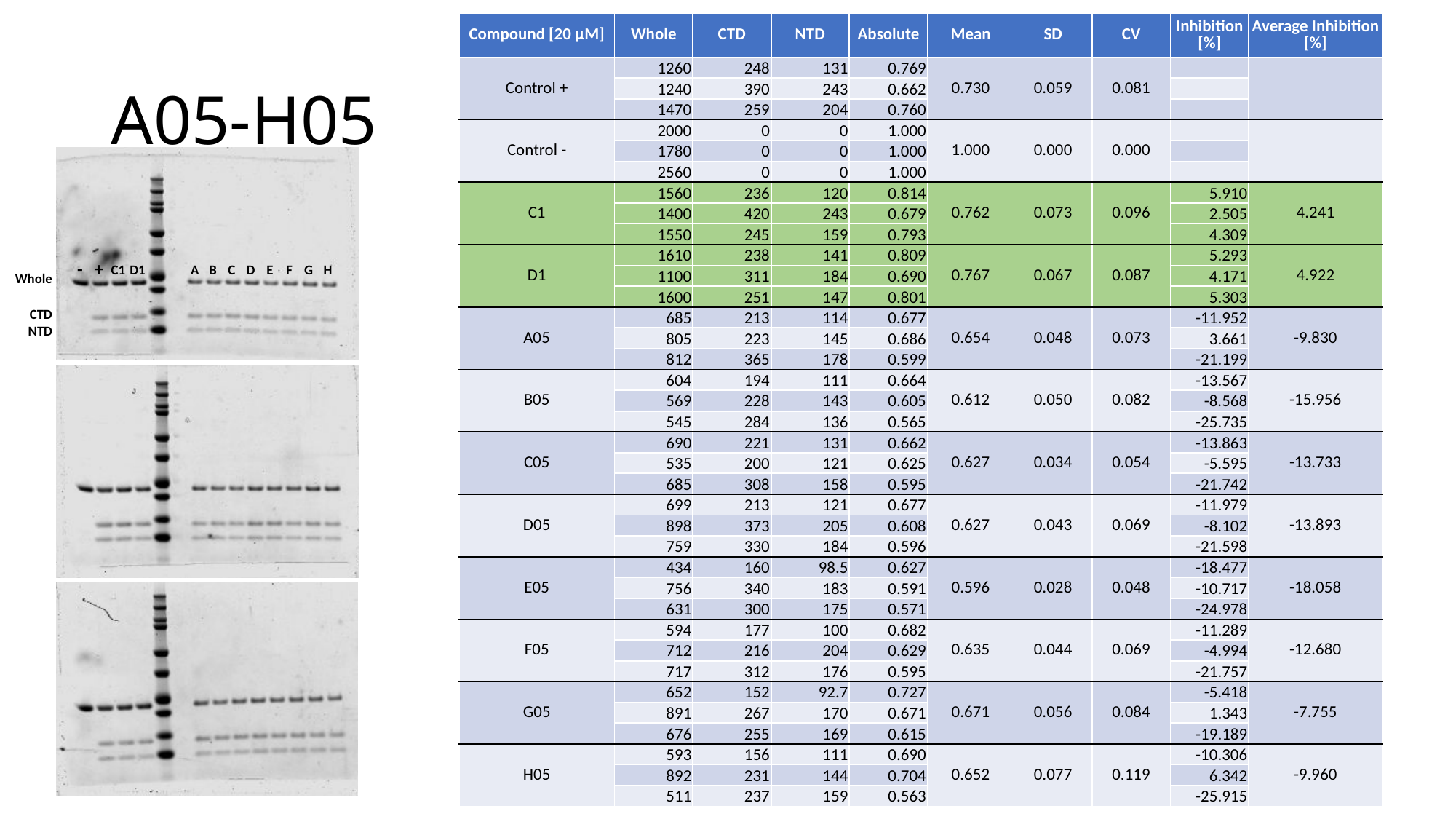

| Compound [20 µM] | Whole | CTD | NTD | Absolute | Mean | SD | CV | Inhibition [%] | Average Inhibition [%] |
| --- | --- | --- | --- | --- | --- | --- | --- | --- | --- |
| Control + | 1260 | 248 | 131 | 0.769 | 0.730 | 0.059 | 0.081 | | |
| | 1240 | 390 | 243 | 0.662 | | | | | |
| | 1470 | 259 | 204 | 0.760 | | | | | |
| Control - | 2000 | 0 | 0 | 1.000 | 1.000 | 0.000 | 0.000 | | |
| | 1780 | 0 | 0 | 1.000 | | | | | |
| | 2560 | 0 | 0 | 1.000 | | | | | |
| C1 | 1560 | 236 | 120 | 0.814 | 0.762 | 0.073 | 0.096 | 5.910 | 4.241 |
| | 1400 | 420 | 243 | 0.679 | | | | 2.505 | |
| | 1550 | 245 | 159 | 0.793 | | | | 4.309 | |
| D1 | 1610 | 238 | 141 | 0.809 | 0.767 | 0.067 | 0.087 | 5.293 | 4.922 |
| | 1100 | 311 | 184 | 0.690 | | | | 4.171 | |
| | 1600 | 251 | 147 | 0.801 | | | | 5.303 | |
| A05 | 685 | 213 | 114 | 0.677 | 0.654 | 0.048 | 0.073 | -11.952 | -9.830 |
| | 805 | 223 | 145 | 0.686 | | | | 3.661 | |
| | 812 | 365 | 178 | 0.599 | | | | -21.199 | |
| B05 | 604 | 194 | 111 | 0.664 | 0.612 | 0.050 | 0.082 | -13.567 | -15.956 |
| | 569 | 228 | 143 | 0.605 | | | | -8.568 | |
| | 545 | 284 | 136 | 0.565 | | | | -25.735 | |
| C05 | 690 | 221 | 131 | 0.662 | 0.627 | 0.034 | 0.054 | -13.863 | -13.733 |
| | 535 | 200 | 121 | 0.625 | | | | -5.595 | |
| | 685 | 308 | 158 | 0.595 | | | | -21.742 | |
| D05 | 699 | 213 | 121 | 0.677 | 0.627 | 0.043 | 0.069 | -11.979 | -13.893 |
| | 898 | 373 | 205 | 0.608 | | | | -8.102 | |
| | 759 | 330 | 184 | 0.596 | | | | -21.598 | |
| E05 | 434 | 160 | 98.5 | 0.627 | 0.596 | 0.028 | 0.048 | -18.477 | -18.058 |
| | 756 | 340 | 183 | 0.591 | | | | -10.717 | |
| | 631 | 300 | 175 | 0.571 | | | | -24.978 | |
| F05 | 594 | 177 | 100 | 0.682 | 0.635 | 0.044 | 0.069 | -11.289 | -12.680 |
| | 712 | 216 | 204 | 0.629 | | | | -4.994 | |
| | 717 | 312 | 176 | 0.595 | | | | -21.757 | |
| G05 | 652 | 152 | 92.7 | 0.727 | 0.671 | 0.056 | 0.084 | -5.418 | -7.755 |
| | 891 | 267 | 170 | 0.671 | | | | 1.343 | |
| | 676 | 255 | 169 | 0.615 | | | | -19.189 | |
| H05 | 593 | 156 | 111 | 0.690 | 0.652 | 0.077 | 0.119 | -10.306 | -9.960 |
| | 892 | 231 | 144 | 0.704 | | | | 6.342 | |
| | 511 | 237 | 159 | 0.563 | | | | -25.915 | |
# A05-H05
| - | + | C1 | D1 |
| --- | --- | --- | --- |
| A | B | C | D | E | F | G | H |
| --- | --- | --- | --- | --- | --- | --- | --- |
| Whole |
| --- |
| |
| CTD |
| NTD |

## Slide 20
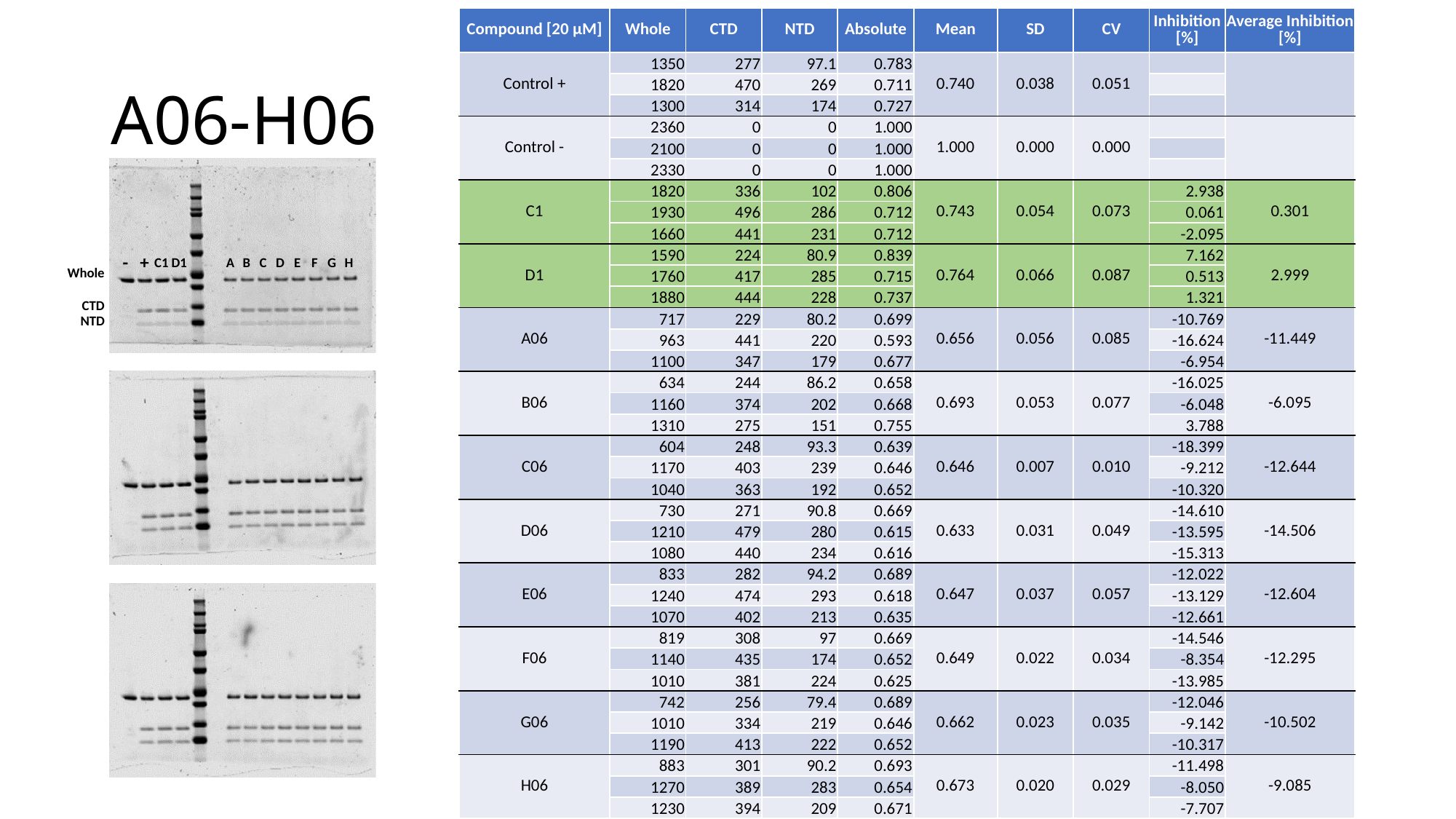

| Compound [20 µM] | Whole | CTD | NTD | Absolute | Mean | SD | CV | Inhibition [%] | Average Inhibition [%] |
| --- | --- | --- | --- | --- | --- | --- | --- | --- | --- |
| Control + | 1350 | 277 | 97.1 | 0.783 | 0.740 | 0.038 | 0.051 | | |
| | 1820 | 470 | 269 | 0.711 | | | | | |
| | 1300 | 314 | 174 | 0.727 | | | | | |
| Control - | 2360 | 0 | 0 | 1.000 | 1.000 | 0.000 | 0.000 | | |
| | 2100 | 0 | 0 | 1.000 | | | | | |
| | 2330 | 0 | 0 | 1.000 | | | | | |
| C1 | 1820 | 336 | 102 | 0.806 | 0.743 | 0.054 | 0.073 | 2.938 | 0.301 |
| | 1930 | 496 | 286 | 0.712 | | | | 0.061 | |
| | 1660 | 441 | 231 | 0.712 | | | | -2.095 | |
| D1 | 1590 | 224 | 80.9 | 0.839 | 0.764 | 0.066 | 0.087 | 7.162 | 2.999 |
| | 1760 | 417 | 285 | 0.715 | | | | 0.513 | |
| | 1880 | 444 | 228 | 0.737 | | | | 1.321 | |
| A06 | 717 | 229 | 80.2 | 0.699 | 0.656 | 0.056 | 0.085 | -10.769 | -11.449 |
| | 963 | 441 | 220 | 0.593 | | | | -16.624 | |
| | 1100 | 347 | 179 | 0.677 | | | | -6.954 | |
| B06 | 634 | 244 | 86.2 | 0.658 | 0.693 | 0.053 | 0.077 | -16.025 | -6.095 |
| | 1160 | 374 | 202 | 0.668 | | | | -6.048 | |
| | 1310 | 275 | 151 | 0.755 | | | | 3.788 | |
| C06 | 604 | 248 | 93.3 | 0.639 | 0.646 | 0.007 | 0.010 | -18.399 | -12.644 |
| | 1170 | 403 | 239 | 0.646 | | | | -9.212 | |
| | 1040 | 363 | 192 | 0.652 | | | | -10.320 | |
| D06 | 730 | 271 | 90.8 | 0.669 | 0.633 | 0.031 | 0.049 | -14.610 | -14.506 |
| | 1210 | 479 | 280 | 0.615 | | | | -13.595 | |
| | 1080 | 440 | 234 | 0.616 | | | | -15.313 | |
| E06 | 833 | 282 | 94.2 | 0.689 | 0.647 | 0.037 | 0.057 | -12.022 | -12.604 |
| | 1240 | 474 | 293 | 0.618 | | | | -13.129 | |
| | 1070 | 402 | 213 | 0.635 | | | | -12.661 | |
| F06 | 819 | 308 | 97 | 0.669 | 0.649 | 0.022 | 0.034 | -14.546 | -12.295 |
| | 1140 | 435 | 174 | 0.652 | | | | -8.354 | |
| | 1010 | 381 | 224 | 0.625 | | | | -13.985 | |
| G06 | 742 | 256 | 79.4 | 0.689 | 0.662 | 0.023 | 0.035 | -12.046 | -10.502 |
| | 1010 | 334 | 219 | 0.646 | | | | -9.142 | |
| | 1190 | 413 | 222 | 0.652 | | | | -10.317 | |
| H06 | 883 | 301 | 90.2 | 0.693 | 0.673 | 0.020 | 0.029 | -11.498 | -9.085 |
| | 1270 | 389 | 283 | 0.654 | | | | -8.050 | |
| | 1230 | 394 | 209 | 0.671 | | | | -7.707 | |
# A06-H06
| - | + | C1 | D1 |
| --- | --- | --- | --- |
| A | B | C | D | E | F | G | H |
| --- | --- | --- | --- | --- | --- | --- | --- |
| Whole |
| --- |
| |
| CTD |
| NTD |

## Slide 21
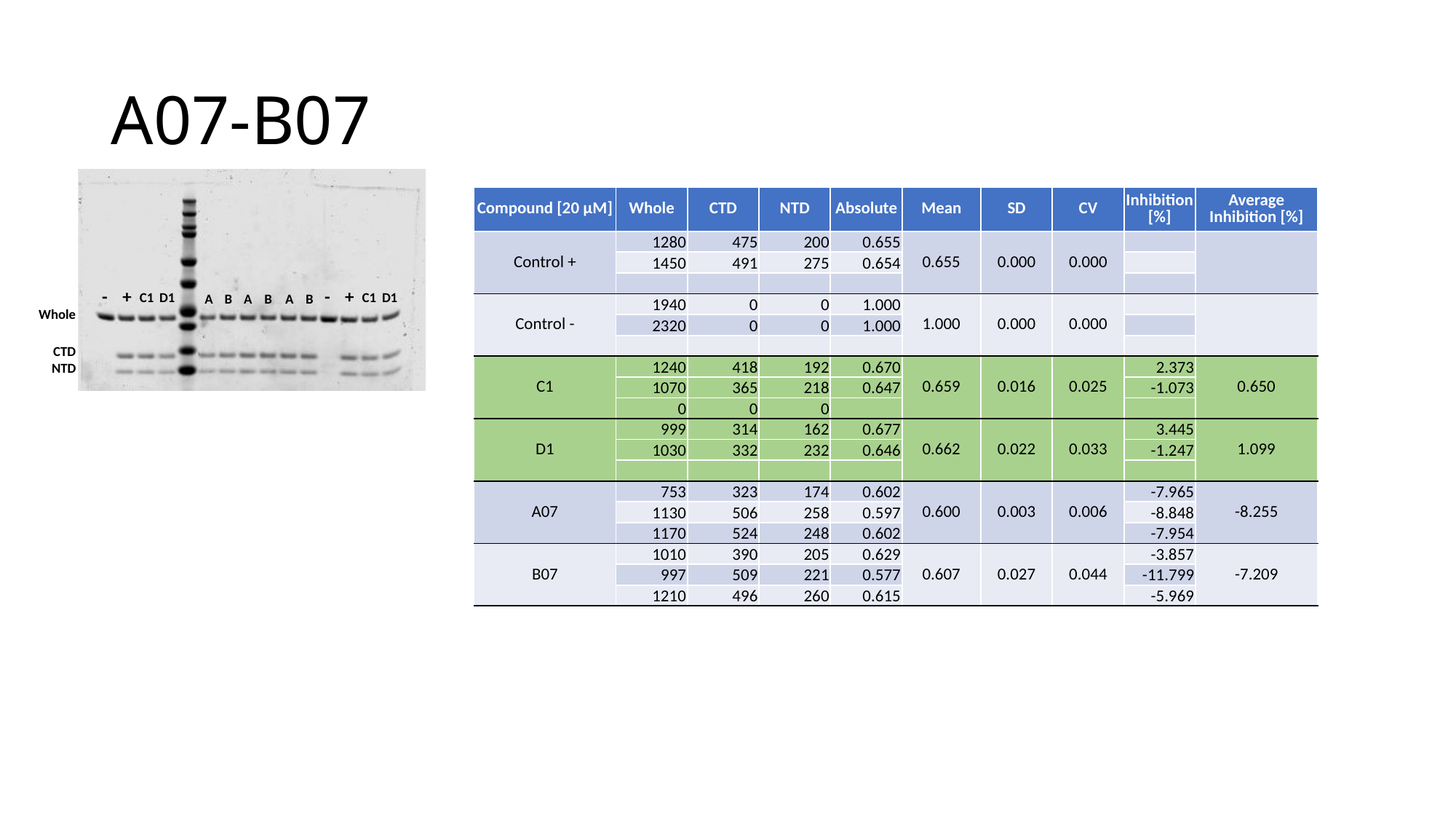

# A07-B07
| Compound [20 µM] | Whole | CTD | NTD | Absolute | Mean | SD | CV | Inhibition [%] | Average Inhibition [%] |
| --- | --- | --- | --- | --- | --- | --- | --- | --- | --- |
| Control + | 1280 | 475 | 200 | 0.655 | 0.655 | 0.000 | 0.000 | | |
| | 1450 | 491 | 275 | 0.654 | | | | | |
| | | | | | | | | | |
| Control - | 1940 | 0 | 0 | 1.000 | 1.000 | 0.000 | 0.000 | | |
| | 2320 | 0 | 0 | 1.000 | | | | | |
| | | | | | | | | | |
| C1 | 1240 | 418 | 192 | 0.670 | 0.659 | 0.016 | 0.025 | 2.373 | 0.650 |
| | 1070 | 365 | 218 | 0.647 | | | | -1.073 | |
| | 0 | 0 | 0 | | | | | | |
| D1 | 999 | 314 | 162 | 0.677 | 0.662 | 0.022 | 0.033 | 3.445 | 1.099 |
| | 1030 | 332 | 232 | 0.646 | | | | -1.247 | |
| | | | | | | | | | |
| A07 | 753 | 323 | 174 | 0.602 | 0.600 | 0.003 | 0.006 | -7.965 | -8.255 |
| | 1130 | 506 | 258 | 0.597 | | | | -8.848 | |
| | 1170 | 524 | 248 | 0.602 | | | | -7.954 | |
| B07 | 1010 | 390 | 205 | 0.629 | 0.607 | 0.027 | 0.044 | -3.857 | -7.209 |
| | 997 | 509 | 221 | 0.577 | | | | -11.799 | |
| | 1210 | 496 | 260 | 0.615 | | | | -5.969 | |
| - | + | C1 | D1 |
| --- | --- | --- | --- |
| - | + | C1 | D1 |
| --- | --- | --- | --- |
| A | B | A | B | A | B |
| --- | --- | --- | --- | --- | --- |
| Whole |
| --- |
| |
| CTD |
| NTD |
